# Construction of highly enantioenriched spirocyclopentaneoxindoles containing four consecutive stereocenters via thiourea-catalyzed asymmetric Michael–Henry cascade reactions

**DOI:** 10.3762/bjoc.13.131

**Published:** 2017-07-07

**Authors:** Yonglei Du, Jian Li, Kerong Chen, Chenglin Wu, Yu Zhou, Hong Liu

**Affiliations:** 1 Nano Science and Technology Institute, University of Science and Technology of China, 166 Ren Ai Road, Suzhou 215123, China; 2 CAS Key Laboratory of Receptor Research, Shanghai Institute of Materia Medica, Chinese Academy of Sciences, 555, Zu Chong Zhi Road, Shanghai 201203, China; 3 University of Chinese Academy of Sciences, NO.19A Yuquan Road, Beijing 100049, China

**Keywords:** asymmetric synthesis, four consecutive stereocenters, Michael–Henry cascade reactions, spirocyclopentaneoxindoles, thioureas

## Abstract

The thiourea-catalyzed asymmetric synthesis of highly enantioenriched spirocyclopentaneoxindoles containing chiral amide functional groups using simple 3-substituted oxindoles and nitrovinylacetamide as starting materials was achieved successfully. This protocol features operational simplicity, high atom economy, and high catalytic asymmetry, thus representing a versatile approach to the synthesis of highly enantioenriched spirocyclopentaneoxindoles.

## Introduction

**Figure 1:**
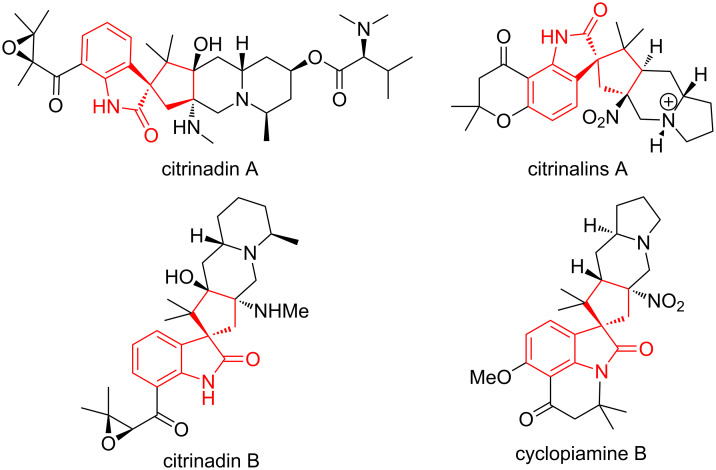
Representative spirooxindole natural products.

The spirocyclic oxindole core represents an important scaffold that is encountered frequently in many biologically active molecules and natural products (Figure 1) [1–19]. Despite many advances in asymmetric synthesis in the construction of heterocyclic spirooxindoles in the past decade [2,4,11,20–22], the development of general and practical strategies to obtain saturated spirocyclopentaneoxindoles containing multiple contiguous stereocenters remains challenging [23–26]. The medicinal properties of these frameworks mean that fast enrichment of spirooxindoles bearing diverse functional groups is of considerable importance.

**Scheme 1:**
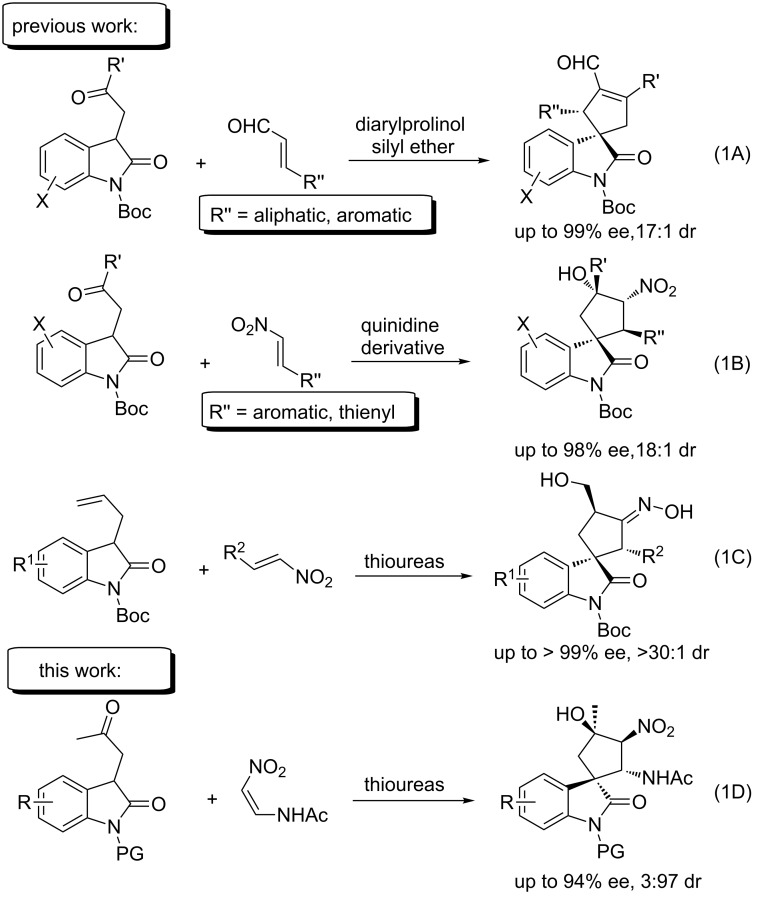
Construction of spirocyclopentaneoxindole scaffolds.

Recently, an increasing number of asymmetric catalysis strategies with chiral transition metals [27–33], organocatalysts such as secondary amines [34–36], nucleophilic phosphines [26,37–44], tertiary amines [45], *N*-heterocyclic carbenes (NHCs) [46–48], and cinchona alkaloid derivatives [25,28,49–50] have been used to construct successfully spirooxindole privileged scaffolds. However, most of them were devoted to exploring new catalyst systems to improve the reaction efficiency and selectivity [51–64], and studies extending the reaction scope using functionalized nitrovinylacetamides are rare. Chiral thioureas [28,38–39] have evolved as powerful hydrogen bonding catalysts for the asymmetric synthesis of spirocyclopentaneoxindoles, which have been demonstrated as acceptable but still considerably limited. Organocatalytic iminium–enamine cascade reactions [35] (Scheme 1, reaction 1A) and Michael–Henry cascade reactions [25] (Scheme 1, reaction 1B) reported by Barbas III's group involve the cyclization between α,β-unsaturated aldehydes and nitrostyrenes with 3-substituted oxindoles to generate the corresponding CHO- or NO_2_-substituted spirooxindole derivatives with good enantiomeric excess (ee) values, respectively. However, the utility of the reaction is limited to α,β-unsaturated aldehydes with aromatic/alkane substitutions and nitroolefins with aromatic substitutions. Additionally, Shao’s group (Scheme 1, reaction 1C) developed a one-pot thiourea-catalyzed Michael addition/intramolecular silyl nitronate-olefin cycloaddition (ISOC)/fragmentation sequence to produce highly enantioenriched spirocyclopentaneoxindoles containing an oxime functional group from easily accessible 3-allyl-substituted oxindoles and nitroolefins, which has received wide attention because of its high efficiency in constructing functionalized spirocyclopentaneoxindoles. However, lower temperatures are required (−30 °C) [50]. Therefore, it is highly desirable to develop novel and efficient methods to access directly various spirocycles. In our continuous endeavor to develop effective strategies to construct biologically active spirocyclic oxindoles [65–68], we have built successfully interesting spirooxindoles via an NHC-catalyzed [4 + 2] annulation involving an oxidative *γ*-carbon activation of common α,β-unsaturated aldehydes [68]. Herein, we report another effective asymmetric catalytic synthesis of saturated spirocyclopentaneoxindoles containing four consecutive stereocenters with 3-substituted oxindoles and nitrovinylacetamide using a bifunctional thiourea catalyst in good yields (up to 95%) with excellent diastereoselectivity (up to 3:97) and enantioselectivity (up to 94%) (Scheme 1, reaction 1D).

## Results and Discussion

To establish the optimal experimental conditions for the synthesis of spirocyclopentaneoxindoles, we chose 3-substituted oxindole **1a** and nitrovinylacetamide (**2a**) as the model substrates, and the results are summarized in Table 1. Initially, a variety of organocatalysts (**a–f**) were investigated in CH_2_Cl_2_ at −20 °C for 12 h to evaluate their ability to promote the transformation (Table 1, entries 1–6). When cinchona alkaloid-derived catalyst **a** and quinine-derived amine catalyst **b** were tested, however, poor yields or ee values were obtained, respectively (Table 1, entries 1 and 2). Further experiments showed that a bifunctional thiourea catalyst **d** was the most efficient for the synthesis of spirocyclic oxindole derivatives in good yields (80%) with excellent diastereoselectivity (8:92 dr), and moderate enantioselectivity (83% ee, Table 1, entries 3–6). Therefore, catalyst **d** was chosen as the optimal catalyst for further investigation (Table 1, entry 4). The reaction temperature was investigated, however elevated temperatures are detrimental (Table 1, entries 4, 12, 13). Different solvents, such as methanol, toluene, acetone, ether and chloroform were further screened. The results suggested that changing the solvent had an adverse effect on the ee value, and CH_2_Cl_2_ remained the best choice for this transformation (Table 1, entries 4, 7–11). Subsequently, we investigated some additives, for example, *p*-MeC_6_H_4_SO_3_H, trimethylsilyl chloride (TMSCl) and trifluoroacetic acid (TFA) for this catalytic system to increase the ee value of the target product; however, the use of the additives proved ineffective (Table 1, entries 15–17).

**Table 1:** Optimization for the reaction conditions^a^.

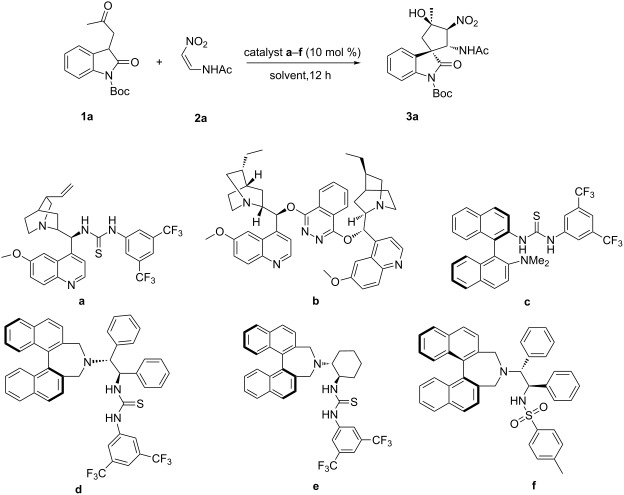						

entry	catalyst	solvent	additive	yield(%)^b^	dr^c^	ee (%)^d^

1	a	CH_2_Cl_2_	–	37	5:95	99
2	b	CH_2_Cl_2_	–	57	20:80	10
3	c	CH_2_Cl_2_	–	6	16:84	6
**4**	**d**	**CH_2_Cl_2_**	**–**	**80**	**8:92**	**83**
5	e	CH_2_Cl_2_	–	21	4:96	29
6	f	CH_2_Cl_2_	–	40	–	–
7	d	CH_3_OH	–	–	–	16
8	d	toluene	–	–	–	47
9	d	acetone	–	–	–	28
10	d	Et_2_O	–	–	–	41
11	d	CHCl_3_	–	–	–	2
12^e^	d	CH_2_Cl_2_	–	56	–	4
13^f^	d	CH_2_Cl_2_	–	trace	–	–
14^g^	d	CH_2_Cl_2_	–	72	–	82
15^h^	d	CH_2_Cl_2_	*p*-MeC_6_H_4_SO_3_H	–	–	25
16^h^	d	CH_2_Cl_2_	TMSCl	–	–	13
17^h^	d	CH_2_Cl_2_	TFA	–	–	11

^a^Reaction conditions: Unless noted, the reaction was carried out with **1a** (0.11 mmol), **2a** (0.1 mmol), catalyst **a**–**f** (0.01 mmol), solvent (2–3 mL), 12 h. ^b^Isolated yield. ^c^Determined by ^1^H NMR spectroscopy of the crude mixture. ^d^Determined by chiral HPLC analysis of the major diastereomer. ^e^Temperature (0 °C). ^f^Temperature (rt). ^g^Solvent (1 mL). ^h^*p*-MeC_6_H_4_SO_3_H (0.01 mmol), TMSCl (0.01 mmol), TFA (0.01 mmol) as the additive. “–” represents: not determined.

**Scheme 2:**
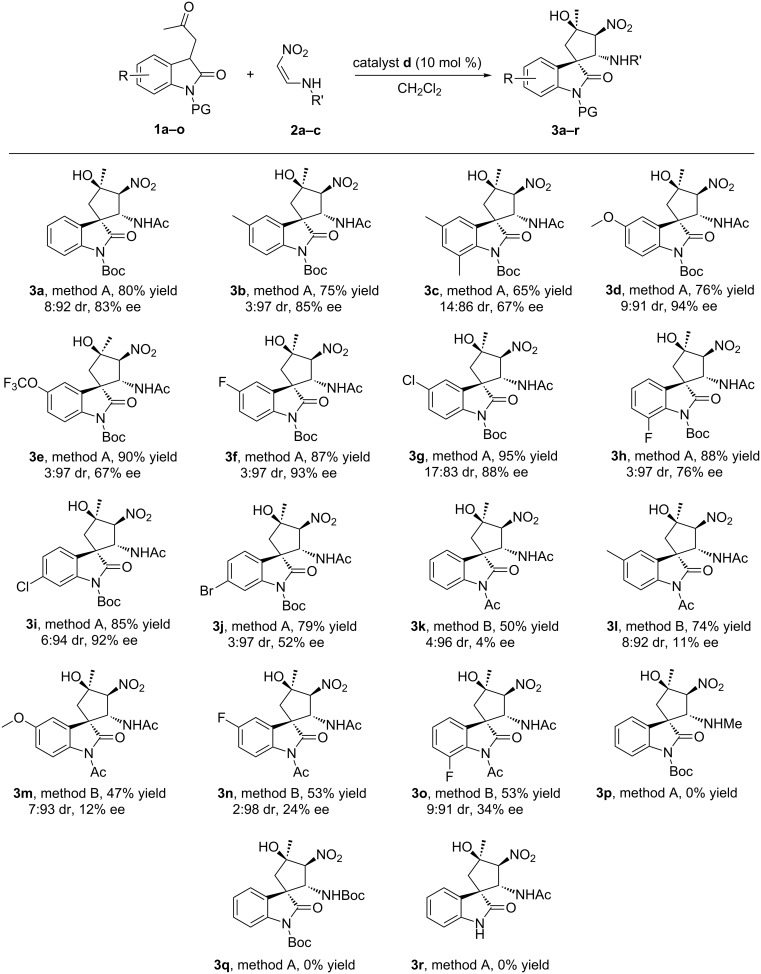
Scope of enantioselective synthesis of spirooxindoles. Reaction conditions: catalyst **d** (0.01 mmol), oxindoles **1a–o** (0.11 mmol) and nitroolefins **2a–c** (0.1 mmol) in CH_2_Cl_2_ (3 mL), method A or method B. The ee values were determined by chiral HPLC analysis of major diastereomer. The dr values were determined by ^1^H NMR analysis. Method A: with 10 mol % of **d** as a catalyst, −20 °C, 12 h. Method B: with 10 mol % of **d** as a catalyst, −10 °C, 24 h.

With the optimized reaction conditions established, we next investigated the substrate scope of 3-substituted oxindoles in this transformation with nitroolefins (Scheme 2). In general, the diverse 3-substituted oxindoles **1a–j** with electron-donating, electron-withdrawing, or halide groups could undergo a smooth reaction with nitrovinylacetamide (**2a**) in moderate yields, good diastereoselectivity, and general enantioselectivity (**3a–j**). For example, the protocol showed moderate yields (75–76%), excellent diastereoselectivity (9:91–3:97 dr) and good enantioselectivity (85–94% ee) for substrates containing 5-CH_3_ or 5-OCH_3_ groups (**3b** and **3d**). Substrates carrying 5-F, 7-F, 5-Cl and 6-Cl afforded the corresponding products **3f–i** in high yields (85–95%) with excellent diastereoselectivity (17:83–3:97 dr) and good enantioselectivity (76–93% ee). Surprisingly, introducing a bromo group into 6-position of the oxindole scaffold resulted in a low enantioselectivity (52% ee for **3j**), although at a high yield (79%) and excellent diastereoselectivity (3:97 dr). To further extend the reaction scope, we attempted to exchange the *N*-Boc group of the 3-substituted oxindoles with other protecting groups, such as Bn, CH_3_ or an acetyl group. The results demonstrated that only an acetyl protecting group proved to be well tolerated, providing for the efficient synthesis of spirocyclopentaneoxindoles **3k–o** with moderate yields (47–74%) and excellent diastereoselectivity (9:91–2:98 dr). However, replacing the *N*-Boc group with an *N*-acetyl group on spirooxindoles had a negative effect on the stereoselectivities of the target products, probably because the *N*-acetyl group decreases steric hindrance. In addition, substituents (CH_3_, Boc) at R′ and the free N-H substituted oxindole were investigated that did not give the corresponding target products (**3p**, **3q** and **3r**).

**Scheme 3:**
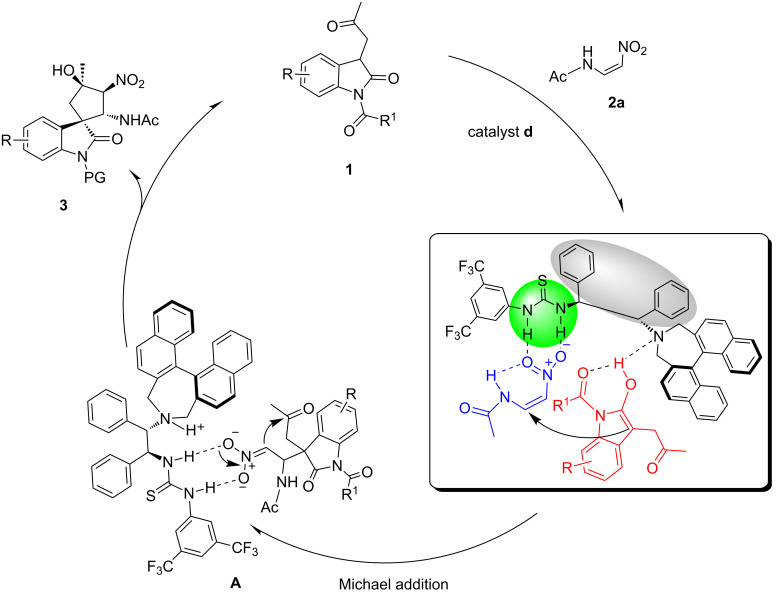
A plausible mechanism.

On the basis of the dual activation model proposed by Takemoto et al. [69], a plausible reaction mechanism was proposed in Scheme 3. The multifunctional organocatalyst **d** has a chiral scaffold including a thiourea moiety and an amino group. Both the 3-substituted oxindoles **1** and nitrovinylacetamide (**2a**) that participate in this reaction are activated simultaneously via multiple hydrogen bonds. In addition, the electrophilicity of the reacting carbon center of nitrovinylacetamide is likely enhanced by H-bonding, thereby enabling the Michael addition to construct a unique quaternary stereogenic center complex **A** which would cyclize concurrently via Henry reaction to give the product **3** and regenerates the catalyst **d**. The absolute configuration of **3g** was determined by X-ray analysis (see Supporting Information File 1, Figure S1).

## Conclusion

We have developed a highly efficient and practical strategy for a single step construction of saturated spirocyclopentaneoxindoles containing four consecutive stereocenters and a unique quaternary stereogenic center, with good yields, and excellent diastereoselectivity and enantioselectivity using thiourea-catalyzed Michael–Henry cascade reactions. We anticipate that this reaction will simplify the synthesis of complex spirooxindoles containing multiple chiral centers with potential pharmacological properties.

## Experimental

**General procedure for the synthesis of products (3a–j):** To a mixture of **1a–j** (0.11 mmol) and **2a** (0.1 mmol) in CH_2_Cl_2_ (3 mL) was added catalyst **d** (0.01 mmol). Then the mixture was stirred at −20 °C for 12 h. After completion of the reaction, the solvent was removed by evaporation. The crude product was purified by column chromatography on silica gel to afford the desired products **3a–j**.

**General procedure for the synthesis of products (3k–o):** 3-Substituted oxindoles **1k–o** (0.11 mmol) and nitrovinylacetamide (**2a**, 0.1 mmol) were dissolved in 3 mL CH_2_Cl_2_, the catalyst **d** (0.01 mmol) was added at −10 °C for 24 h. After nitrovinylacetamide (**2a**) was consumed completely, the solvent was removed under vacuum. The crude product was purified by column chromatography on silica gel to afford the desired products **3k–o**.

## Supporting Information

File 1 General information, experimental details, characterization data and copies of ^1^H and ^13^C NMR spectra, and HPLC experimental data.
