# Supplementary material for: Construction of highly enantioenriched spirocyclopentaneoxindoles containing four consecutive stereocenters via thiourea-catalyzed asymmetric Michael–Henry cascade reactions
Source: Beilstein J Org Chem. 2017 Jul 7;13:1342–9. doi: 10.3762/bjoc.13.131 (PMC5530720; doi:10.3762/bjoc.13.131)
Supplement: File 1 — General information, experimental details, characterization data and copies of 1H and 13C NMR spectra, and HPLC experimental data. [file Beilstein_J_Org_Chem-13-1342-s001.pdf]

Supporting Information

for

Construction of highly enantioenriched spirocyclopentaneoxindoles containing four consecutive stereocenters via thiourea- catalyzed asymmetric Michael–Henry cascade reactions

Yonglei Du[1], Jian Li[2], Kerong Chen[2], Chenglin Wu[2], Yu Zhou*[2] and Hong Liu*[2,3]

Address: [1]Nano Science and Technology Institute, University of Science and Technology of China, 166 Ren Ai Road, Suzhou 215123, China, [2]CAS Key Laboratory of Receptor Research, Shanghai Institute of Materia Medica, Chinese Academy of Sciences, 555, Zu Chong Zhi Road, Shanghai 201203, China and [3]University of Chinese Academy of Sciences, NO.19A Yuquan Road, Beijing 100049, China

Email: Hong Liu* - hliu@simm.ac.cn

* Corresponding author

General information, experimental details, characterization data and copies of ^{1}H and ^{13}C NMR spectra, and HPLC experimental data

Table of contents

(A) General procedures for the synthesis of substrates and products……...…....S1–S17

(B) ^{1}H NMR and ^{13}C NMR spectra for substrates and products……………...S18–S41

(C) HPLC spectra…………………………………………………………...…S42–S56

(D) X-ray data……………………………………………………..…..........S57

General information

The reagents were purchased from commercial suppliers and used without further purification. Analytical thin-layer chromatography (TLC) was performed on HSGF 254 (0.15–0.2 mm thickness), visualized by irradiation with UV light (254 nm). Column chromatography was performed using silica gel FCP 300–400. Melting points were measured with a micro melting point apparatus. ^{1}H and ^{13}C NMR spectra were recorded on a 400 MHz, 500 MHz or 600 MHz instrument (TMS as internal standard). Chemical shifts were reported in parts per million (ppm, δ) downfield from tetramethylsilane. Proton coupling patterns were described as singlet (s), doublet (d), triplet (t), multiplet (m), doublet of doublets (dd) and broad (br), triplet of doublets (td). Low- and high-resolution mass spectra (LRMS and HRMS) were measured on spectrometer (Micromass Ultra Q-TOF). Yields refer to pure compounds, unless otherwise indicated.

(A) General procedures for the synthesis of substrates and products.

Procedure for the preparation of (*Z*)-2a

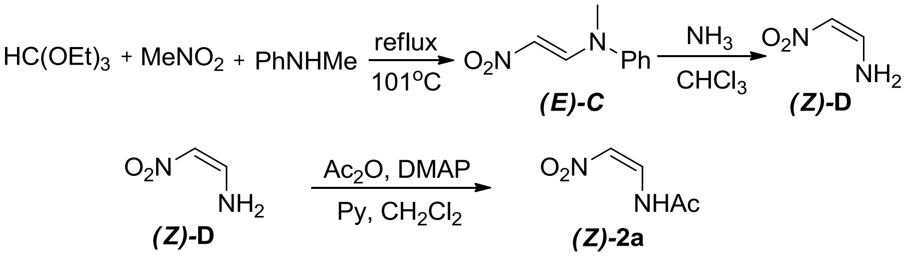

A solution of nitromethane (0.5 mol), triethyl orthoformate (0.2 mol), TsOH (0.7 g) and *N*-methylaniline (0.15 mol) was heated at 101 °C for 7 h. Then the solvent was removed by evaporation, the residue was purified by recrystallization from

toluene/petrolether to give the product (*E*)-*N*-methyl-*N*-(2-nitrovinyl) aniline **C** (18.7 g , 70%). Compound **C** was dissolved in chloroform, then resultant solution was saturated with ammonia and the mixture was kept in a refrigerator overnight. After the removal of solvent, the residue was recrystallized from chloroform to give *(Z)*-2-nitroethenamine **D** (8.3 g, 90%). To a stirred solution of (*Z*)-2-nitroethenamine **D** (4.20 g, 47.69 mmol) and DMAP (1.16 g, 9.53 mmol) in 190 mL of CH_2Cl_2, the pyridine (15.4 mL, 4.0 equiv, 191 mmol) and Ac_2O (8.90 mL, 95.4 mmol) were added at 0 °C. The reaction was allowed to continue at ambient temperature for 5 hours, then 1 mol/L HCl was added and the reaction mixture was extracted with CH_2Cl_2. The organic extracts were washed with saturated aqueous $NaHCO_3$ solution, dried over Na_2SO_4 and concentrated to give nitrovinylacetamide **2a** (5.59 g, 90%) [1] as light yellow solid. Further purification can be achieved by recrystallization from CH_2Cl_2.

***N*-(2-Nitrovinyl)acetamide (2a):** ^{1}H NMR (400 MHz, $CDCl_3$) δ 10.40 (br s, 1H), 7.59 (dd, *J* = 12.0, 6.9 Hz, 1H), 6.60 (d, *J* = 6.9 Hz, 1H), 2.28 (s, 3H). EI-MS *m/z* 131 [M+H$^+$]; HRMS (ESI) calcd for $C_4H_6N_2O_3$ [M]$^-$:129.0306, found: 129.0306.

(*Z*)-*N*-Methyl-2-nitroethenamine (**2b**): Compound **2b** was purchased from commercial suppliers and used without further purification. ^{1}H NMR (400 MHz, $CDCl_3$) δ 9.01 (s, 1H), 6.72 (dd, *J* = 13.9, 5.7 Hz, 1H), 6.46 (d, *J* = 5.7 Hz, 1H), 3.17 (d, *J* = 5.2 Hz, 3H). EI-MS *m/z* 103 [M+H$^+$]; HRMS (ESI) calcd for $C_3H_6N_2O_2$ [M+H$^+$]:103.0502, found: 103.0499.

(*Z*)-*tert*-Butyl-(2-nitrovinyl)carbamate (**2c**): Compound **2c** was purchased from commercial suppliers and used without further purification. ^{1}H NMR (400 MHz,

$CDCl_3$) δ 9.71 (s, 1H), 7.36 (dd, *J* = 12.5, 6.8 Hz, 1H), 6.56 (d, *J* = 6.8 Hz, 1H), 1.54 (s, 9H). EI-MS *m/z* 189 [M+H$^+$]; HRMS (ESI) calcd for $C_7H_{12}N_2O_4$ [M]$^-$:187.0724, found: 187.0721.

Procedure for the preparation of 1a–j

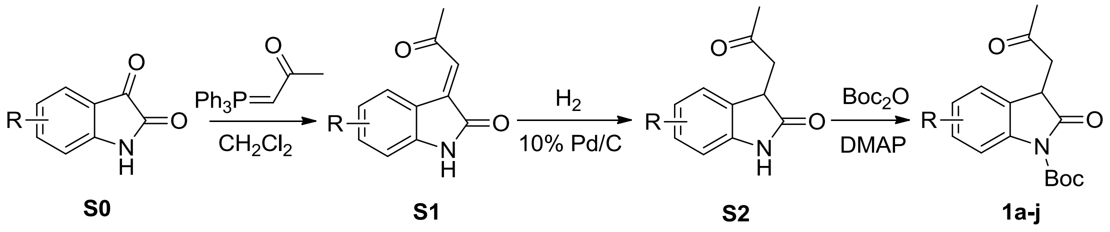

To a stirred solution of **S0** (10 mmol) in CH_2Cl_2 (180 mL) was added slowly triphenylphosphoranylidene-2-propanon (11 mmol, 1.1 equiv) in CH_2Cl_2 (20 mL) at room temperature, and the resulting mixture was stirred at the same temperature for 8 hours. Then the mixture was concentrated under reduced pressure, and the crude product was purified by column chromatography on silica gel to afford the desired product **S1** (yield, 90%). Compound **S1** (2 mmol) was dissolved in 50 mL methanol and was allowed to react in the presence of Pd/C (0.2 mmol, 0.1 equiv; 10% loading in 50% water) under a hydrogen atmosphere for 1 hour. After the reaction was completed, the reaction liquid was filtrated through celite and evaporated to give a residue, which was purified via flash chromatography on silica gel using petroleum/EtOAc (4:1 to 2:1) to afford the desired product **S2** as a light yellow oil (yield, 80%). The di-*tert*-butyl-dicarbonate (1.1 mmol, 1.1equiv) in 5 mL of CH_2Cl_2 was added slowly to a solution of 3-substituted indoline **S2** (1.0 mmol) in CH_2Cl_2 (30 mL) in the presence of DMAP (0.1 mmol, 0.1 equiv) at −40 °C. The reaction was allowed to continue overnight at −40 °C. After the reaction was completed, the

aqueous sat. NH_4Cl was added and the reaction mixture was extracted with CH_2Cl_2. The crude product was purified by flash chromatography on silica gel using petroleum/EtOAc (6:1 to 4:1) to afford desired product **1a−j** as colourless liquid, which solidified over time [2].

1-*tert*-Butyloxycarbonyl-3-(2-oxopropyl)indolin-2-one (1a): This compound is known [2], colourless liquid, 59% yield; ^{1}H NMR (500 MHz, $CDCl_3$) δ 7.81 (d, J = 8.2 Hz, 1H), 7.31 – 7.27 (m, 1H), 7.15 (d, *J* = 7.4 Hz, 1H), 7.09 (t, *J* = 7.5 Hz, 1H), 3.95 (dd, *J* = 7.7, 3.3 Hz, 1H), 3.26 (dd, *J* = 18.5, 3.5 Hz, 1H), 2.97 (dd, *J* = 18.5, 7.9 Hz, 1H), 2.20 (s, 3H), 1.64 (s, 9H). EI-MS *m/z* 312 [M+Na$^+$]; HRMS (ESI) calcd for $C_{16}H_{19}NO_4Na$ [M+Na$^+$]: 312.1212; observed: 312.1203.

1-*tert*-Butyloxycarbonyl-5-methyl-3-(2-oxopropyl)indolin-2-one (1b): This compound is known [2], colourless liquid, 58% yield; ^{1}H NMR (600 MHz, $CDCl_3$) δ 7.68 (d, *J* = 8.3 Hz, 1H), 7.08 (d, *J* = 8.3 Hz, 1H), 6.97 (s, 1H), 3.94 (dd, *J* = 8.0, 3.4 Hz, 1H), 3.25 (dd, *J* = 18.5, 3.4 Hz, 1H), 2.94 (dd, *J* = 18.4, 8.1 Hz, 1H), 2.30 (s, 3H), 2.22 (s, 3H), 1.63 (s, 9H). EI-MS *m/z* 304 [M+H$^+$]; HRMS (ESI) calcd for $C_{17}H_{21}NO_4$ [M+H$^+$]: 304.1543; observed: 304.1533.

1-*tert*-Butyloxycarbonyl-5,7-dimethyl-3-(2-oxopropyl)indolin-2-one (1c): colourless liquid, 50% yield; ^{1}H NMR (500 MHz, $CDCl_3$) δ 6.88 (s, 1H), 6.80 (s, 1H), 3.94 (dd, *J* = 7.9, 3.5 Hz, 1H), 3.21 (dd, *J* = 18.4, 3.6 Hz, 1H), 2.92 (dd, *J* = 18.4, 8.0 Hz, 1H), 2.26 (s, 3H), 2.20 (s, 3H), 2.19 (s, 3H), 1.61 (s, 9H). EI-MS *m/z* 340 [M+Na$^+$]; HRMS (ESI) calcd for $C_{18}H_{23}NO_4Na$ [M+Na$^+$]: 340.1512; observed: 340.1517.

1-*tert*-Butyloxycarbonyl-5-methoxy-3-(2-oxopropyl)indolin-2-one (1d): This compound is known [2], colourless liquid, 62% yield; ^{1}H NMR (500 MHz, $CDCl_3$) δ 7.72 (d, *J* = 8.9 Hz, 1H), 6.79 (dd, *J* = 8.9, 2.5 Hz, 1H), 6.74 (d, *J* = 1.7 Hz, 1H), 3.93 (dd, *J* = 7.9, 3.4 Hz, 1H), 3.76 (s, 3H), 3.25 (dd, *J* = 18.5, 3.5k Hz, 1H), 2.95 (dd, *J* = 18.5, 8.0 Hz, 1H), 2.20 (s, 3H), 1.62 (s, 9H). EI-MS *m/z* 342 [$M+Na^+$]; HRMS (ESI) calcd for $C_{17}H_{21}NO_5Na$ [$M+Na^+$]: 342.1317; observed: 342.1320.

1-*tert*-Butyloxycarbonyl-5-trifluoromethoxy-3-(2-oxopropyl)indolin-2-one (1e): colourless liquid, 52% yield; ^{1}H NMR (500 MHz, DMSO) δ 7.81 (d, *J* = 8.9 Hz, 1H), 7.36 (s, 1H), 7.31 (dd, *J* = 8.9, 0.8 Hz, 1H), 3.97 (t, *J* = 4.7 Hz, 1H), 3.53 (dd, *J* = 18.9, 4.1 Hz, 1H), 3.24 (dd, *J* = 19.0, 5.5 Hz, 1H), 2.10 (s, 3H), 1.58 (s, 9H). EI-MS *m/z* 372 [$M]^-$; HRMS (ESI) calcd for $C_{17}H_{18}F_3NO_5$ [$M]^-$: 372.1064; observed: 372.1059.

1-*tert*-Butyloxycarbonyl-5-fluoro-3-(2-oxopropyl)indolin-2-one (1f): colourless liquid, 56% yield; ^{1}H NMR (400 MHz, $CDCl_3$) δ 7.80 (dd, *J* = 8.9, 4.6 Hz, 1H), 6.98 (td, *J* = 9.0, 2.6 Hz, 1H), 6.94 – 6.89 (m, 1H), 3.93 (dd, *J* = 8.1, 2.7 Hz, 1H), 3.28 (dd, *J* = 18.6, 3.3 Hz, 1H), 2.98 (dd, *J* = 18.6, 8.0 Hz, 1H), 2.22 (s, 3H), 1.63 (s, 9H). EI-MS *m/z* 330 [$M+Na^+$]; HRMS (ESI) calcd for $C_{16}H_{18}FNO_4Na$ [$M+Na^+$]: 330.1118; observed: 330.1124.

1-*tert*-Butyloxycarbonyl-5-chloro-3-(2-oxopropyl)indolin-2-one (1g): colourless liquid, 65% yield; ^{1}H NMR (400 MHz, $CDCl_3$) δ 7.76 (d, *J* = 8.7 Hz, 1H), 7.26 – 7.22 (m, 1H), 7.14 (d, *J* = 0.7 Hz, 1H), 3.89 (dd, *J* = 7.6, 3.0 Hz, 1H), 3.26 (dd, *J* = 18.7, 3.3 Hz, 1H), 2.99 (dd, *J* = 18.7, 7.8 Hz, 1H), 2.20 (s, 3H), 1.62 (s, 9H).

EI-MS *m/z* 346 [M+Na$^+$]; HRMS (ESI) calcd for $C_{16}H_{18}ClNO_4Na$ [M+Na$^+$]: 346.0822; observed: 346.0828.

1-*tert*-Butyloxycarbonyl-7-fluoro-3-(2-oxopropyl)indolin-2-one (1h): colourless liquid, 75% yield; ^{1}H NMR (400 MHz, $CDCl_3$) δ 7.89 (s, 1H), 7.08 (d, *J* = 0.9 Hz, 2H), 3.89 (dd, *J* = 7.8, 3.4 Hz, 1H), 3.26 (dd, *J* = 18.5, 3.4 Hz, 1H), 2.98 (dd, *J* = 18.5, 7.9 Hz, 1H), 2.20 (s, 3H), 1.64 (s, 9H). EI-MS *m/z* 330 [M+Na$^+$]; HRMS (ESI) calcd for $C_{16}H_{18}FNO_4Na$ [M+Na$^+$]: 330.1118; observed: 330.1122.

1-*tert*-Butyloxycarbonyl-6-chloro-3-(2-oxopropyl)indolin-2-one (1i): This compound is known [2], colourless liquid, 50% yield; ^{1}H NMR (400 MHz, $CDCl_3$) δ 7.11 – 7.01 (m, 2H), 7.00 – 6.92 (m, 1H), 3.99 (dd, *J* = 8.0, 3.3 Hz, 1H), 3.29 (dd, *J* = 18.6, 3.3 Hz, 1H), 3.00 (dd, *J* = 18.6, 8.0 Hz, 1H), 2.21 (s, 3H), 1.61 (s, 9H). EI-MS m/z 346 [M+Na$^+$]; HRMS (ESI) calcd for $C_{16}H_{18}ClNO_4Na$ [M+Na$^+$]: 346.0822; observed: 346.0822.

1-*tert*-Butyloxycarbonyl-6-bromo-3-(2-oxopropyl)indolin-2-one (1j): colourless liquid, 51% yield; ^{1}H NMR (400 MHz, DMSO) δ 7.89 (d, *J* = 1.5 Hz, 1H), 7.33 (dd, *J* = 7.9, 1.6 Hz, 1H), 7.22 (d, *J* = 8.0 Hz, 1H), 3.86 (t, *J* = 4.6 Hz, 1H), 3.45 (dd, *J* = 18.8, 3.8 Hz, 1H), 3.20 (dd, *J* = 19.0, 5.7 Hz, 1H), 2.08 (s, 3H), 1.56 (s, 9H). EI-MS (*m/z*): 392, 390 [M+Na$^+$, (Br^{81}), (Br^{79})]. HRMS (ESI) calcd for $C_{16}H_{18}BrNO_4$ [M]$^-$: 366.0346; observed: 366.0345.

Procedure for the preparation of 1k–o

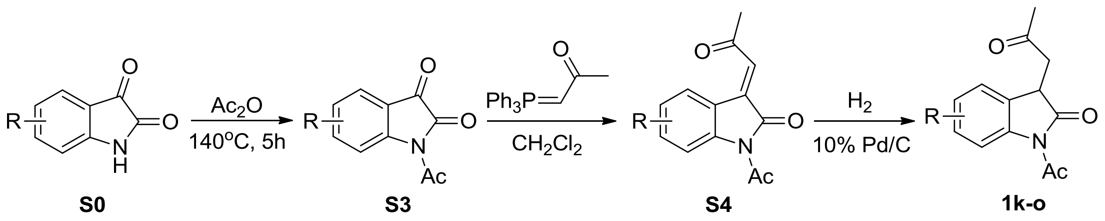

Isatin-derivative **S0** (10 mmol) was dissolved in Ac_2O (50 mL) at 140 °C for 5 h. After the reaction was completed, the reaction was cooled, the precipitated solid was filtered off, washed with petroleum and recrystallized from CH_2Cl_2 to give purer product **S3**. Compound **S3** to **1k–o**, the same as Compound **S1** to **1a–j**.

1-Acetyl-3-(2-oxopropyl)indolin-2-one (1k): white solid, 50% yield; ^1H NMR (400 MHz, DMSO) δ 8.10 – 8.04 (m, 1H), 7.32 – 7.24 (m, 2H), 7.19 – 7.10 (m, 1H), 3.97 (t, *J* = 4.8 Hz, 1H), 3.46 (dd, *J* = 18.8, 4.0 Hz, 1H), 3.22 (dd, *J* = 18.8, 5.8 Hz, 1H), 2.55 (s, 3H), 2.10 (s, 3H). EI-MS *m/z* 230 $[M]^-$; HRMS (ESI) calcd for $C_{13}H_{13}NO_3$ $[M]^-$: 230.0823; observed: 230.0824.

1-Acetyl-5-methyl-3-(2-oxopropyl)indolin-2-one (1l): white solid, 54% yield; ^1H NMR (400 MHz, DMSO) δ 7.96 (d, *J* = 8.1 Hz, 1H), 7.12 – 7.05 (m, 2H), 3.94 (t, *J* = 4.8 Hz, 1H), 3.44 (dd, *J* = 18.9, 3.9 Hz, 1H), 3.21 (dd, *J* = 18.8, 5.8 Hz, 1H), 2.54 (s, 3H), 2.27 (s, 3H), 2.11 (s, 3H). EI-MS *m/z* 244 $[M]^-$; HRMS (ESI) calcd for $C_{14}H_{15}NO_3$ $[M]^-$: 244.0979; observed: 244.0978.

1-Acetyl-5-methoxy-3-(2-oxopropyl)indolin-2-one (1m): white solid, 57% yield; ^1H NMR (400 MHz, DMSO) δ 7.99 (d, *J* = 8.9 Hz, 1H), 6.93 (dd, *J* = 2.6, 1.1 Hz, 1H), 6.83 (dd, *J* = 8.9, 2.3 Hz, 1H), 3.95 (t, *J* = 4.8 Hz, 1H), 3.73 (s, 3H), 3.48 (dd, *J* = 18.8, 4.0 Hz, 1H), 3.23 (dd, *J* = 18.8, 5.6 Hz, 1H), 2.53 (s, 3H), 2.10 (s, 3H). EI-MS *m/z*

260 $[M]^-$; HRMS (ESI) calcd for $C_{14}H_{15}NO_4$ $[M]^-$: 260.0928; observed: 260.0926.

1-Acetyl-5-fluoro-3-(2-oxopropyl)indolin-2-one (1n): white solid, 53% yield; ^{1}H NMR (400 MHz, DMSO) δ 8.08 (dd, *J* = 8.9, 4.9 Hz, 1H), 7.22 (dd, *J* = 8.4, 1.7 Hz, 1H), 7.12 (td, *J* = 9.3, 2.8 Hz, 1H), 4.00 (t, *J* = 4.3 Hz, 1H), 3.52 (dd, *J* = 19.0, 3.9 Hz, 1H), 3.27 (dd, *J* = 19.0, 5.5 Hz, 1H), 2.55 (s, 3H), 2.10 (s, 3H). EI-MS *m/z* 248 $[M]^-$; HRMS (ESI) calcd for $C_{13}H_{12}FNO_3$ $[M]^-$: 248.0728; observed: 248.0725.

1-Acetyl-7-fluoro-3-(2-oxopropyl)indolin-2-one (1o): white solid, 58% yield; ^{1}H NMR (400 MHz, DMSO) δ 7.23 – 7.17 (m, 1H), 7.17 – 7.10 (m, 2H), 4.05 (t, *J* = 4.5 Hz, 1H), 3.51 (dd, *J* = 19.0, 3.8 Hz, 1H), 3.26 (dd, *J* = 19.0, 5.5 Hz, 1H), 2.55 (s, 3H), 2.07 (s, 3H). EI-MS *m/z* 248 $[M]^-$; HRMS (ESI) calcd for $C_{13}H_{12}FNO_3$ $[M]^-$: 248.0728; observed: 248.0725.

General procedure for the synthesis of products (3a–j).

To a mixture of **1a–j** (0.11 mmol) and **2** (0.1 mmol) in CH_2Cl_2 (3 mL) was added catalyst **d** (0.01 mmol). Then the mixture was reacted at −20 ^oC for 12h. After the completion of the reaction, the solvent was removed by evaporation. The crude product was purified by column chromatography on silica gel to afford the desired product **3a-j**.

(1*S*,2*R*,3*S*,4*R*)-*tert*-Butyl-2-acetamido-4-hydroxy-4-methyl-3-nitro-2'-oxospiro-[cyclopentane-1,3'-indoline]-1'-carboxylate (3a): white solid, 80% yield; (dr =8:92, 83% ee for the major diastereomer); M.P.: 155.0−156.0°C, ^{1}H NMR (500 MHz, $CDCl_3$) δ 7.73 (d, *J* = 7.1 Hz, 1H), 7.67 (d, *J* = 8.0 Hz, 1H), 7.30 (t, *J* = 7.3 Hz, 1H), 7.24 (d, *J* = 7.3 Hz, 1H), 6.03 (d, *J* = 10.1 Hz, 1H), 5.90 (t, *J* = 10.7 Hz, 1H), 5.11 (d,

J = 11.4 Hz, 1H), 3.37 (s, 1H), 2.49 (d, J = 14.5 Hz, 1H), 2.28 (d, J = 14.5 Hz, 1H), 1.82 (s, 3H), 1.65 (s, 9H), 1.61 (s, 3H). ^{13}C NMR (126 MHz, $CDCl_3$) δ 178.71, 169.63, 148.32, 138.85, 130.15, 128.92, 125.83, 124.48, 114.52, 92.52, 85.25, 75.89, 58.74, 54.53, 48.47, 27.97, 25.47, 22.87. HRMS (ESI) calcd for $C_{20}H_{25}N_3O_7Na$ [M+Na$^+$]: 442.1590; observed: 442.1594. HPLC conditions: chiralpak OD-H, hexane/iso-PrOH = 85:15, flow rate = 0.8 mL/min, λ= 254 nm, retention time: 11.46 min (major) and 15.95 min (minor).

(1*S*,2*R*,3*S*,4*R*)-*tert*-Butyl-2-acetamido-4-hydroxy-4,5'-dimethyl-3-nitro-2'-oxospiro[cyclopentane-1,3'-indoline]-1'-carboxylate (3b): white solid, 75% yield; (dr =3:97, 85% ee for the major diastereomer); M.P.: 156.9−159.6°C, ^{1}H NMR (400 MHz, $CDCl_3$) δ 7.53 (d, J = 8.5 Hz, 2H), 7.11 – 7.07 (m, 1H), 5.99 – 5.81 (m, 2H), 5.08 (d, J = 11.0 Hz, 1H), 2.46 (d, J = 14.4 Hz, 1H), 2.35 (s, 3H), 2.26 (d, J = 14.5 Hz, 1H), 1.83 (s, 3H), 1.64 (s, 9H), 1.60 (s, 3H). ^{13}C NMR (151 MHz, CDCl3) δ 178.98, 169.59, 148.48, 136.60, 135.74, 130.16, 129.58, 125.03, 114.43, 92.68, 85.15, 75.94, 58.86, 54.61, 48.51, 28.09, 25.54, 23.01, 21.18. HRMS (ESI) calcd for $C_{21}H_{27}N_3O_7Na$ [M+Na$^+$]: 456.1747; observed: 456.1744. HPLC conditions: chiralpak OZ-3-H, hexane/iso-PrOH = 85:15, flow rate = 0.8 mL/min, λ= 220 nm, retention time: 34.21 min (major) and 13.76 min (minor).

(1*S*,2*R*,3*S*,4*R*)-*tert*-Butyl-2-acetamido-4-hydroxy-4,5',7'-trimethyl-3-nitro-2'-oxospiro[cyclopentane-1,3'-indoline]-1'-carboxylate (3c): white solid, 65% yield; (dr =14:86, 67% ee for the major diastereomer); M.P.: 153.5−155.3°C, ^{1}H NMR (400 MHz, DMSO) δ 8.13 (d, J = 8.3 Hz, 1H), 7.24 (s, 1H), 6.90 (s, 1H), 5.98 (s, 1H), 5.59

– 5.49 (m, 1H), 5.12 (d, *J* = 11.6 Hz, 1H), 2.42 (d, *J* = 14.6 Hz, 1H), 2.28 (s, 3H), 2.12 (d, *J* = 7.6 Hz, 1H), 2.06 (s, 3H), 1.57 (s, 3H), 1.53 (s, 9H), 1.50 (s, 3H). ^{13}C NMR (126 MHz, DMSO) δ 178.33, 169.21, 148.95, 135.84, 134.13, 132.60, 131.93, 122.54, 122.32, 92.68, 84.75, 75.66, 59.15, 55.24, 48.75, 27.80, 26.58, 22.65, 21.17, 19.19. HRMS (ESI) calcd for $C_{22}H_{29}N_3O_7Na$ [M+Na$^+$]: 470.1903; observed: 470.1890. HPLC conditions: chiralpak OZ-3-H, hexane/iso-PrOH = 80:20, flow rate = 0.8 mL/min, λ= 220 nm, retention time: 23.02 min (major) and 10.73 min (minor).

(1*S*,2*R*,3*S*,4*R*)-*tert*-Butyl-2-acetamido-4-hydroxy-5'-methoxy-4-methyl-3-nitro-2'-oxospiro[cyclopentane-1,3'-indoline]-1'-carboxylate (3d): white solid, 76% yield; (dr =9:91, 94% ee for the major diastereomer); M.P.: 177.4−178.6°C, ^{1}H NMR (400 MHz, DMSO) δ 8.23 (d, *J* = 8.1 Hz, 1H), 7.53 (d, *J* = 8.9 Hz, 1H), 7.26 (d, *J* = 2.6 Hz, 1H), 6.86 (dd, *J* = 8.9, 2.6 Hz, 1H), 6.02 (s, 1H), 5.53 (dd, *J* = 11.3, 8.3 Hz, 1H), 5.18 (d, *J* = 11.4 Hz, 1H), 3.78 (s, 3H), 2.50 (d, *J* = 14.7 Hz, 1H), 2.15 (d, *J* = 14.7 Hz, 1H), 1.61 (s, 3H), 1.56 (s, 9H), 1.53 (s, 3H). ^{13}C NMR (126 MHz, DMSO) δ 177.28, 169.41, 157.11, 148.76, 132.99, 132.75, 115.25, 113.20, 110.98, 92.90, 84.09, 75.68, 59.26, 55.86, 55.24, 49.35, 28.11, 26.65, 22.61. HRMS (ESI) calcd for $C_{21}H_{27}N_3O_8Na$ [M+Na$^+$]: 472.1696; observed: 472.1705. HPLC conditions: chiralpak OZ-3-H, hexane/iso-PrOH = 85:15, flow rate = 0.8 mL/min, λ= 254 nm, retention time: 37.32 min (major) and 13.56 min (minor).

(1*S*,2*R*,3*S*,4*R*)-*tert*-Butyl-2-acetamido-4-hydroxy-5'-trifluoromethoxy-4-methyl-3-nitro-2'-oxospiro[cyclopentane-1,3'-indoline]-1'-carboxylate (3e): white solid, 90% yield; (dr =3:97, 67% ee for the major diastereomer); M.P.: 169.6−170.7°C, ^{1}H NMR

(500 MHz, DMSO) δ 8.29 (d, *J* = 7.9 Hz, 1H), 7.64 (d, *J* = 8.9 Hz, 1H), 7.60 (d, *J* = 1.9 Hz, 1H), 7.26 (dd, *J* = 8.9, 1.8 Hz, 1H), 6.04 (s, 1H), 5.42 (dd, *J* = 11.3, 7.9 Hz, 1H), 5.10 (d, *J* = 11.4 Hz, 1H), 2.48 (d, *J* = 14.8 Hz, 1H), 2.10 (d, *J* = 14.8 Hz, 1H), 1.56 (s, 3H), 1.51 (s, 9H), 1.48 (s, 3H). ^{13}C NMR (126 MHz, DMSO) δ 176.57, 169.67, 148.55, 145.64, 138.49, 134.27, 121.42, 120.6 (q, J =255.8 Hz) 117.75, 115.77, 92.77, 84.71, 75.90, 59.72, 55.26, 49.06, 28.06, 26.30, 22.46. HRMS (ESI) calcd for $C_{21}H_{24}F_3N_3O_8Na$ $[M]^-$: 502.1443; observed: 502.1445. HPLC conditions: chiralpak OZ-3-H, hexane/iso-PrOH = 90:10, flow rate = 1.0 mL/min, λ= 230 nm, retention time: 12.46 min (major) and 6.78 min (minor).

(1*S*,2*R*,3*S*,4*R*)-*tert*-Butyl-2-acetamido-4-hydroxy-5'-fluoro-4-methyl-3-nitro-2'-oxospiro[cyclopentane-1,3'-indoline]-1'-carboxylate (3f): white solid, 87 % yield; (dr =3:97, 93% ee for the major diastereomer); M.P.: 140.0−141.5°C, ^{1}H NMR (400 MHz, $CDCl_3$) δ 7.66 (dd, *J* = 9.0, 4.4 Hz, 1H), 7.51 (dd, *J* = 7.8, 2.5 Hz, 1H), 7.00 (td, *J* = 8.8, 2.5 Hz, 1H), 5.98 (d, *J* = 10.1 Hz, 1H), 5.89 – 5.80 (m, 1H), 5.03 (d, *J* = 11.3 Hz, 1H), 2.48 (d, *J* = 14.5 Hz, 1H), 2.26 (d, *J* = 14.5 Hz, 1H), 1.85 (s, 3H), 1.64 (s, 9H), 1.61 (s, 3H). ^{13}C NMR (151 MHz, $CDCl_3$) δ 178.22, 169.56, 160.75 (d, J_{C-F} =246.1Hz), 148.32, 134.88 (d, J_{C-F} =1.5Hz), 132.53 (d, J_{C-F} =9.1Hz), 116.09 (d, J_{C-F} =7.6Hz), 115.73 (d, J_{C-F} =22.7Hz), 112.28 (d, J_{C-F} =25.7Hz), 92.57, 85.49, 76.08, 59.11, 54.90, 48.17, 28.07, 25.17, 22.97. HRMS (ESI) calcd for $C_{20}H_{24}FN_3O_7Na$ $[M+Na^+]$: 460.1496; observed: 460.1483. HPLC conditions: chiralpak OZ-3-H, hexane/iso-PrOH = 90:10, flow rate = 1.0 mL/min, λ= 220 nm, retention time: 25.16min (major) and 13.86 min (minor).

(1*S*,2*R*,3*S*,4*R*)-*tert*-Butyl-2-acetamido-4-hydroxy-5'-chloro-4-methyl-3-nitro-2'-oxospiro[cyclopentane-1,3'-indoline]-1'-carboxylate (3g): white solid, 95% yield; (dr =17:83, 88% ee for the major diastereomer); M.P.: 161.2−163.6°C, ^{1}H NMR (400 MHz, $CDCl_3$) δ 7.75 (s, 1H), 7.64 (d, *J* = 8.8 Hz, 1H), 7.34 – 7.27 (m, 1H), 5.94 (d, *J* = 9.8 Hz, 1H), 5.84 (t, *J* = 10.4 Hz, 1H), 5.00 (d, *J* = 11.2 Hz, 1H), 2.47 (d, *J* = 14.5 Hz, 1H), 2.28 (s, 1H), 2.21 (d, *J* = 20.4 Hz, 1H), 1.85 (s, 3H), 1.64 (s, 9H), 1.60 (s, 3H). ^{13}C NMR (151 MHz, $CDCl_3$) δ 177.98, 169.64, 148.20, 137.47, 132.39, 131.42, 129.14, 124.95, 115.96, 92.58, 85.67, 76.09, 59.11, 54.75, 48.22, 28.06, 25.17, 22.97. HRMS (ESI) calcd for $C_{20}H_{24}ClN_3O_7Na$ [M+Na$^+$]: 476.1200; observed: 476.1199. HPLC conditions: chiralpak OZ-3-H, hexane/iso-PrOH = 90:10, flow rate = 0.8 mL/min, λ= 254 nm, retention time: 35.91 min (major) and 18.41 min (minor).

(1*S*,2*R*,3*S*,4*R*)-*tert*-Butyl-2-acetamido-4-hydroxy-7'-fluoro-4-methyl-3-nitro-2'-oxospiro[cyclopentane-1,3'-indoline]-1'-carboxylate (3h): white solid, 88% yield; (dr =3:97, 76% ee for the major diastereomer); M.P.: 122.7−125.2°C, ^{1}H NMR (400 MHz, $CDCl_3$) δ 7.54 (d, *J* = 7.5 Hz, 1H), 7.22 (td, *J* = 7.9, 4.4 Hz, 1H), 7.11 – 7.00 (m, 1H), 6.01 (d, *J* = 9.9 Hz, 1H), 5.88 (t, *J* = 10.8 Hz, 1H), 5.06 (d, *J* = 11.4 Hz, 1H), 2.65 (s, 1H), 2.50 (d, *J* = 14.5 Hz, 1H), 2.30 (d, *J* = 14.6 Hz, 1H), 1.85 (s, 3H), 1.62 (s, 3H), 1.60 (s, 9H). ^{13}C NMR (151 MHz, $CDCl_3$) δ 177.76, 169.65, 147.99 (d, J_{C-F} =250.7Hz), 146.91, 133.50, 126.91 (d, J_{C-F} =7.6Hz), 125.90 (d, J_{C-F} =9.1Hz), 120.42 (d, J_{C-F} =3.0Hz), 117.35 (d, J_{C-F} =19.6Hz), 92.26, 85.88, 76.07, 59.10, 55.18, 47.80, 27.65, 25.46, 22.92. HRMS (ESI) calcd for $C_{20}H_{24}FN_3O_7Na$ [M+Na$^+$]: 460.1496; observed: 460.1487. HPLC conditions: chiralpak OZ-3-H, hexane/iso-PrOH = 85:15,

flow rate = 0.8 mL/min, λ= 220 nm, retention time: 35.28 min (major) and 25.77min (minor).

(1*S*,2*R*,3*S*,4*R*)-*tert*-Butyl-2-acetamido-4-hydroxy-6'-chloro-4-methyl-3-nitro-2'-oxospiro[cyclopentane-1,3'-indoline]-1'-carboxylate (3i): white solid, 85% yield; (dr =6:94, 92% ee for the major diastereomer); M.P.: 138.5−141.4°C, ^{1}H NMR (400 MHz, $CDCl_3$) δ 7.75 (s, 1H), 7.66 (d, *J* = 8.1 Hz, 1H), 7.23 (d, *J* = 8.1 Hz, 1H), 5.98 – 5.76 (m, 2H), 5.03 (d, *J* = 11.1 Hz, 1H), 3.31 (s, 1H), 2.46 (d, *J* = 14.6 Hz, 1H), 2.23 (d, *J* = 14.3 Hz, 1H), 1.85 (s, 3H), 1.65 (s, 9H), 1.60 (s, 3H). ^{13}C NMR (151 MHz, $CDCl_3$) δ 178.22, 169.52, 148.13, 139.82, 134.81, 128.79, 126.04, 125.55, 115.57, 92.47, 85.84, 75.98, 58.87, 54.43, 48.32, 28.04, 25.36, 23.02. HRMS (ESI) calcd for $C_{20}H_{24}ClN_3O_7Na$ [M+Na$^+$]: 476.1200; observed: 476.1210. HPLC conditions: chiralpak OZ-3-H, hexane/iso-PrOH = 90:10, flow rate = 0.8 mL/min, λ= 254 nm, retention time: 51.61 min (major) and 34.22 min (minor).

(1*S*,2*R*,3*S*,4*R*)-*tert*-Butyl-2-acetamido-4-hydroxy-6'-bromo-4-methyl-3-nitro-2'-oxospiro[cyclopentane-1,3'-indoline]-1'-carboxylate (3j): white solid, 79% yield; (dr = 3:97, 52% ee for the major diastereomer); M.P.: 144.5−147.6°C, ^{1}H NMR (500 MHz, DMSO) δ 8.31 (d, *J* = 7.9 Hz, 1H), 7.77 (d, *J* = 1.8 Hz, 1H), 7.59 (d, *J* = 8.1 Hz, 1H), 7.46 (dd, *J* = 8.1, 1.8 Hz, 1H), 6.04 (s, 1H), 5.45 (dd, *J* = 11.3, 7.9 Hz, 1H), 5.12 (d, *J* = 11.3 Hz, 1H), 2.49 (d, *J* =14.3 Hz, 1H), 2.13 (d, *J* = 14.7 Hz, 1H), 1.61 (s, 3H), 1.56 (s, 9H), 1.52 (s, 3H). ^{13}C NMR (126 MHz, DMSO) δ 176.06, 169.11, 148.09, 140.29, 131.17, 127.45, 125.99, 120.44, 116.70, 92.27, 84.29, 75.39, 59.12, 54.44, 48.58, 27.54, 25.82, 22.02. HRMS (ESI) calcd for $C_{20}H_{24}BrN_3O_7$ [M]$^-$: 496.0725;

observed: 496.0740. HPLC conditions: chiralpak OZ-3-H, hexane/iso-PrOH = 90:10, flow rate = 1.0 mL/min, λ= 230 nm, retention time: 43.95 min (major) and 28.58 min (minor).

General procedure for the synthesis of products (3k–o).

3-Substituted oxindoles **1k–o** (0.11mmol) and nitrovinylacetamide **2a** (0.1mmol) were dissolved in 3 mL CH_2Cl_2, the catalyst **d** (0.01 mmol) was added at −10 °C for 24 h. After nitrovinylacetamide **2a** was consumed completely, the solvent was removed under vacuum. The crude product was purified by column chromatography on silica gel to afford the desired product **3k-o**.

***N*-((1*S*,2*R*,3*S*,4*R*)-1'-acetyl-3-hydroxy-3-methyl-4-nitro-2'-oxospiro[cyclopentane -1,3'-indolin]-5-yl)acetamide (3k):** white solid, 50% yield; (dr =4:96, 4% ee for the major diastereomer); M.P.: 192.0−193.3°C, ^{1}H NMR (500 MHz, DMSO) δ 8.18 (d, *J* = 8.4 Hz, 1H), 7.97 – 7.94 (m, 1H), 7.68 – 7.63 (m, 1H), 7.32 – 7.24 (m, 2H), 6.03 (s, 1H), 5.58 (dd, *J* = 11.5, 8.4 Hz, 1H), 5.18 (d, *J* = 11.5 Hz, 1H), 2.58 (s, 3H), 2.54 (d, *J* = 14.7 Hz, 1H), 2.19 (d, *J* = 14.7 Hz, 1H), 1.59 (s, 3H), 1.55 (s, 3H). ^{13}C NMR (126 MHz, DMSO) δ 179.81, 170.70, 169.47, 140.09, 131.76, 128.59, 125.82, 124.50, 115.43, 92.90, 75.62, 59.27, 55.06, 49.31, 26.71, 26.68, 22.59. HRMS (ESI) calcd for $C_{17}H_{19}N_3O_6$ $[M]^-$: 360.1201; observed: 360.1202. HPLC conditions: chiralpak AD-H, hexane/iso-PrOH = 85:15, flow rate = 1.0 mL/min, λ= 230 nm, retention time: 32.74 min (major) and 7.56 min (minor).

***N*-((1*S*,2*R*,3*S*,4*R*)-1'-Acetyl-3-hydroxy-3,5'-dimethyl-4-nitro-2'-oxospiro[cyclo-pentane-1,3'-indolin]-5-yl)acetamide (3l):** white solid, 74% yield; (dr =8:92, 11% ee

for the major diastereomer); M.P.: 173.1−175.5°C,^{1}H NMR (500 MHz, DMSO) δ 8.17 (d, *J* = 8.4 Hz, 1H), 7.85 (d, *J* = 8.3 Hz, 1H), 7.48 (s, 1H), 7.11 (d, *J* = 7.3 Hz, 1H), 6.02 (s, 1H), 5.57 (dd, *J* = 11.4, 8.4 Hz, 1H), 5.18 (d, *J* = 11.5 Hz, 1H), 2.58 (s, 3H), 2.55 (d, *J* = 14.7 Hz, 1H), 2.35 (s, 3H), 2.19 (d, *J* = 14.7 Hz, 1H), 1.61 (s, 3H), 1.55 (s, 3H). ^{13}C NMR (126 MHz, DMSO) δ 179.43, 170.00, 168.96, 137.34, 134.32, 131.24, 128.52, 124.31, 114.77, 92.43, 75.11, 58.75, 54.55, 48.79, 26.19, 26.10, 22.11, 20.88. HRMS (ESI) calcd for $C_{18}H_{21}N_3O_6$ $[M]^-$: 374.1358; observed: 374.1352. HPLC conditions: chiralpak AD-H, hexane/iso-PrOH = 85:15, flow rate = 1.0 mL/min, λ= 254 nm, retention time: 24.06 min (major) and 5.97 min (minor).

***N*-((1*S*,2*R*,3*S*,4*R*)-1'-acetyl-3-hydroxy-5'-methoxy-3-methyl-4-nitro-2'-oxospiro[cyclopentane-1,3'-indolin]-5-yl)acetamide (3m):** white solid, 47% yield; (dr =7:93, 12% ee for the major diastereomer); M.P.: 150.0−156.4°C, ^{1}H NMR (500 MHz, DMSO) δ 8.18 (d, *J* = 8.4 Hz, 1H), 7.88 (d, *J* = 8.9 Hz, 1H), 7.26 (d, *J* = 2.7 Hz, 1H), 6.84 (dd, *J* = 8.9, 2.8 Hz, 1H), 6.04 (s, 1H), 5.56 (dd, *J* = 11.5, 8.4 Hz, 1H), 5.17 (d, *J* = 11.5 Hz, 1H), 3.77 (s, 3H), 2.55 (s, 3H), 2.52 (d, *J* = 14.8 Hz, 1H), 2.19 (d, *J* = 14.8 Hz, 1H), 1.60 (s, 3H), 1.54 (s, 3H). ^{13}C NMR (126 MHz, DMSO) δ 179.74, 170.27, 169.50, 157.44, 133.50, 133.20, 116.43, 113.16, 110.73, 92.92, 75.61, 59.28, 55.86, 55.31, 49.20, 26.71, 26.51, 22.59. HRMS (ESI) calcd for $C_{18}H_{21}N_3O_7$ $[M]^-$: 390.1307; observed: 390.1315. HPLC conditions: chiralpak AD-H, hexane/iso-PrOH = 85:15, flow rate = 1.0 mL/min, λ= 254 nm, retention time: major diastereomer: 39.47 min (major) and 9.19 min (minor).

***N*-((1*S*,2*R*,3*S*,4*R*)-1'-acetyl-5'-fluoro-3-hydroxy-3-methyl-4-nitro-2'-oxospiro[cyclopentane-1,3'-indolin]-5-yl)acetamide (3n):** white solid, 53% yield; (dr =2:98, 24% ee for the major diastereomer); M.P.: 194.6−195.7°C, ^{1}H NMR (600 MHz, DMSO) δ 8.28 (d, *J* = 8.1 Hz, 1H), 7.97 (dd, *J* = 9.0, 4.8 Hz, 1H), 7.51 (dd, *J* = 8.4, 2.8 Hz, 1H), 7.12 (td, *J* = 9.1, 2.8 Hz, 1H), 6.07 (s, 1H), 5.52 (dd, *J* = 11.4, 8.2 Hz, 1H), 5.15 (d, *J* = 11.5 Hz, 1H), 2.55 (s, 3H), 2.53 (d, *J* = 14.8 Hz, 1H), 2.19 (d, *J* = 14.8 Hz, 1H), 1.60 (s, 3H), 1.53 (s, 3H). ^{13}C NMR (151 MHz, DMSO) δ 179.26, 170.53, 169.70, 160.10 (d, J_{C-F} = 241.6 Hz) 136.43, 134.42 (d, J_{C-F} = 9.1 Hz), 117.06 (d, J_{C-F} = 7.6 Hz), 115.07 (d, J_{C-F} = 22.7 Hz), 111.85 (d, J_{C-F} = 24.2 Hz), 92.77, 75.76, 59.50, 55.35, 48.96, 26.57, 26.52, 22.53. HRMS (ESI) calcd for $C_{17}H_{18}FN_3O_6$ $[M]^-$: 378.1107; observed: 378.1105. HPLC conditions: chiralpak AD-H, hexane/iso-PrOH = 85:15, flow rate = 1.0 mL/min, λ= 254 nm, retention time: 13.29 min (major) and 5.87 min (minor).

***N*-((1*S*,2*R*,3*S*,4*R*)-1'-acetyl-7'-fluoro-3-hydroxy-3-methyl-4-nitro-2'-oxospiro[cyclopentane-1,3'-indolin]-5-yl)acetamide (3o):** white solid, 53% yield; (dr =9:91, 34% ee for the major diastereomer); M.P.: 173.5−174.8°C, ^{1}H NMR (600 MHz, DMSO) δ 8.25 (d, *J* = 8.2 Hz, 1H), 7.47 (dd, *J* = 7.5, 0.9 Hz, 1H), 7.30 (td, *J* = 8.1, 4.4 Hz, 1H), 7.15 (dd, *J* = 10.7, 8.4 Hz, 1H), 6.05 (s, 1H), 5.51 (dd, *J* = 11.5, 8.2 Hz, 1H), 5.10 (d, *J* = 11.5 Hz, 1H), 2.57 (s, 3H), 2.54 (d, *J* = 14.9 Hz, 1H), 2.19 (d, *J* = 14.9 Hz, 1H), 1.57 (s, 3H), 1.52 (s, 3H). ^{13}C NMR (151 MHz, DMSO) δ 179.06, 169.54, 168.06, 148.83 (d, J_{C-F} = 252.2 Hz), 135.69 (d, J_{C-F} = 3.0 Hz), 127.48 (d, J_{C-F} = 7.6 Hz), 125.92 (d, J_{C-F} = 10.6 Hz), 120.41 (d, J_{C-F} = 3.0 Hz), 116.94 (d, J_{C-F} = 21.1 Hz), 92.58,

75.76, 59.82, 56.17, 48.64, 26.51, 25.97, 22.53. HRMS (ESI) calcd for $C_{17}H_{18}FN_3O_6$ $[M]^-$: 378.1107; observed: 378.1108. HPLC conditions: chiralpak AD-H, hexane/iso-PrOH = 85:15, flow rate = 1.0 mL/min, λ= 230 nm, retention time: 27.04 min (major) and 7.58 min (minor).

References

[1] Zhu, S.; Yu, S.; Wang, Y.; Ma, D. *Angew. Chem. Int. Ed.* **2010**, *49*, 4656-4660.

[2] Albertshofer, K.; Tan, B.; Barbas, C. F., III *Org. Lett.* **2012**, *14*, 1834-1837.

(B) ^{1}H and ^{13}C NMR Spectra for Substrates and Products

(Z)-*N*-(2-nitrovinyl)acetamide (**2a**)

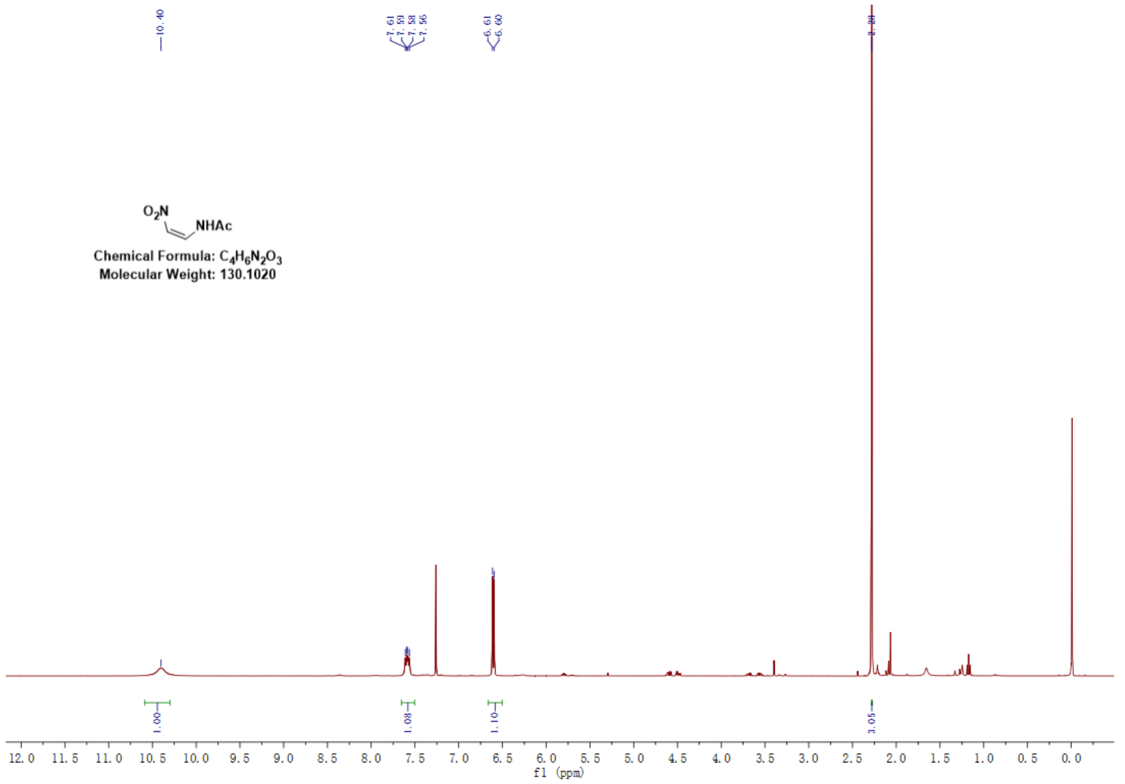

(*Z*)-*N*-methyl-2-nitroethenamine (**2b**)

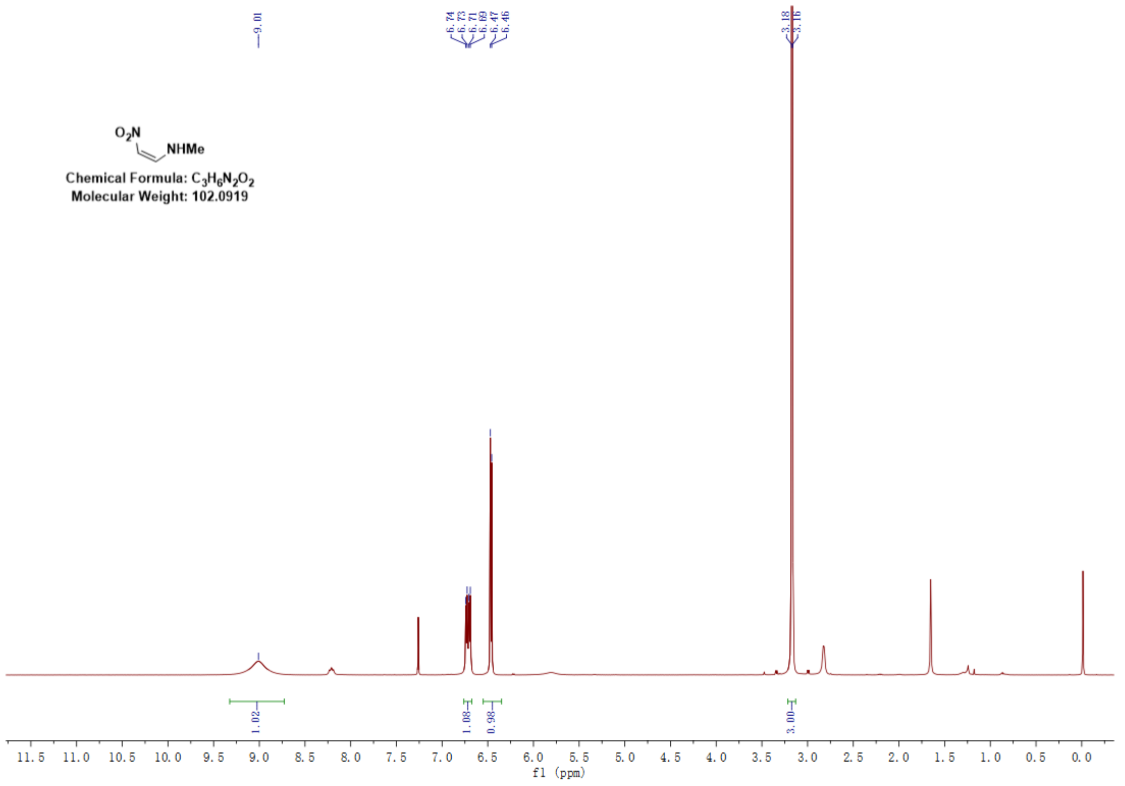

(*Z*)-*tert*-butyl-(2-nitrovinyl)carbamate (**2c**)

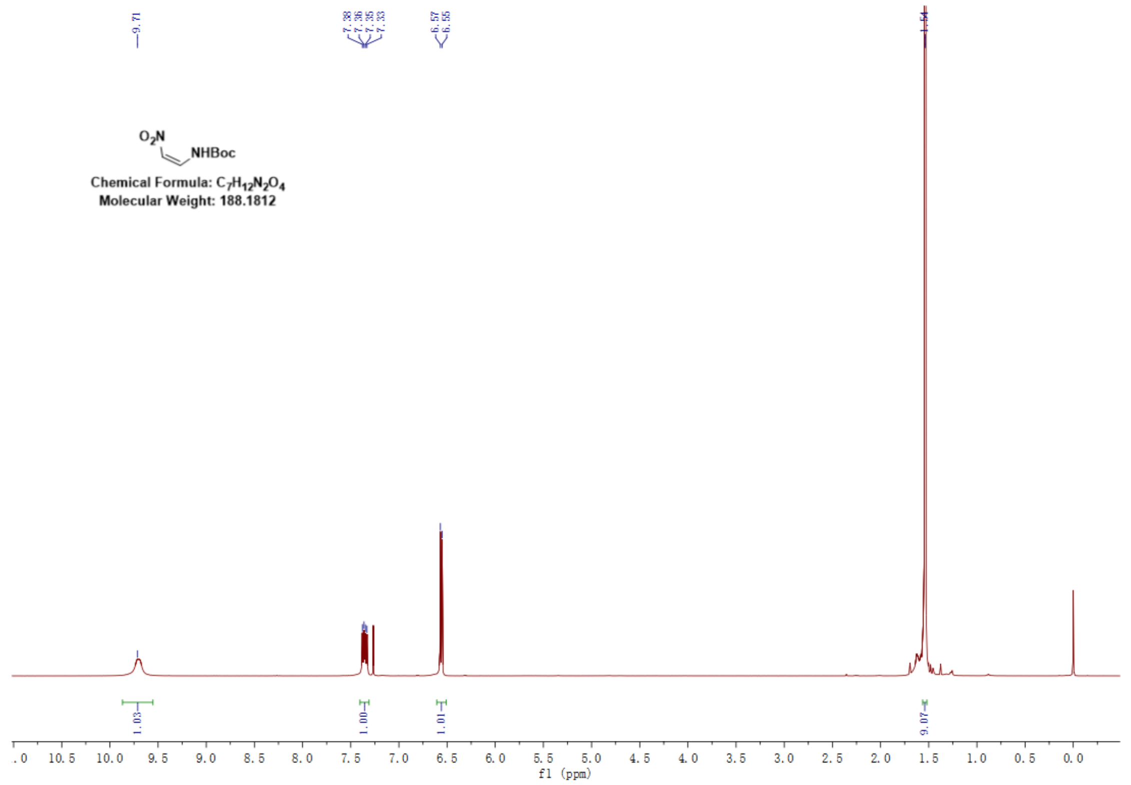

1-*tert*-Butyloxycarbonyl-3-(2-oxopropyl)indolin-2-one (**1a**)

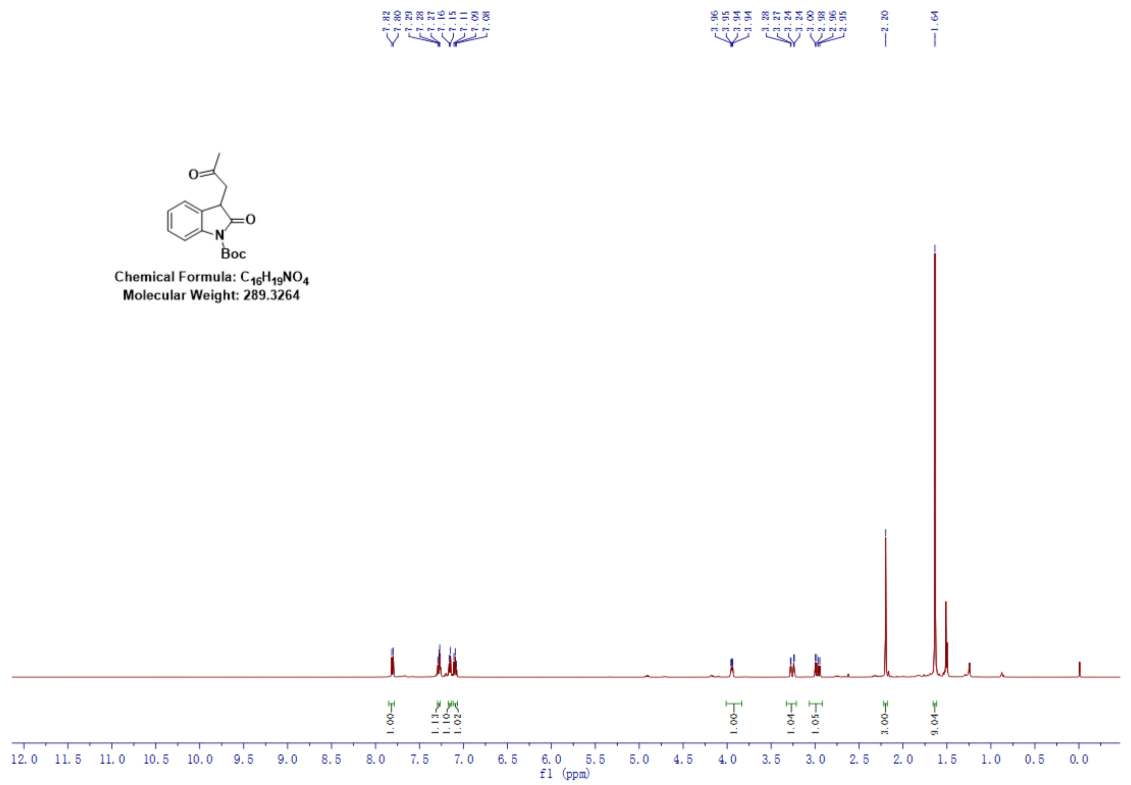

1-*tert*-Butyloxycarbonyl-5-methyl-3-(2-oxopropyl)indolin-2-one **(1b)**

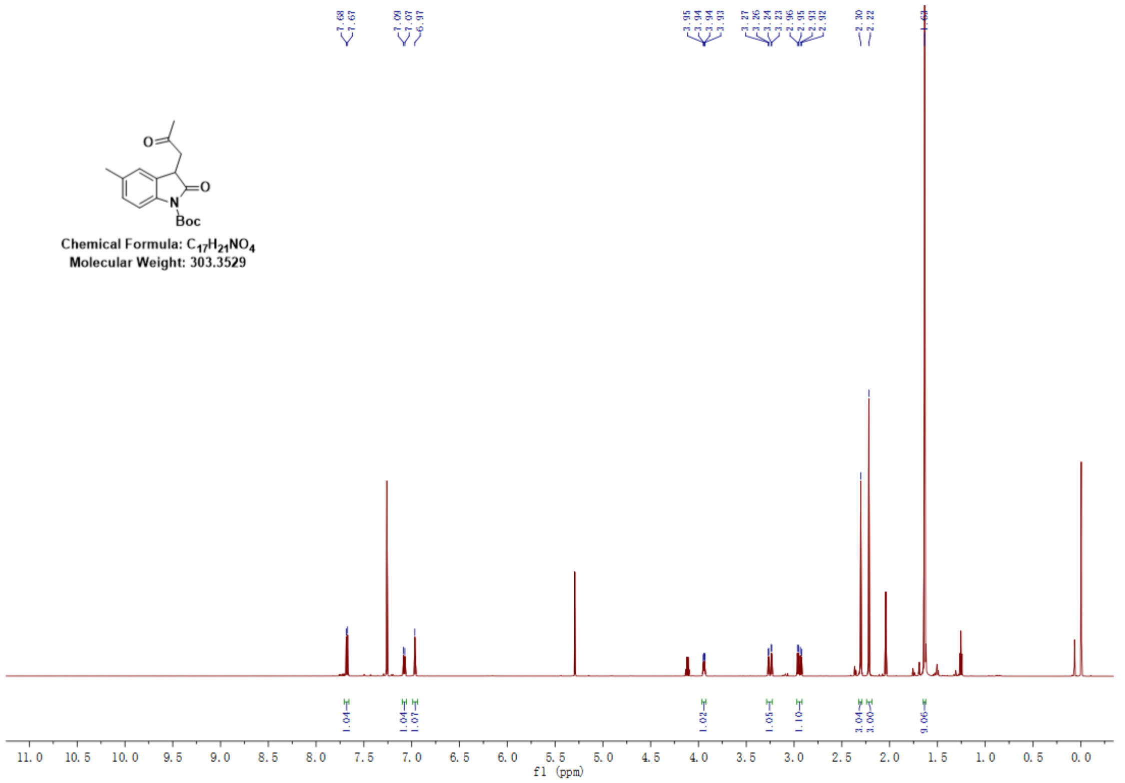

1-*tert*-Butyloxycarbonyl-5,7-dimethyl-3-(2-oxopropyl)indolin-2-one **(1c)**

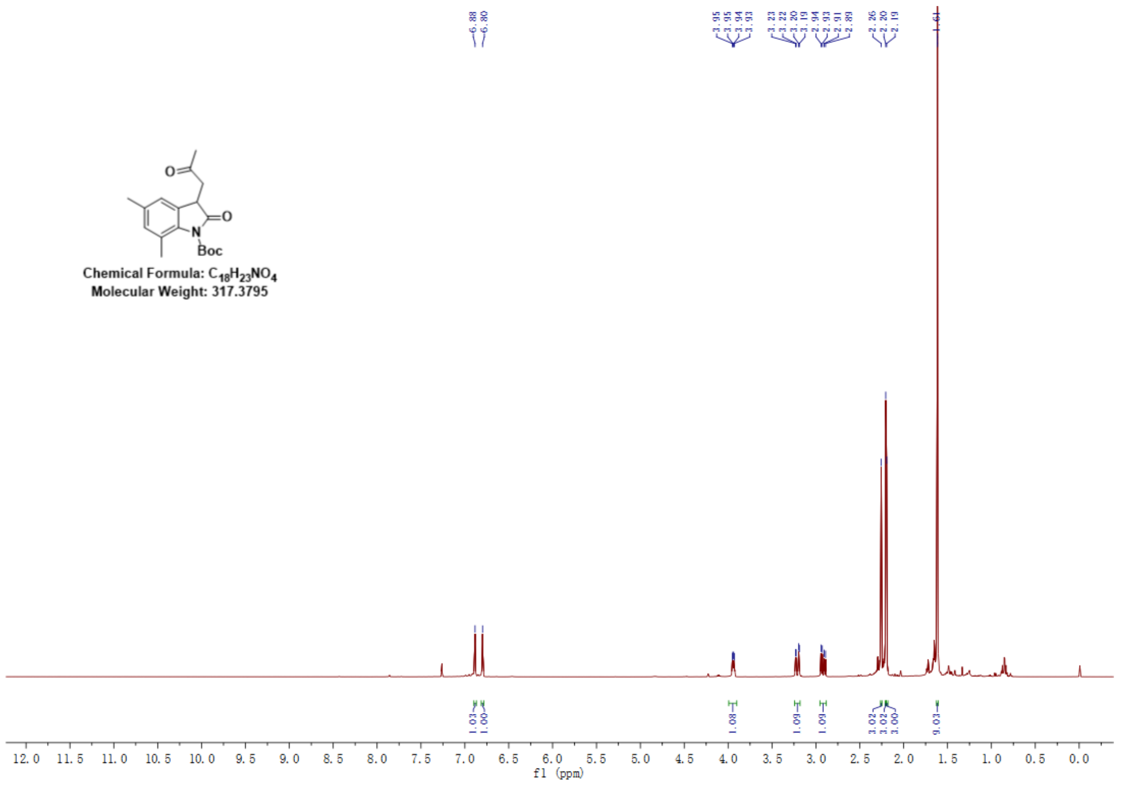

1-*tert*-Butyloxycarbonyl-5-methoxy-3-(2-oxopropyl)indolin-2-one **(1d)**

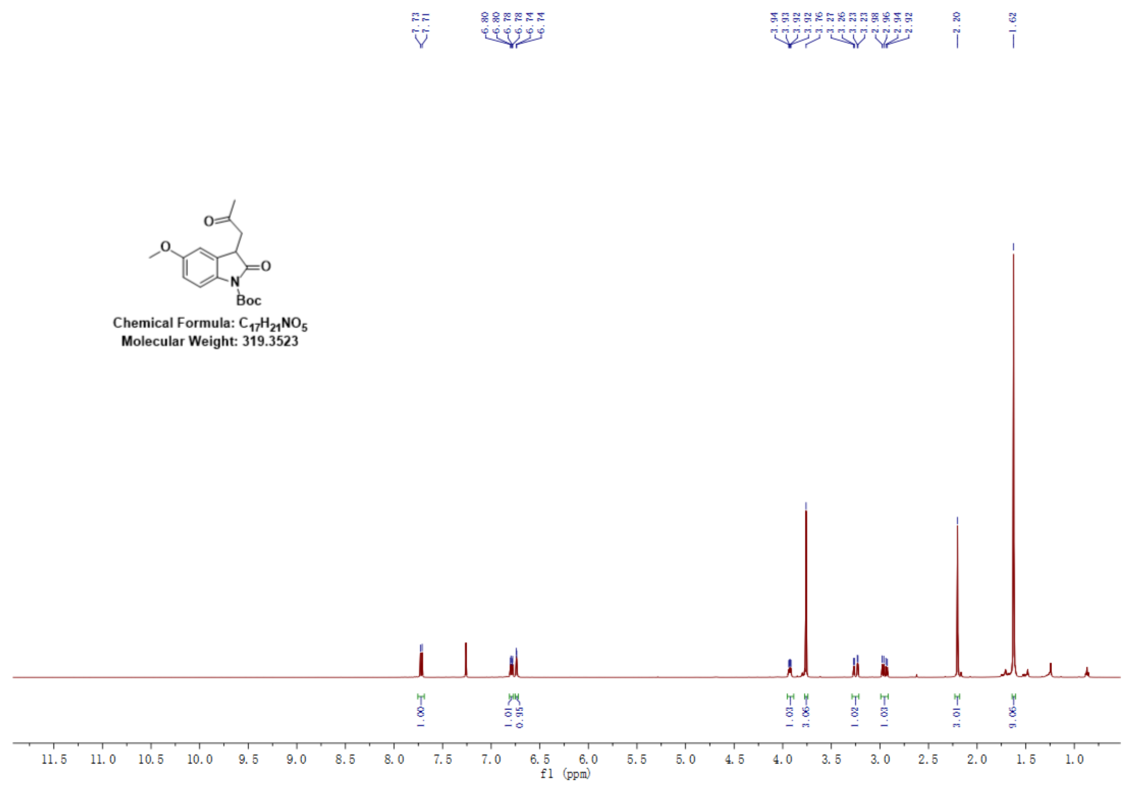

1-*tert*-Butyloxycarbonyl-5-trifluoromethoxy-3-(2-oxopropyl)indolin-2-one **(1e)**

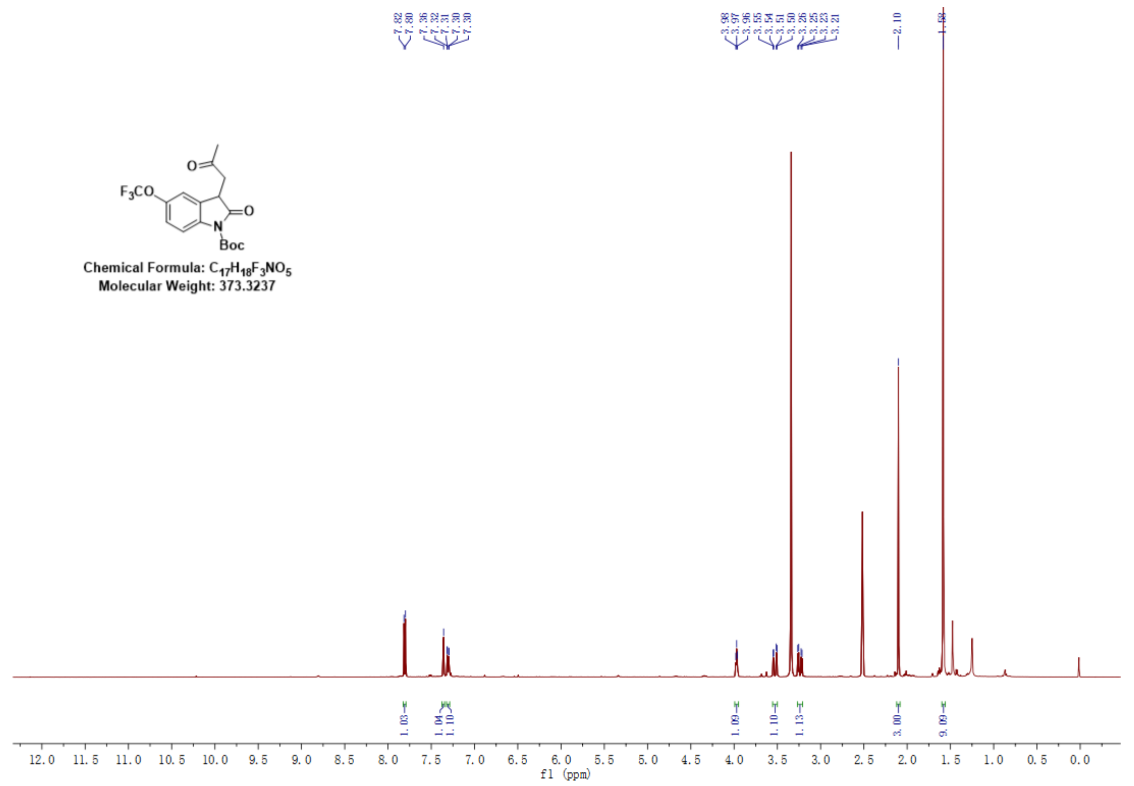

1-*tert*-Butyloxycarbonyl-5-fluoro-3-(2-oxopropyl)indolin-2-one **(1f)**

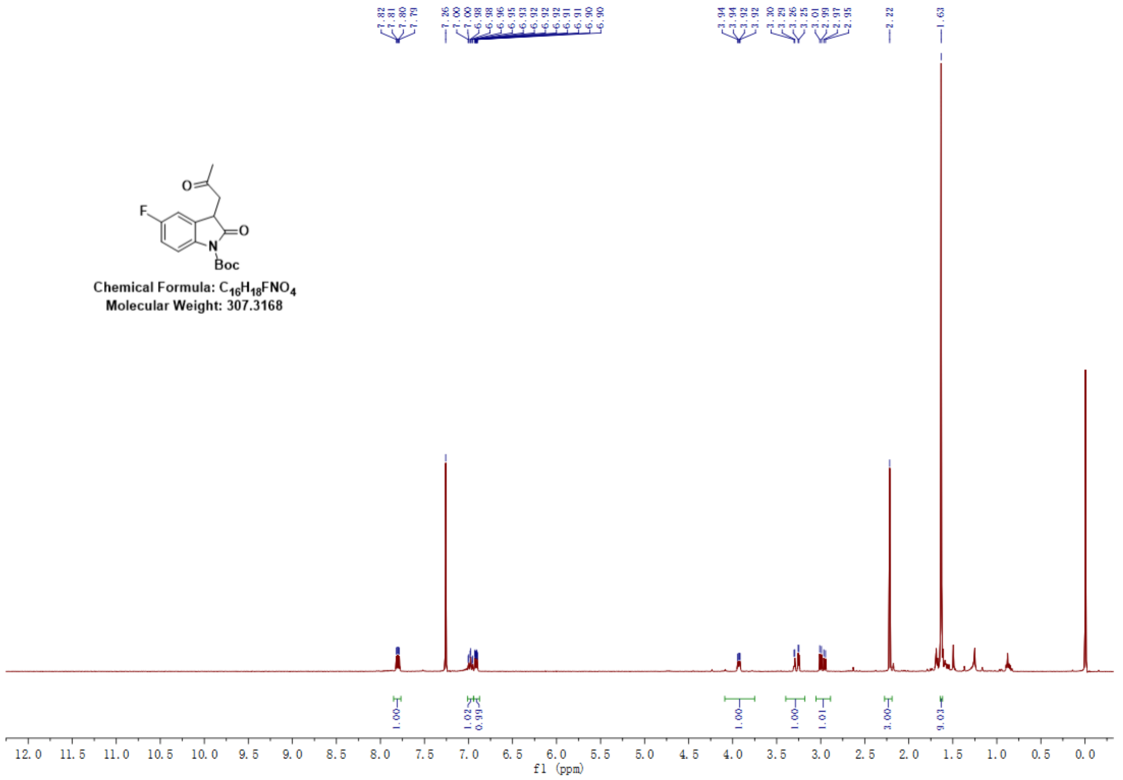

1-*tert*-Butyloxycarbonyl-5-chloro-3-(2-oxopropyl)indolin-2-one **(1g)**

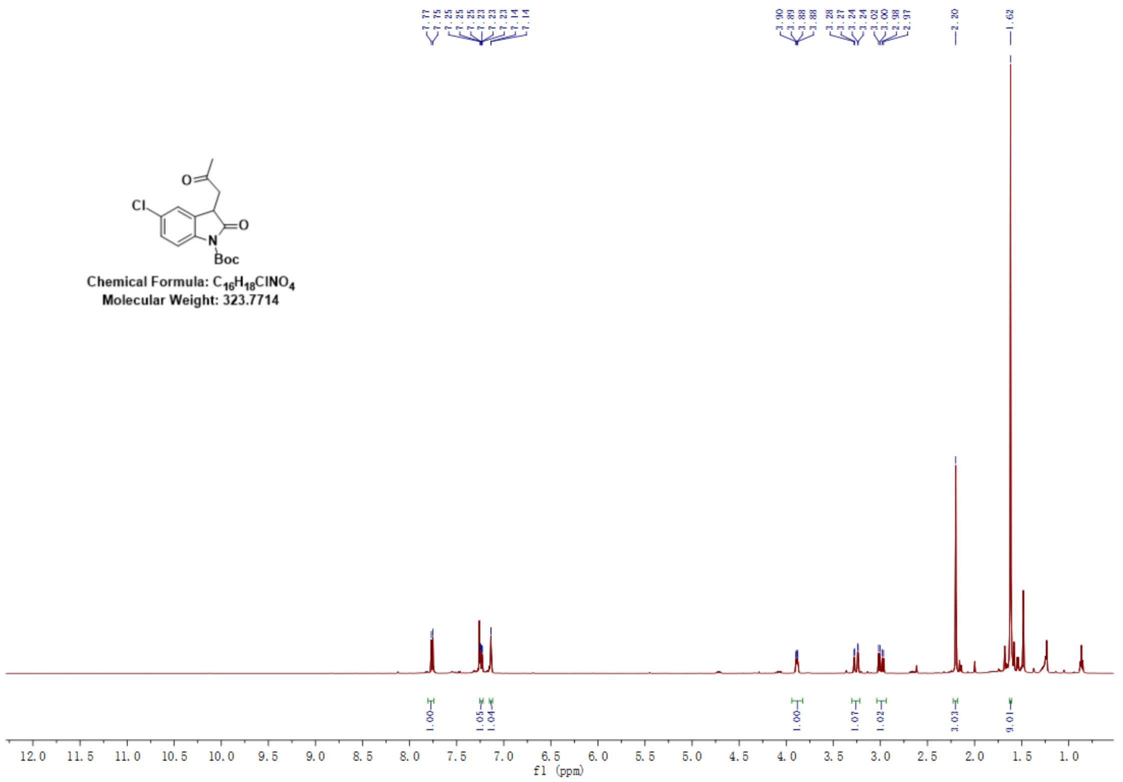

1-*tert*-Butyloxycarbonyl-7-fluoro-3-(2-oxopropyl)indolin-2-one **(1h)**

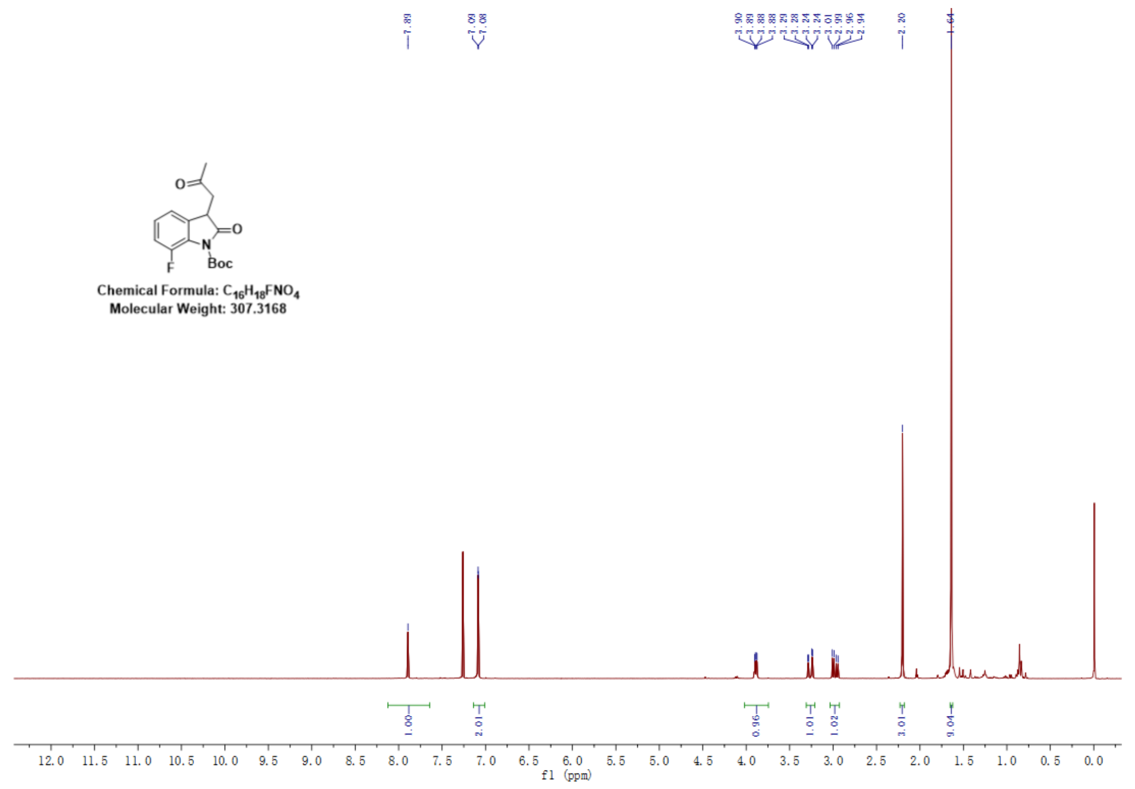

1-*tert*-Butyloxycarbonyl-6-chloro-3-(2-oxopropyl)indolin-2-one **(1i)**

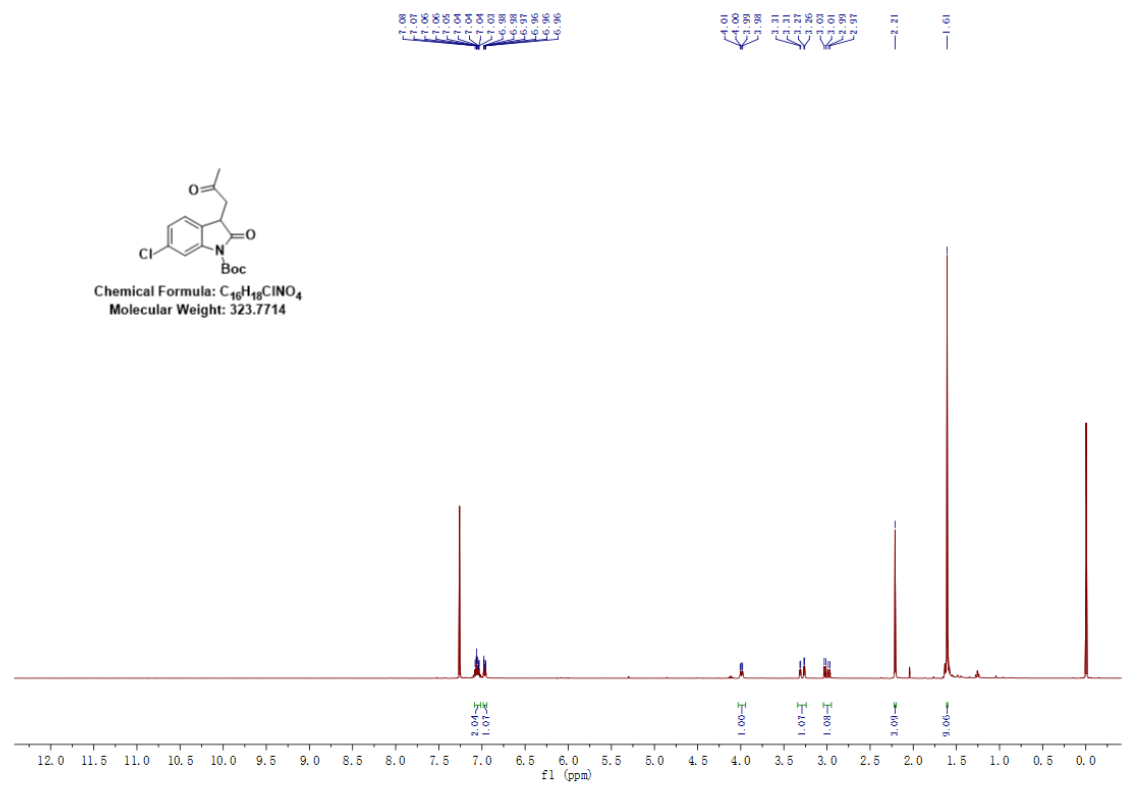

1-*tert*-Butyloxycarbonyl-6-bromo-3-(2-oxopropyl)indolin-2-one **(1j)**

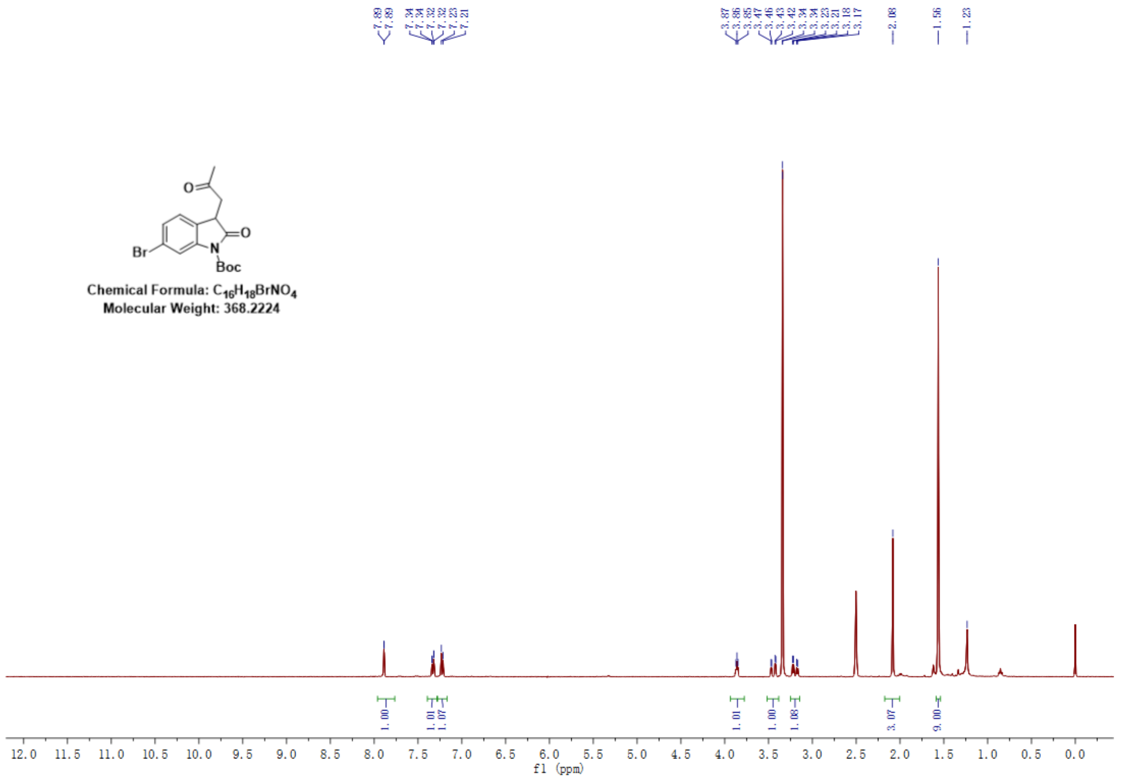

1-Acetyl-3-(2-oxopropyl)indolin-2-one (**1k**)

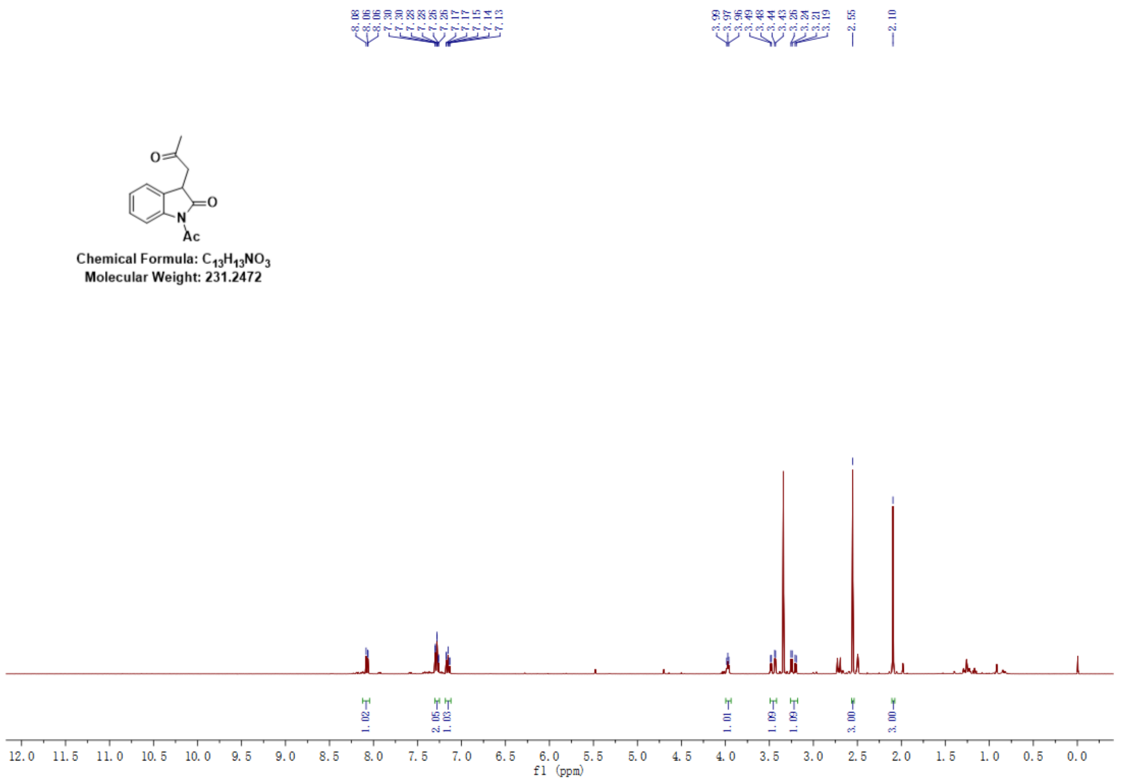

1-Acetyl-5-methyl-3-(2-oxopropyl)indolin-2-one **(1l)**

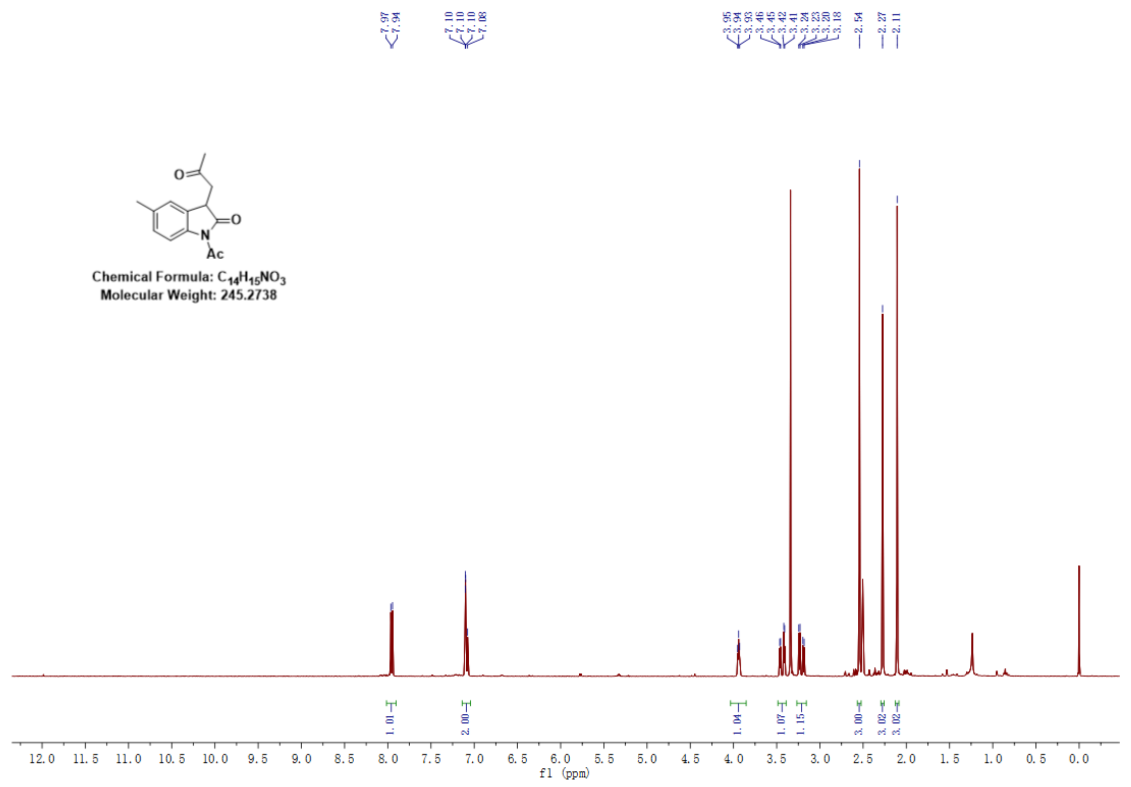

1-Acetyl-5-methoxy-3-(2-oxopropyl)indolin-2-one **(1m)**

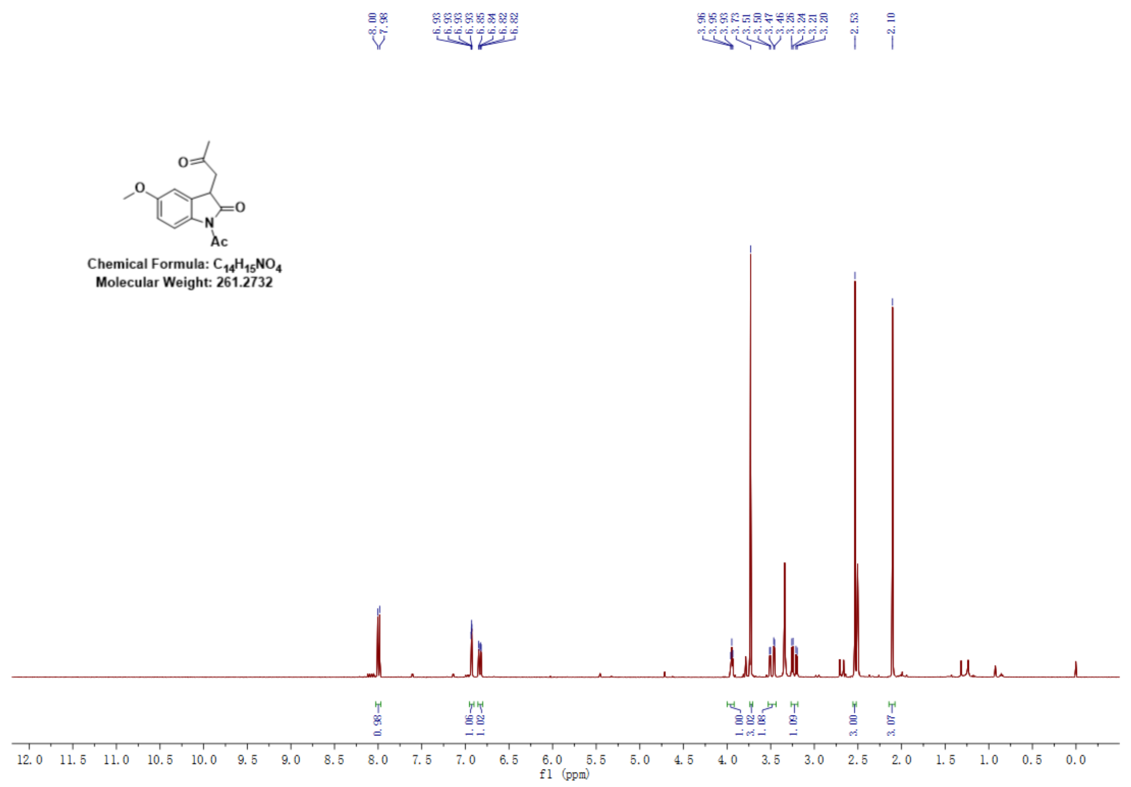

1-Acetyl-5-fluoro-3-(2-oxopropyl)indolin-2-one **(1n)**

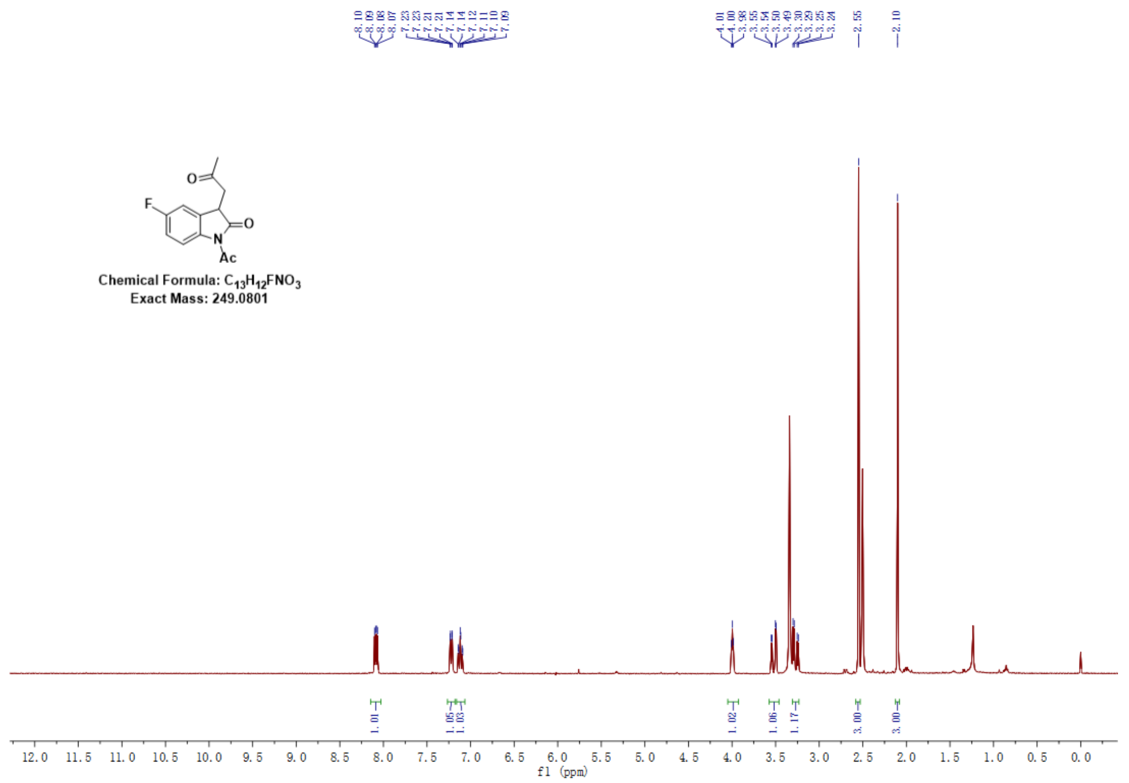

1-Acetyl-7-fluoro-3-(2-oxopropyl)indolin-2-one **(1o)**

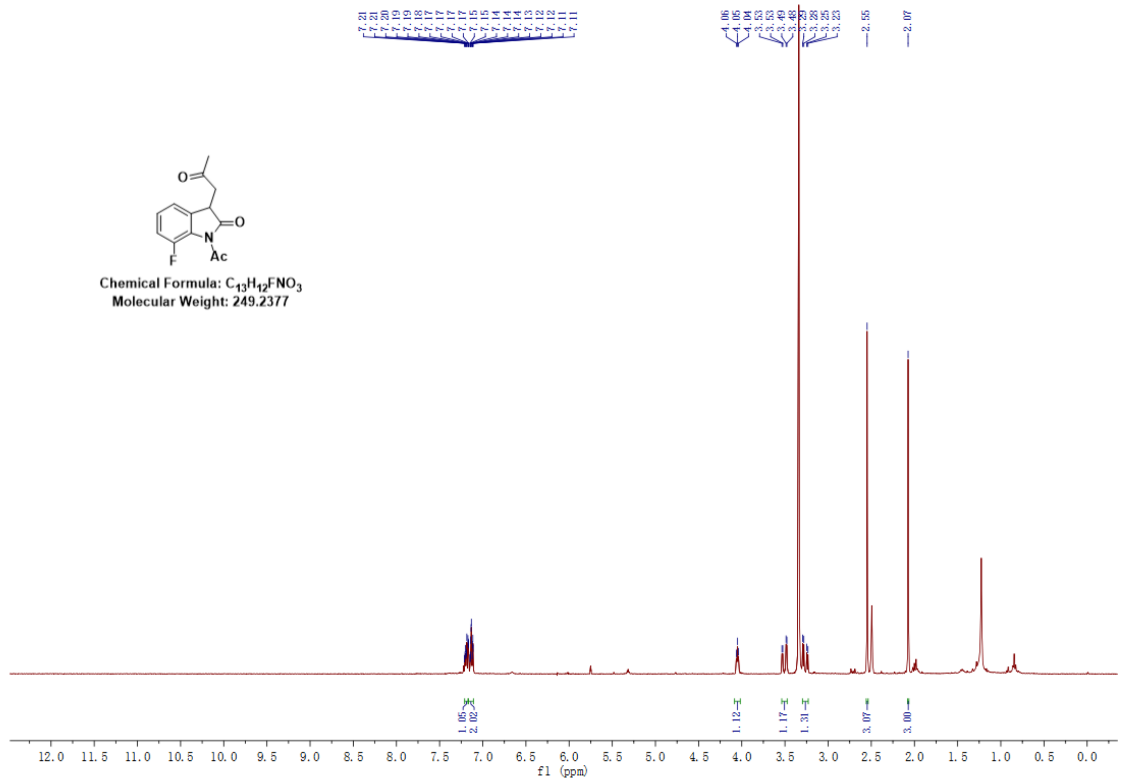

(1*S*,2*R*,3*S*,4*R*)-*tert*-Butyl-2-acetamido-4-hydroxy-4-methyl-3-nitro-2'-oxospiro[cyclopentane-1,3'-indoline]-1'-carboxylate **(3a)**

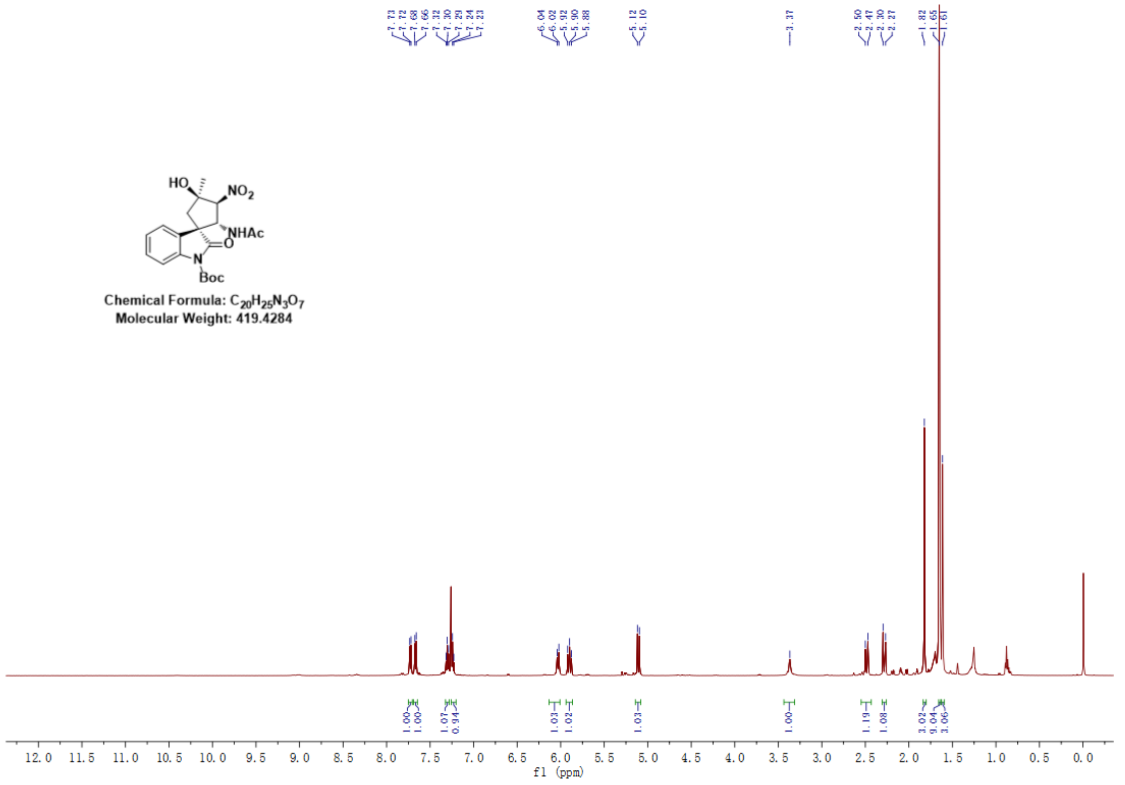

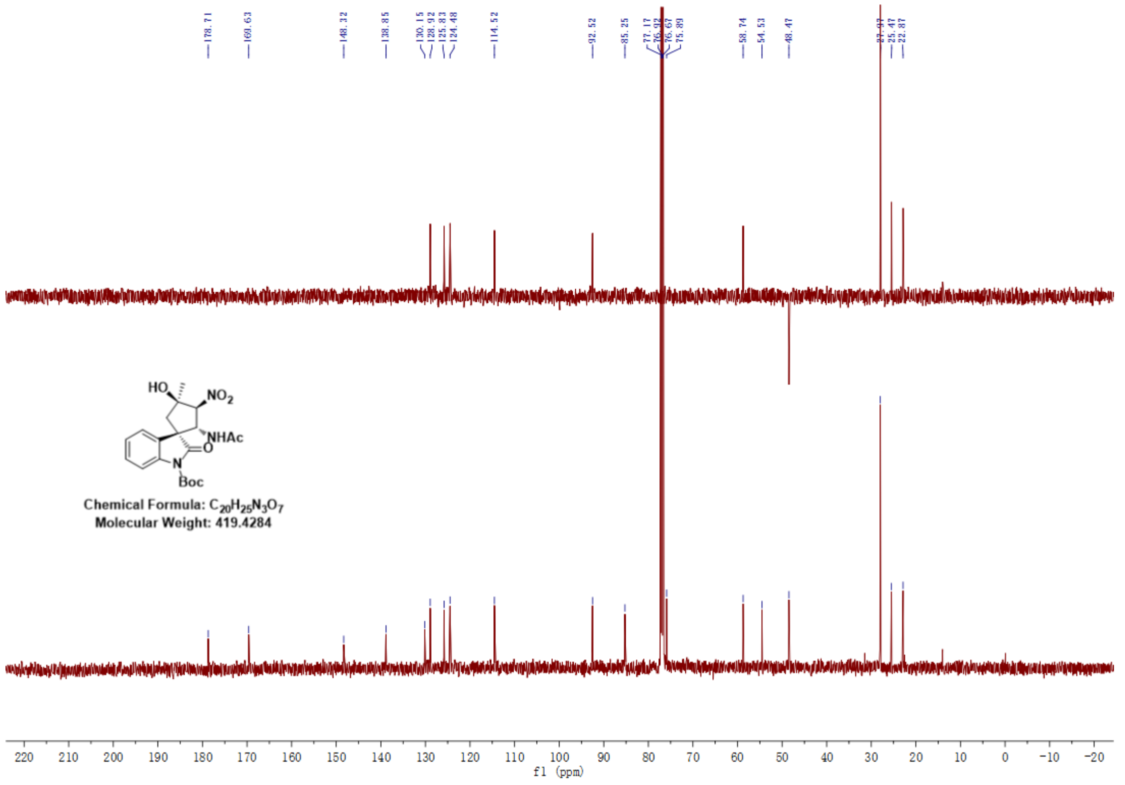

(1*S*,2*R*,3*S*,4*R*)-*tert*-Butyl-2-acetamido-4-hydroxy-4,5'-dimethyl-3-nitro-2'-oxospiro[cyclopentane-1,3'-indoline]-1'-carboxylate **(3b)**

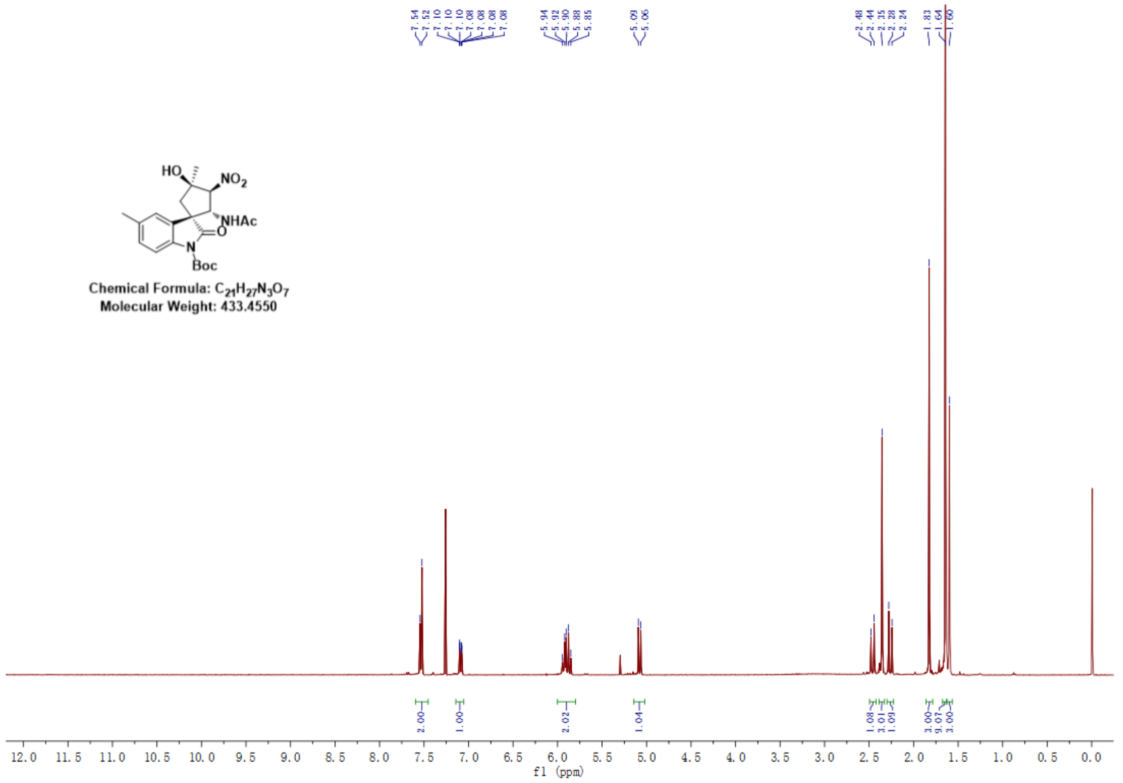

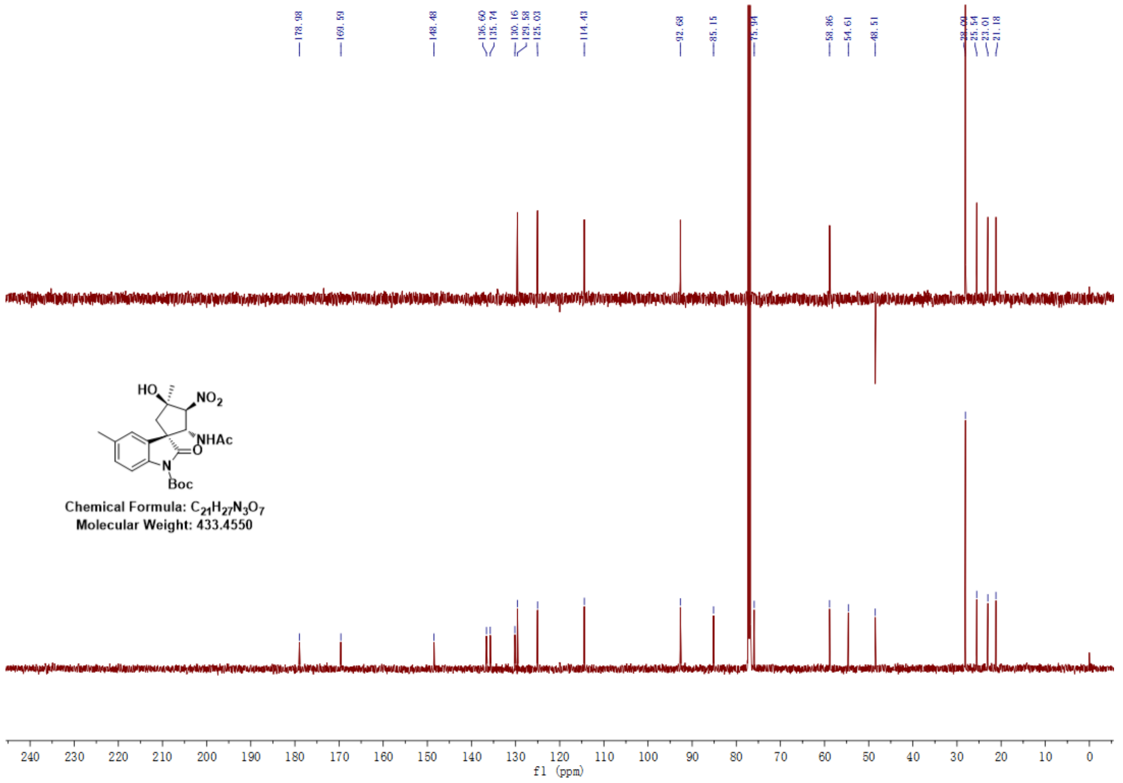

(1*S*,2*R*,3*S*,4*R*)-*tert*-Butyl-2-acetamido-4-hydroxy-4,5',7'-trimethyl-3-nitro-2'-oxospiro [cyclopentane-1,3'-indoline]-1'-carboxylate **(3c)**

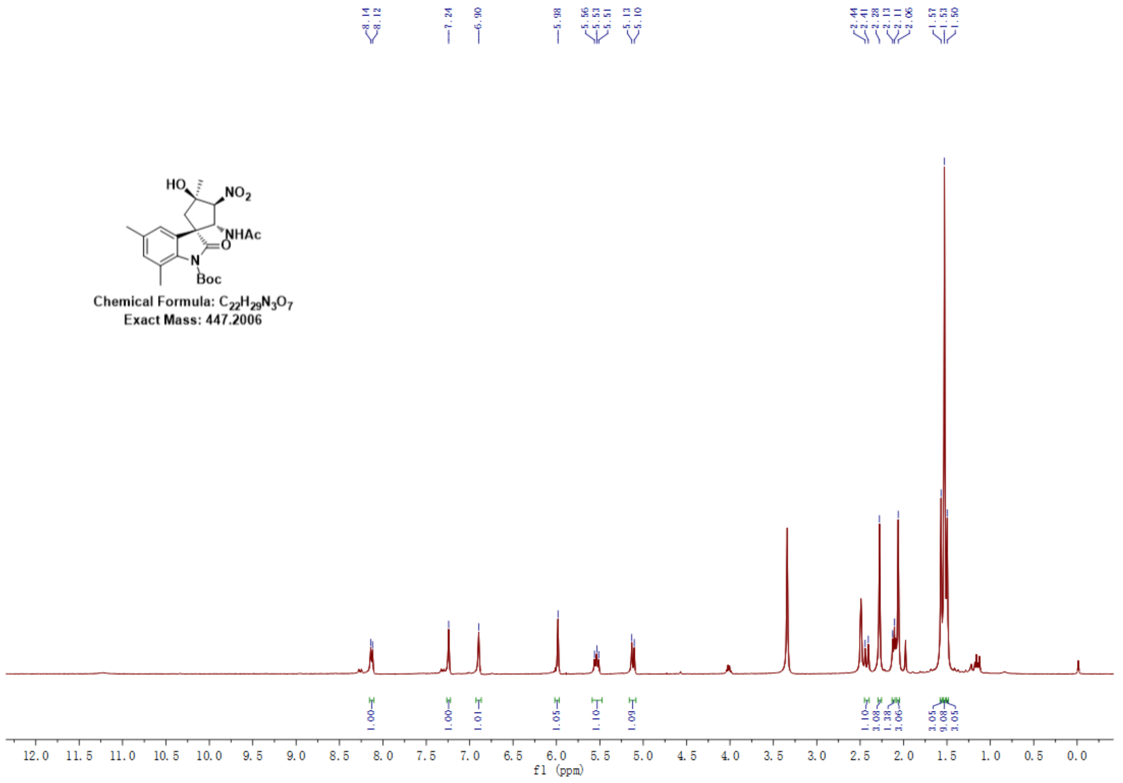

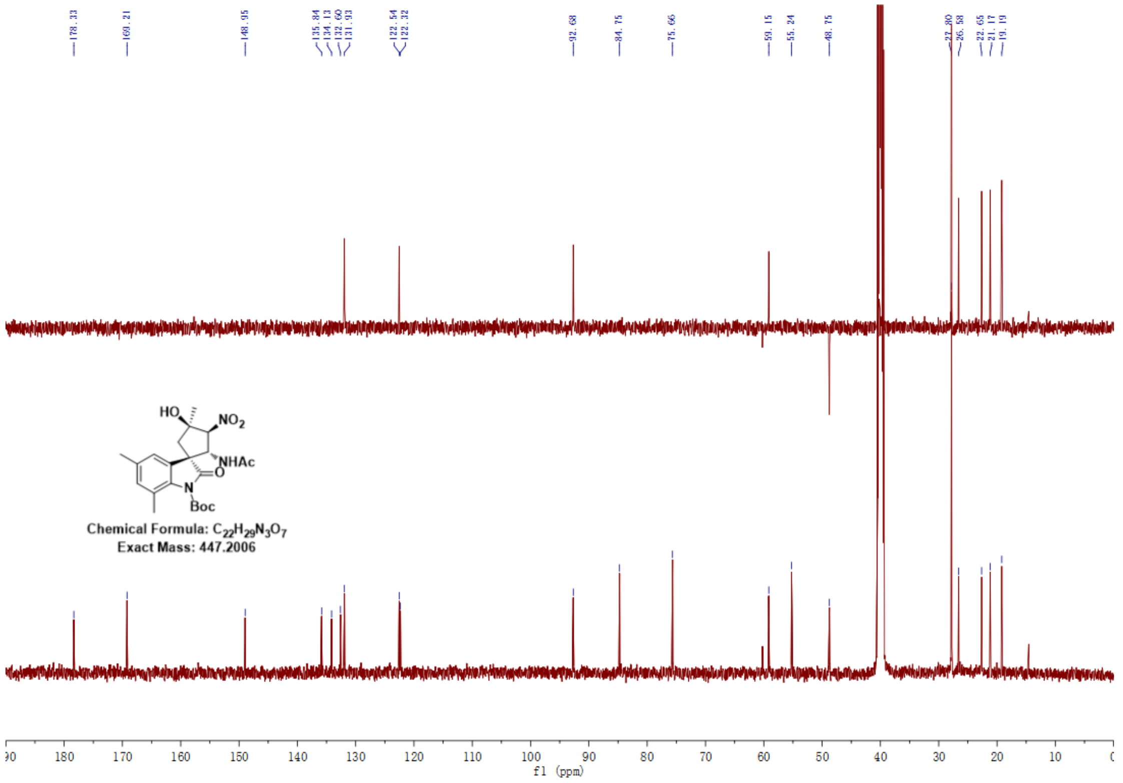

(1*S*,2*R*,3*S*,4*R*)-*tert*-Butyl-2-acetamido-4-hydroxy-5'-methoxy-4-methyl-3-nitro-2'-oxo spiro[cyclopentane-1,3'-indoline]-1'-carboxylate **(3d)**

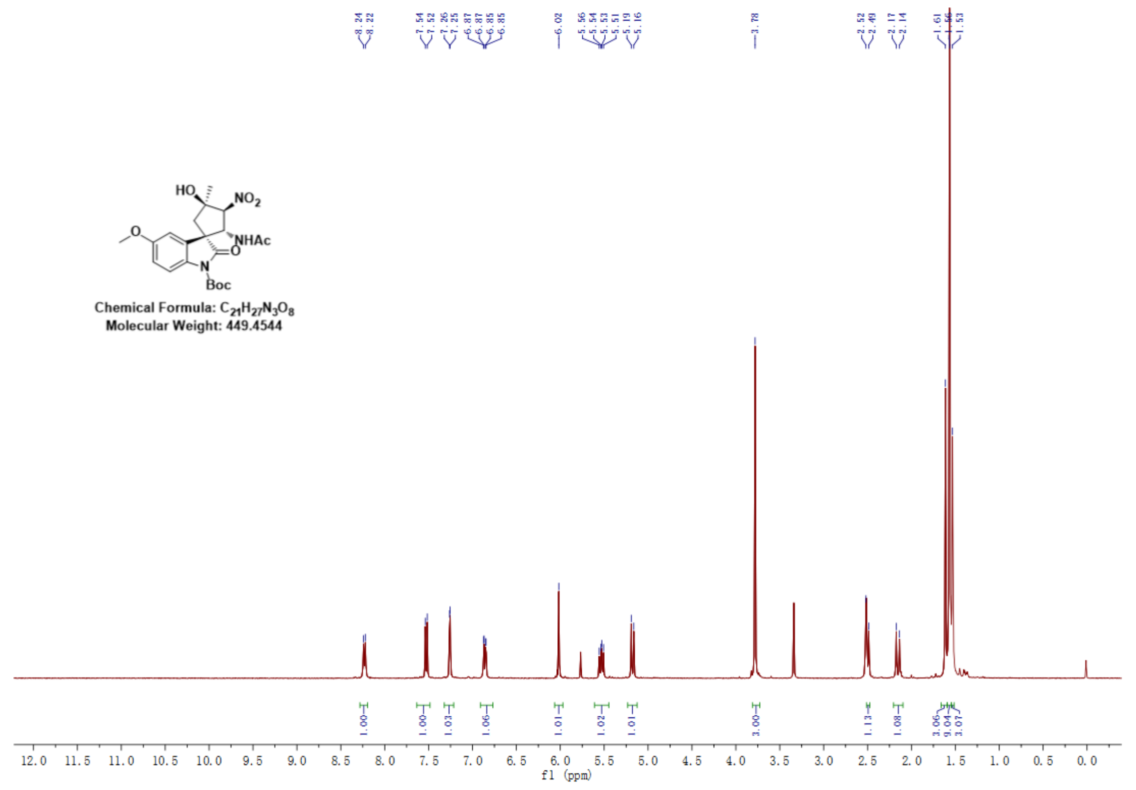

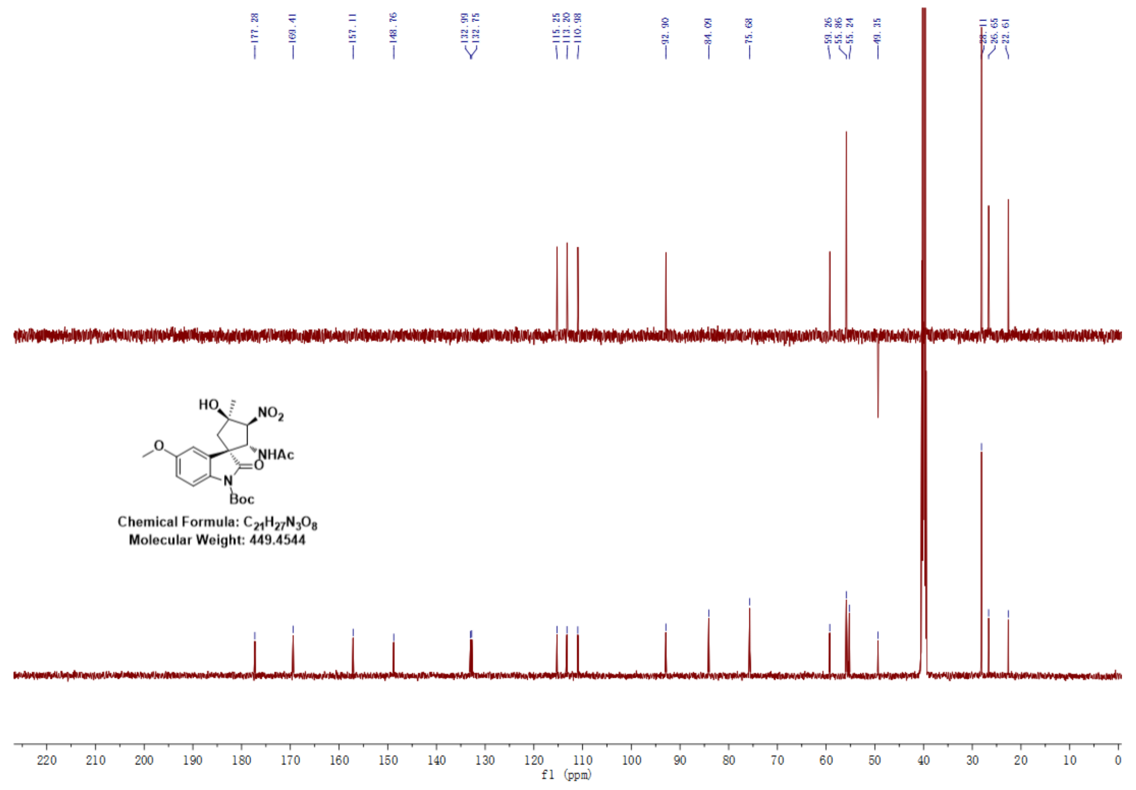

(1*S*,2*R*,3*S*,4*R*)-*tert*-Butyl-2-acetamido-4-hydroxy-5'-trifluoromethoxy-4-methyl-3-nitro-2'-oxospiro[cyclopentane-1,3'-indoline]-1'-carboxylate **(3e)**

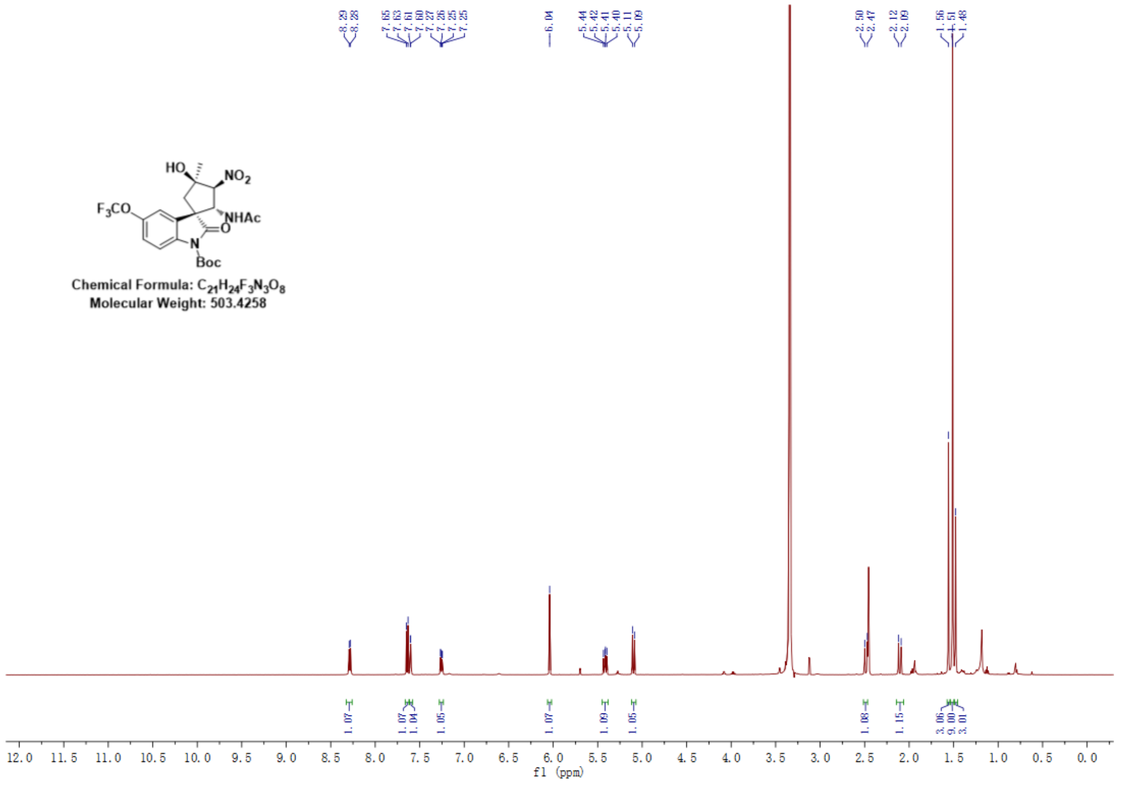

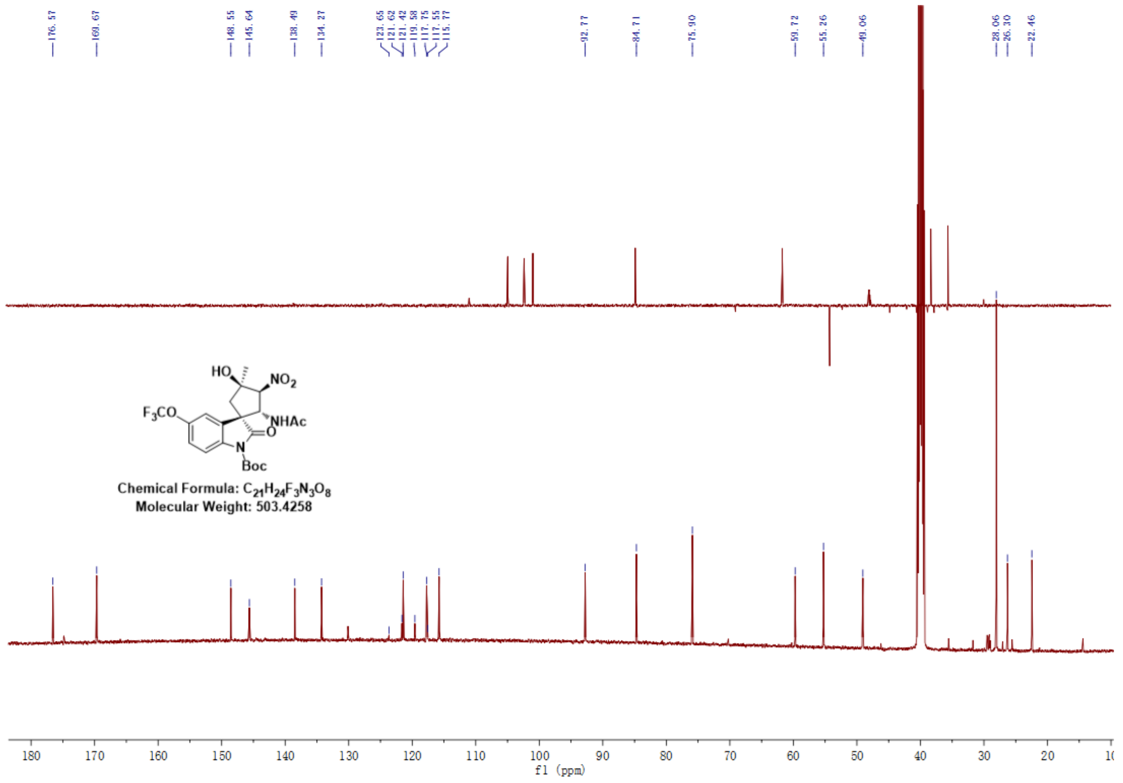

(1*S*,2*R*,3*S*,4*R*)-*tert*-Butyl-2-acetamido-4-hydroxy-5'-fluoro-4-methyl-3-nitro-2'-oxospiro[cyclopentane-1,3'-indoline]-1'-carboxylate **(3f)**

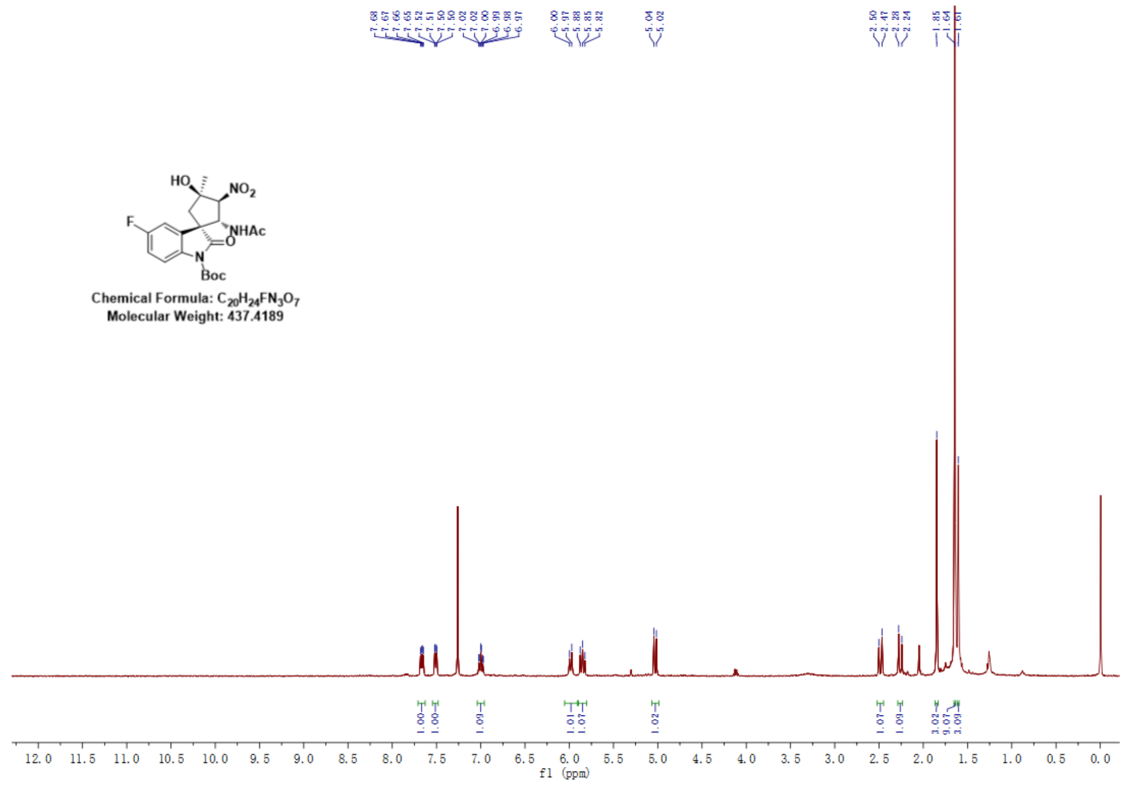

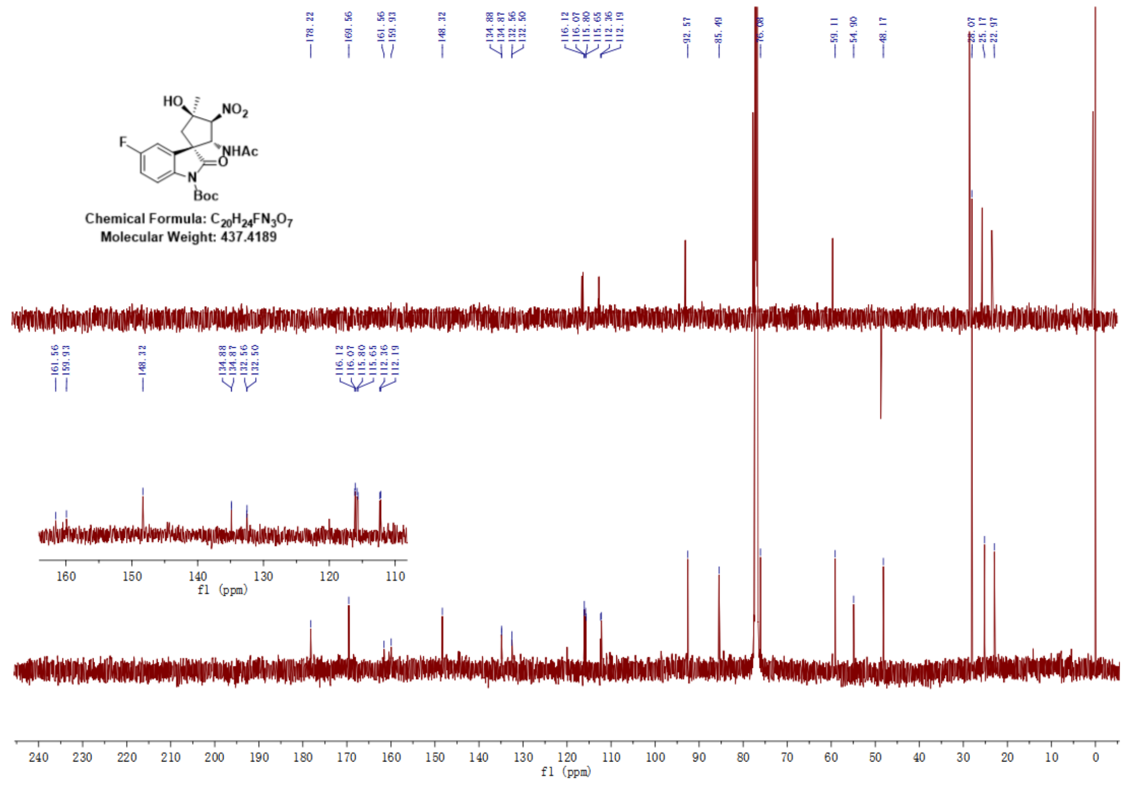

(1*S*,2*R*,3*S*,4*R*)-*tert*-Butyl-2-acetamido-4-hydroxy-5'-chloro-4-methyl-3-nitro-2'-oxospiro[cyclopentane-1,3'-indoline]-1'-carboxylate **(3g)**

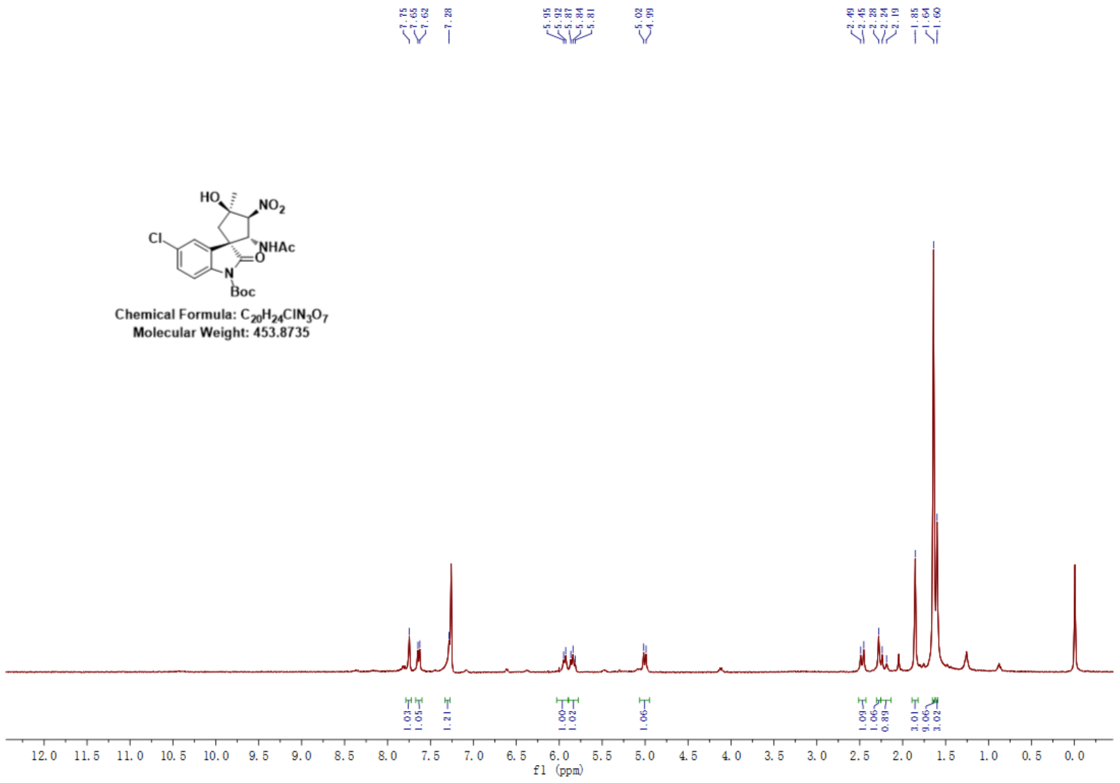

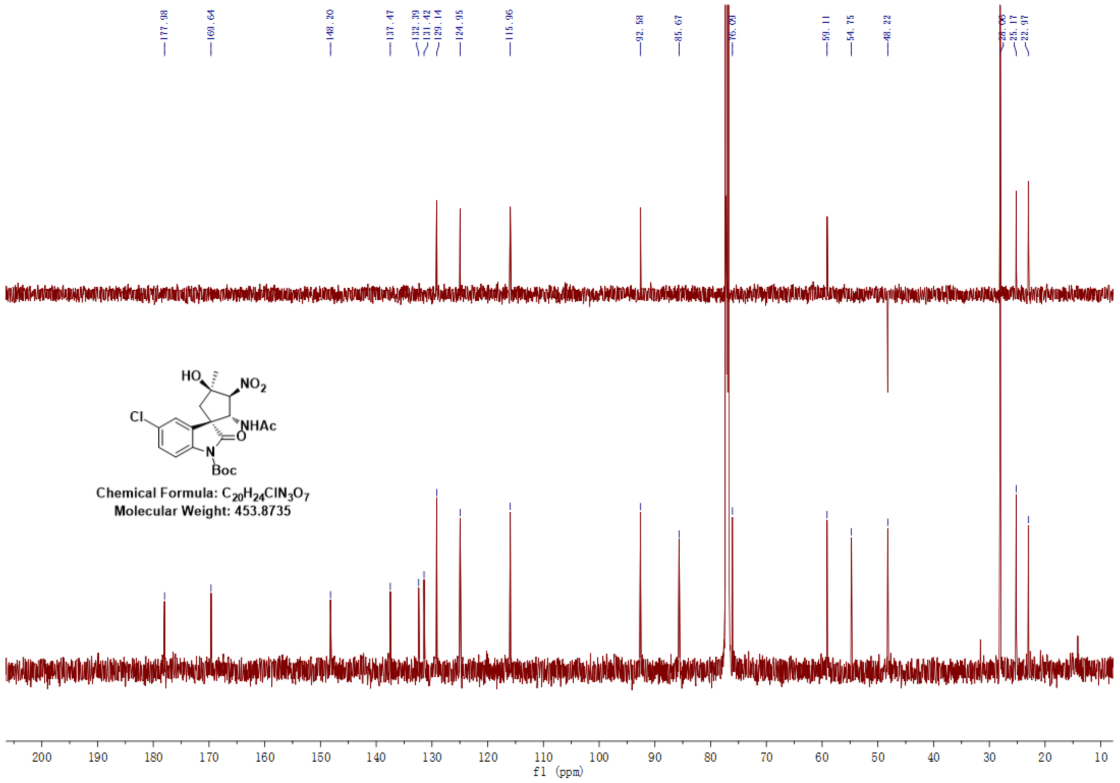

(1*S*,2*R*,3*S*,4*R*)-*tert*-Butyl-2-acetamido-4-hydroxy-7'-fluoro-4-methyl-3-nitro-2'-oxospiro[cyclopentane-1,3'-indoline]-1'-carboxylate **(3h)**

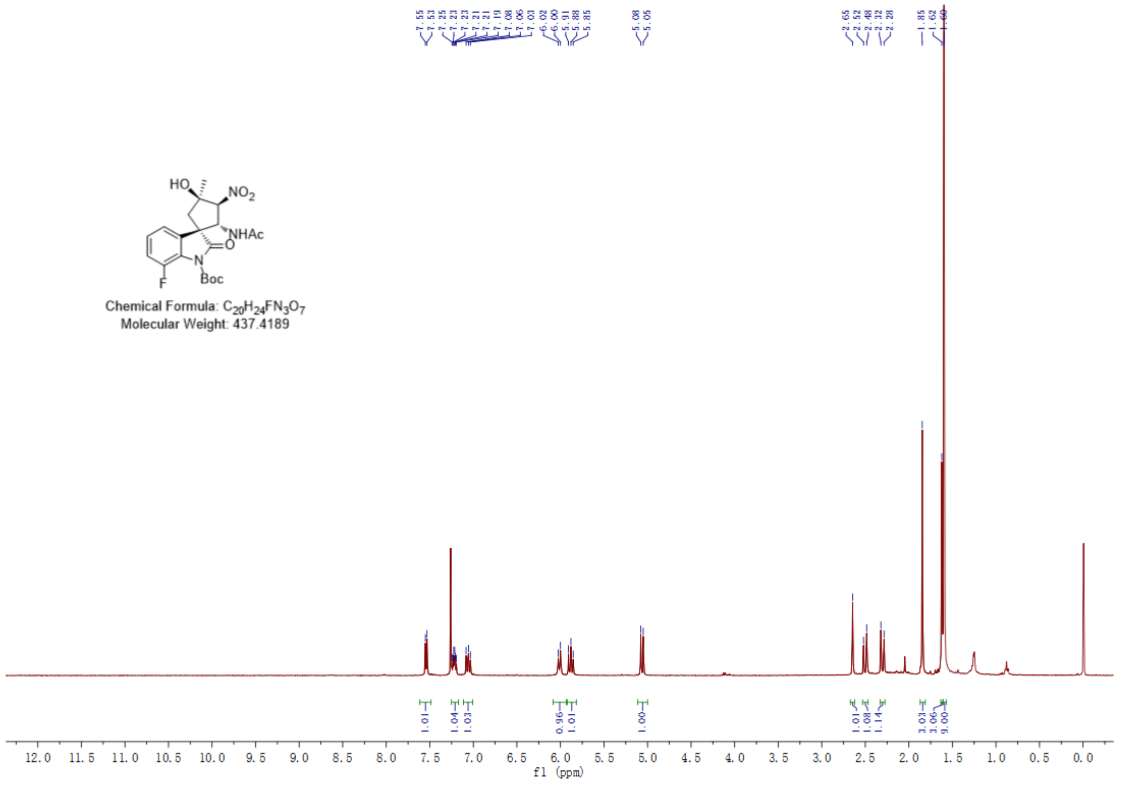

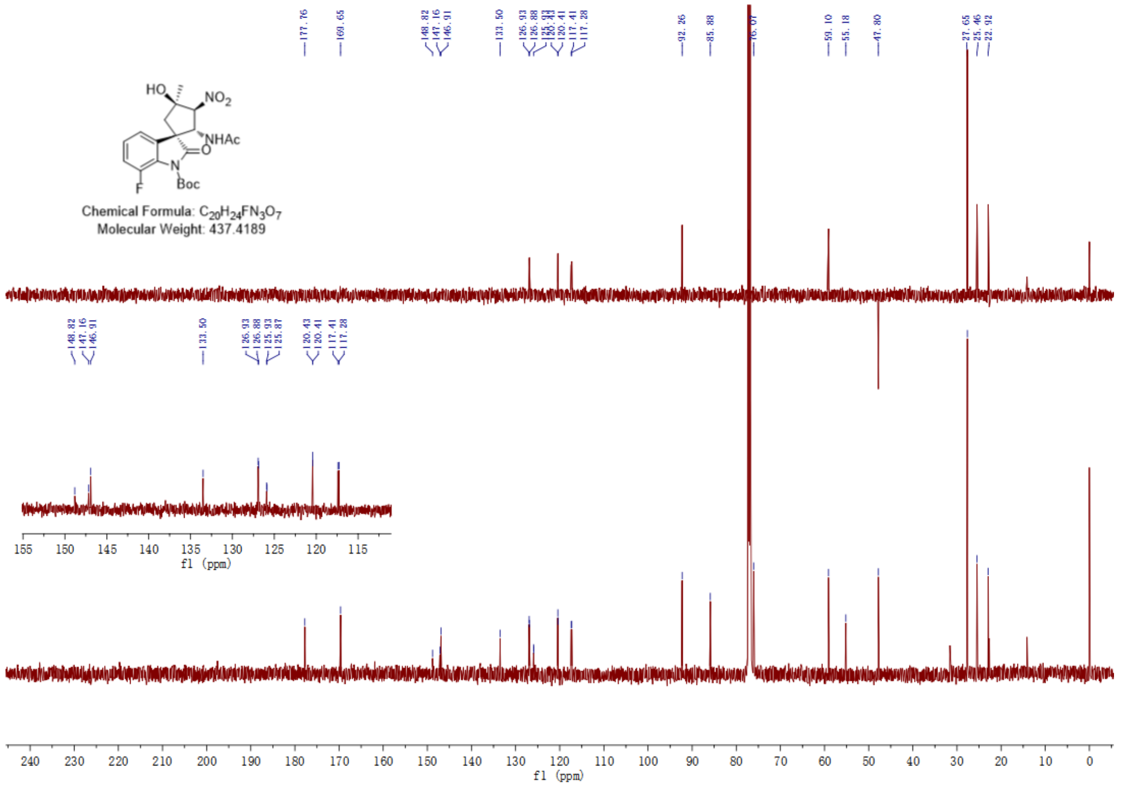

(1*S*,2*R*,3*S*,4*R*)-*tert*-Butyl-2-acetamido-4-hydroxy-6'-chloro-4-methyl-3-nitro-2'-oxospiro[cyclopentane-1,3'-indoline]-1'-carboxylate **(3i)**

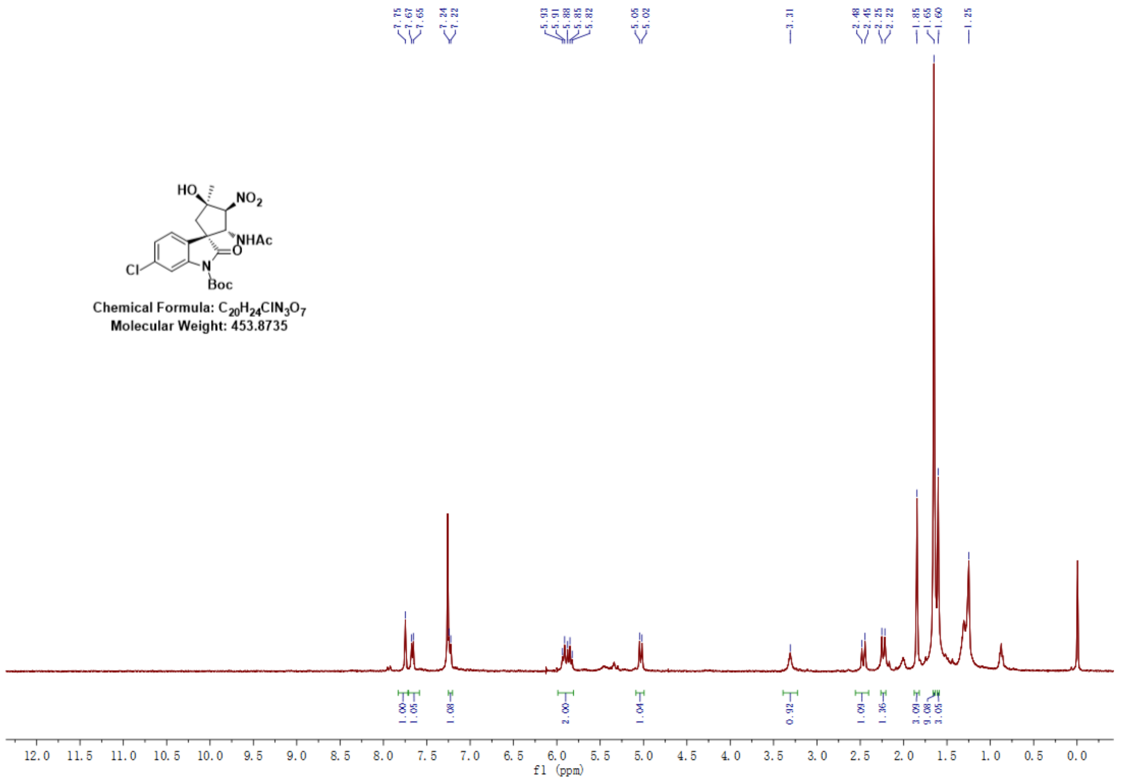

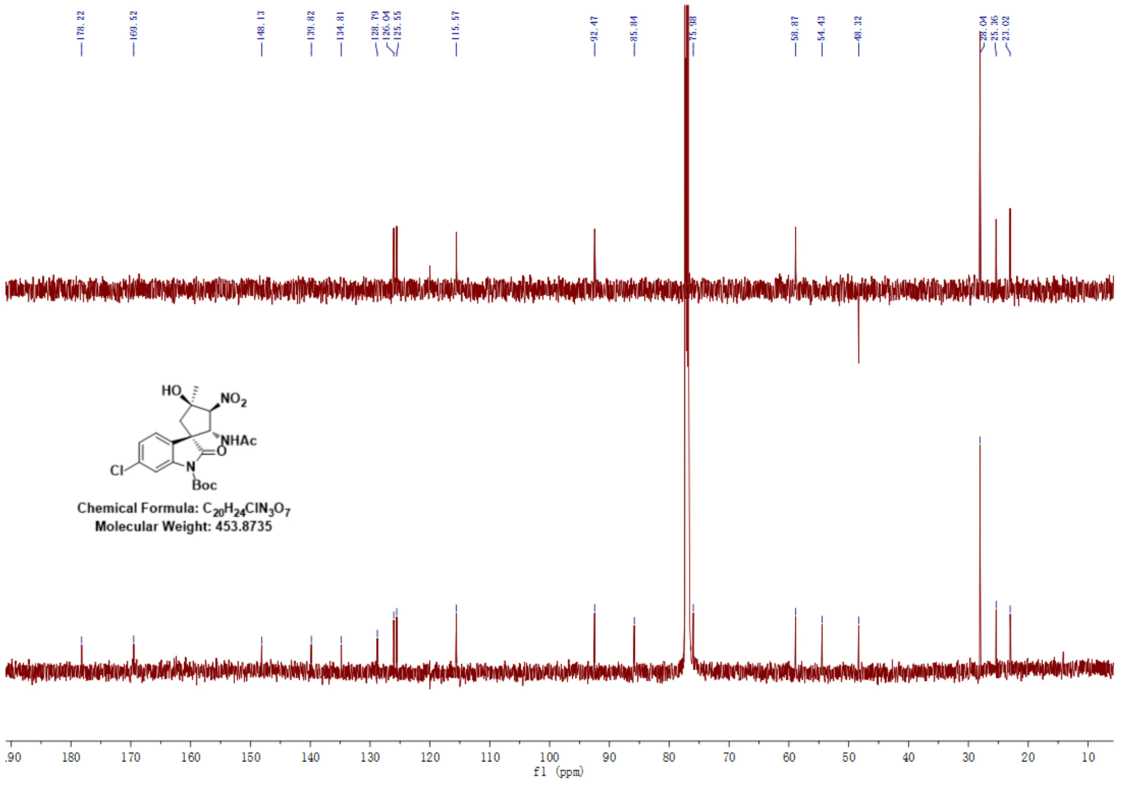

(1*S*,2*R*,3*S*,4*R*)-*tert*-Butyl-2-acetamido-4-hydroxy-6'-bromo-4-methyl-3-nitro-2'-oxospiro[cyclopentane-1,3'-indoline]-1'-carboxylate **(3j)**

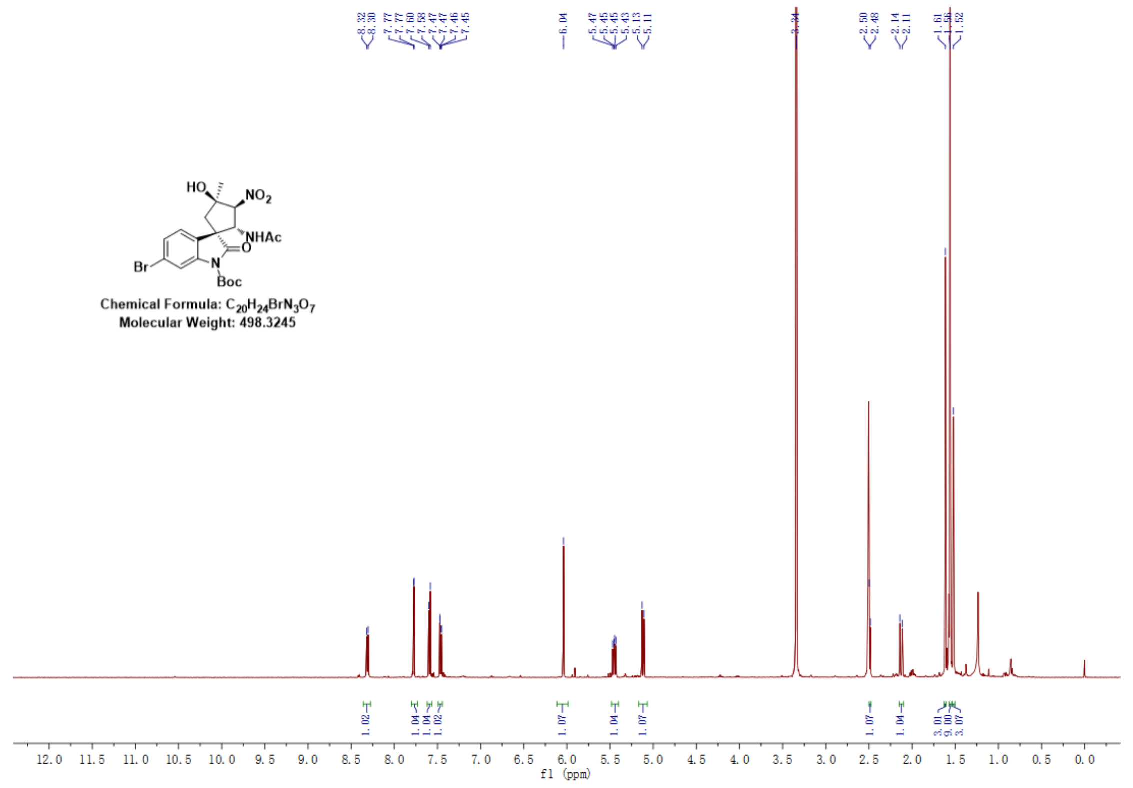

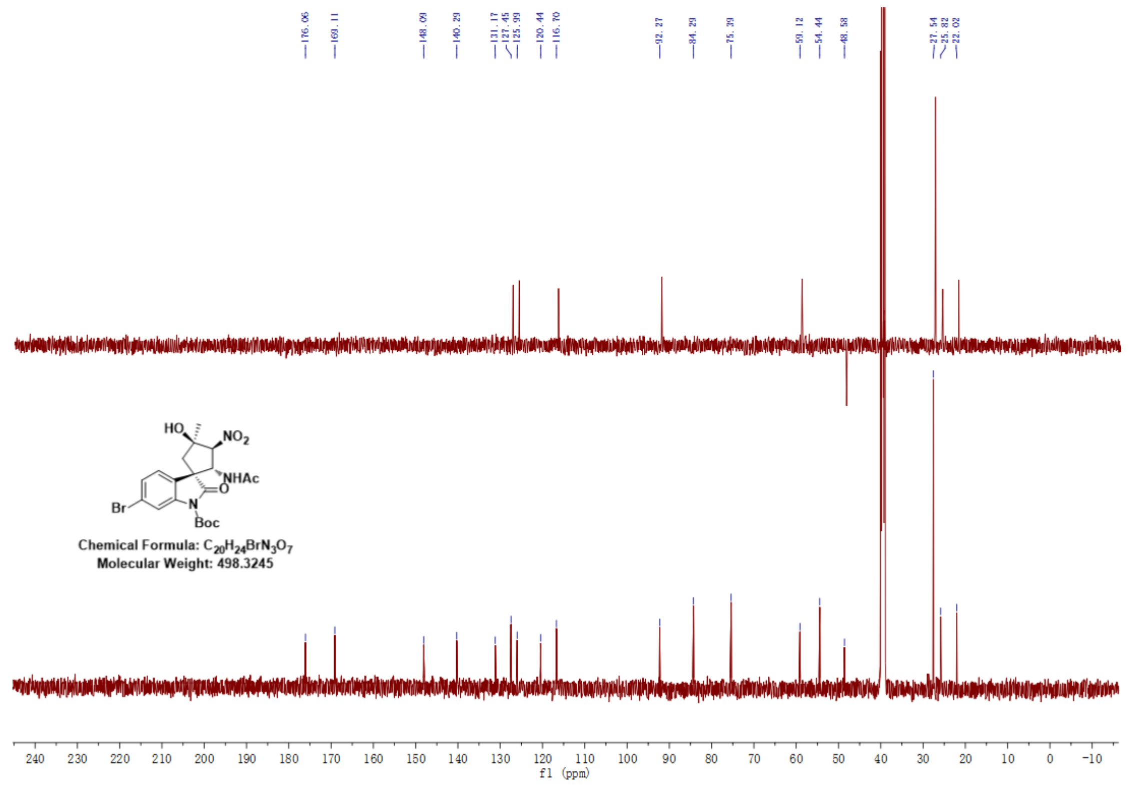

N-((1*S*,2*R*,3*S*,4*R*)-1'-Acetyl-3-hydroxy-3-methyl-4-nitro-2'-oxospiro[cyclopentane-1,3'-indolin]-5-yl)acetamide **(3k)**

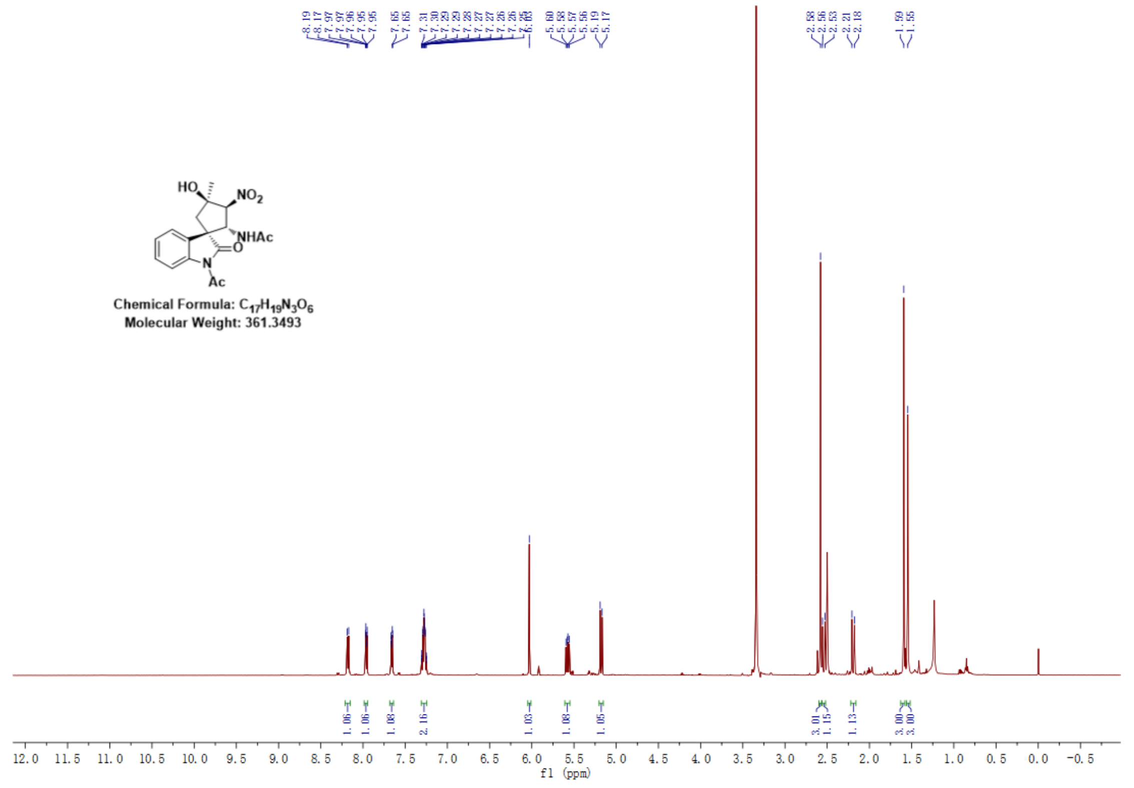

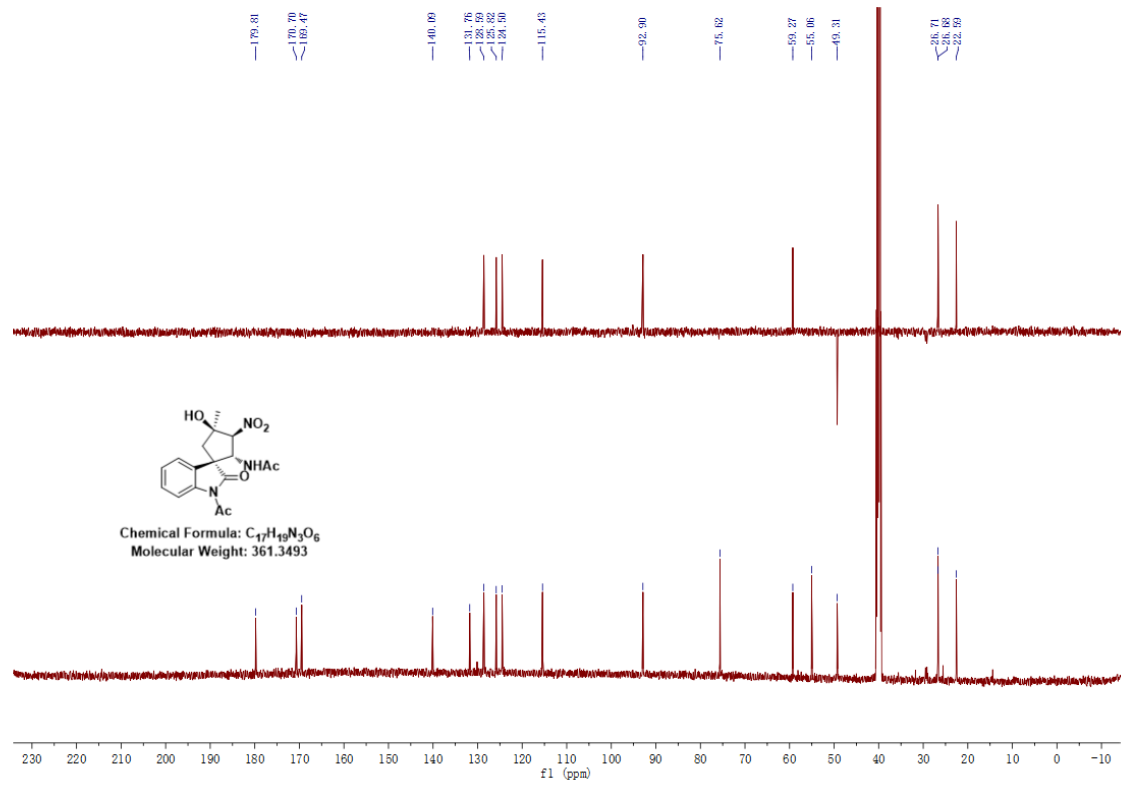

N-((1*S*,2*R*,3*S*,4*R*)-1'-Acetyl-3-hydroxy-3,5'-dimethyl-4-nitro-2'-oxospiro[cyclopentane-1,3'-indolin]-5-yl)acetamide **(3l)**

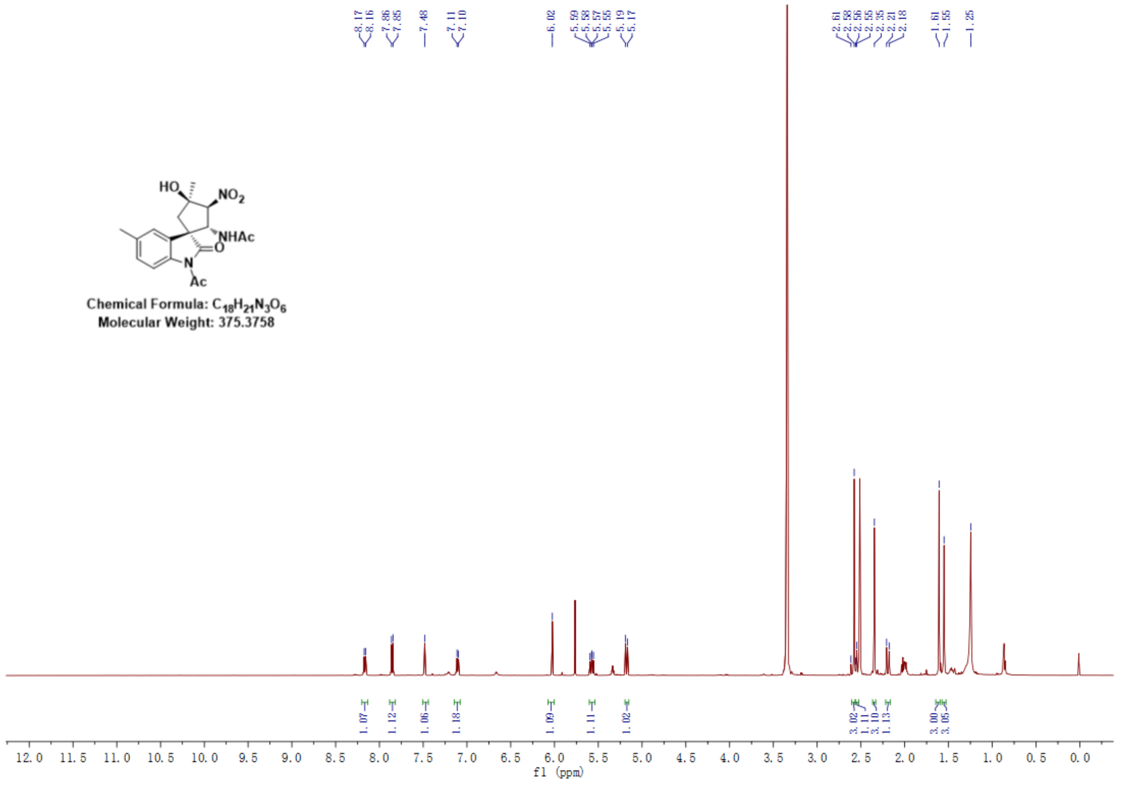

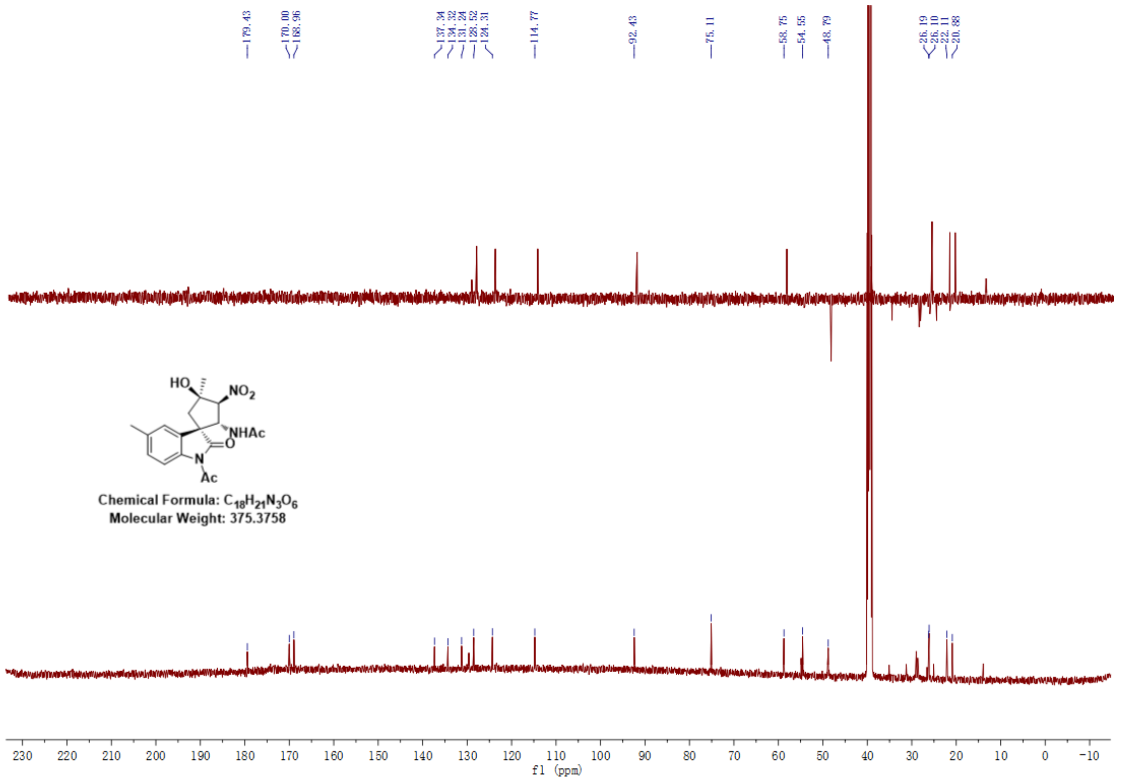

N-((1*S*,2*R*,3*S*,4*R*)-1'-Acetyl-3-hydroxy-5'-methoxy-3-methyl-4-nitro-2'-oxospiro[cyclopentane-1,3'-indolin]-5-yl)acetamide **(3m)**

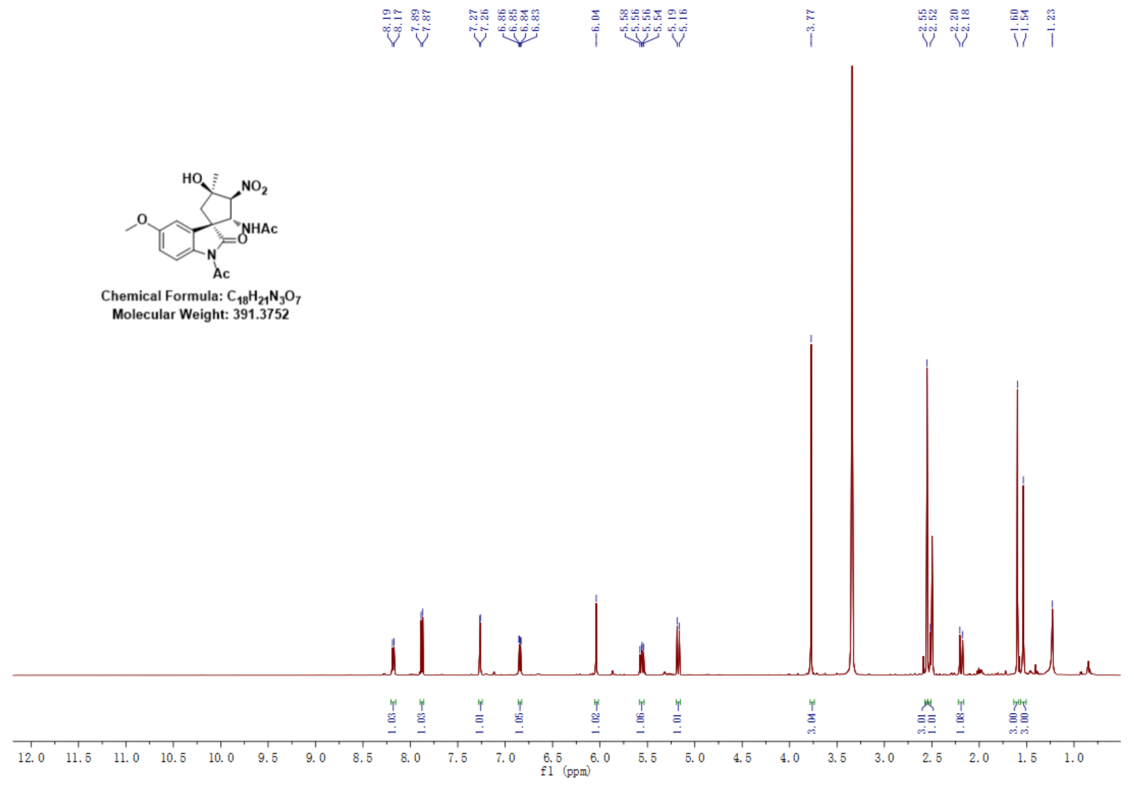

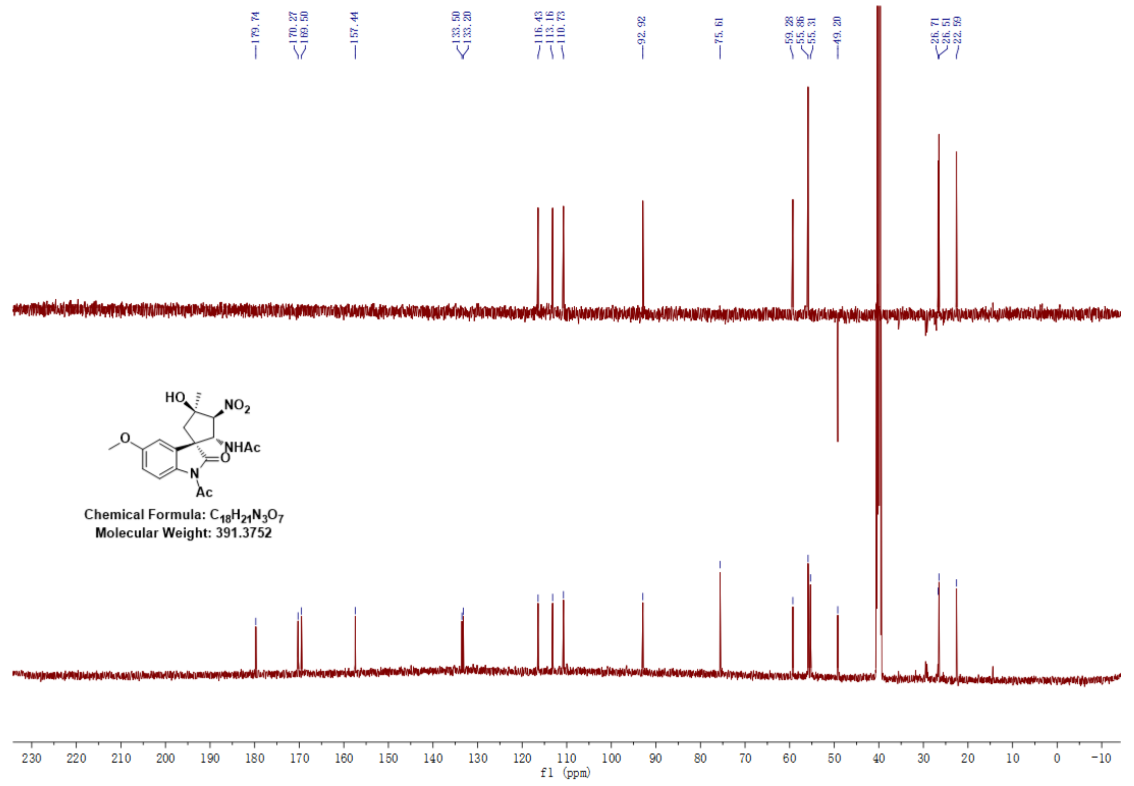

N-((1*S*,2*R*,3*S*,4*R*)-1'-Acetyl-5'-fluoro-3-hydroxy-3-methyl-4-nitro-2'-oxospiro[cyclopentane-1,3'-indolin]-5-yl)acetamide **(3n)**

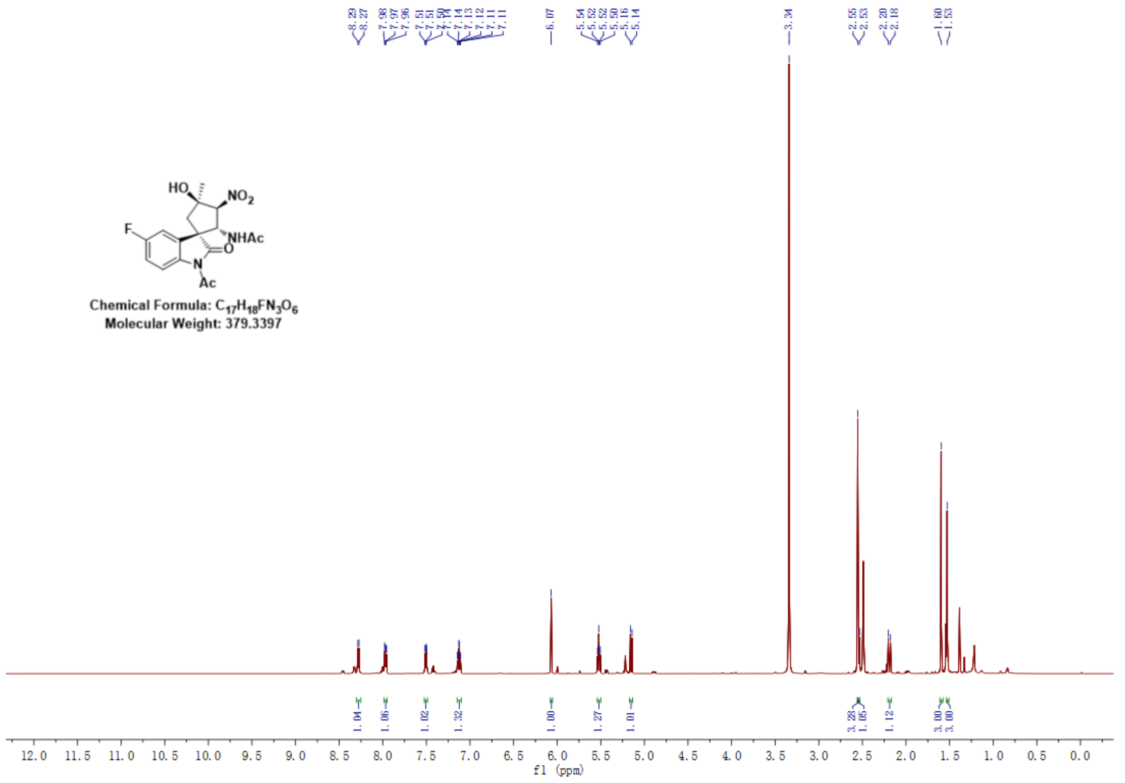

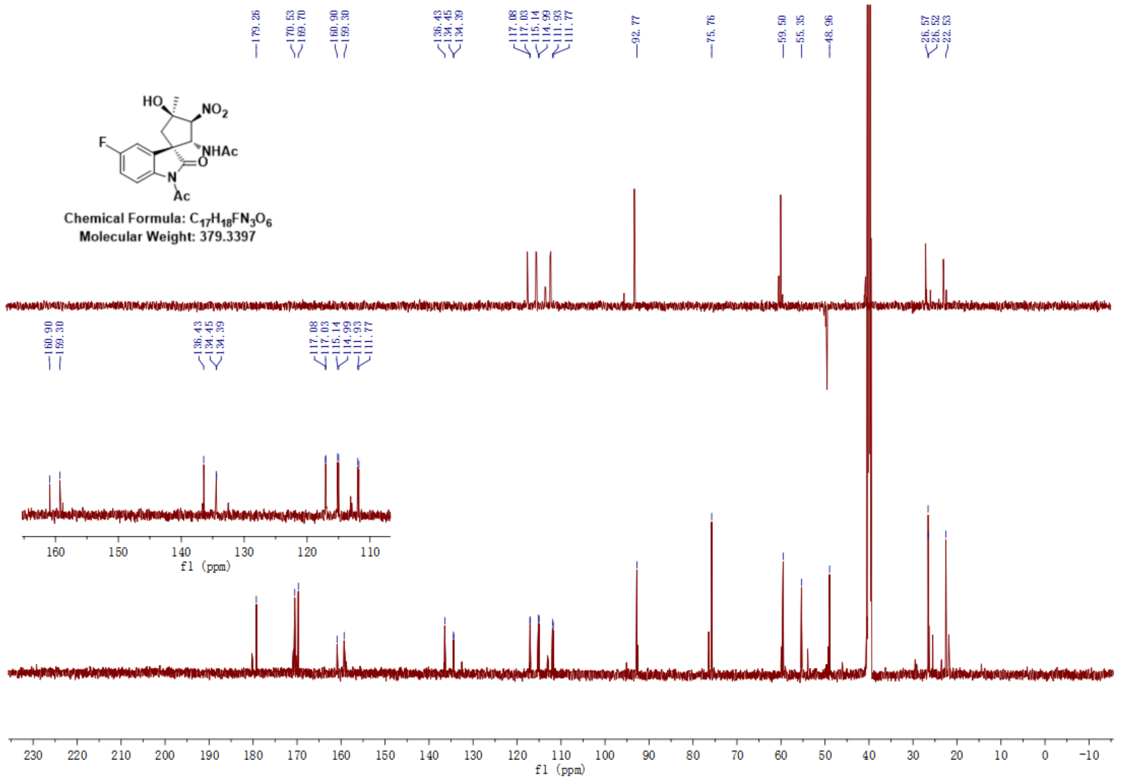

N-((1*S*,2*R*,3*S*,4*R*)-1'-Acetyl-7'-fluoro-3-hydroxy-3-methyl-4-nitro-2'-oxospiro[cyclopentane-1,3'-indolin]-5-yl)acetamide **(3o)**

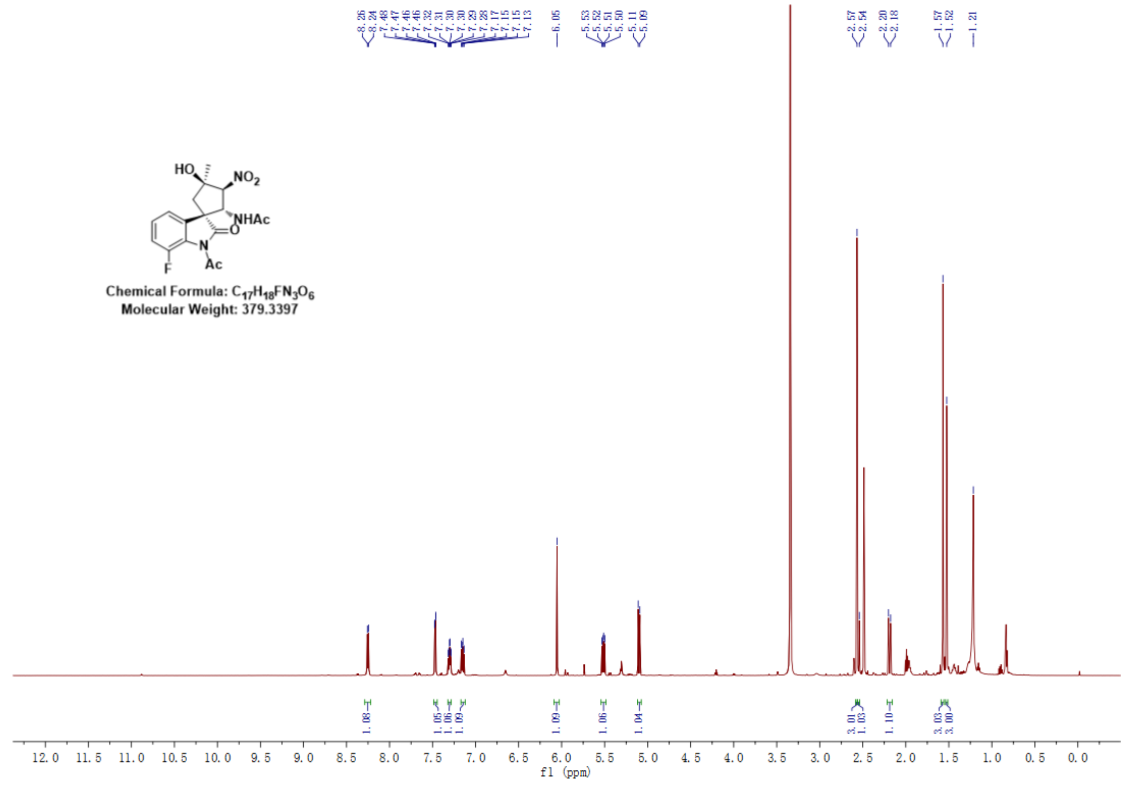

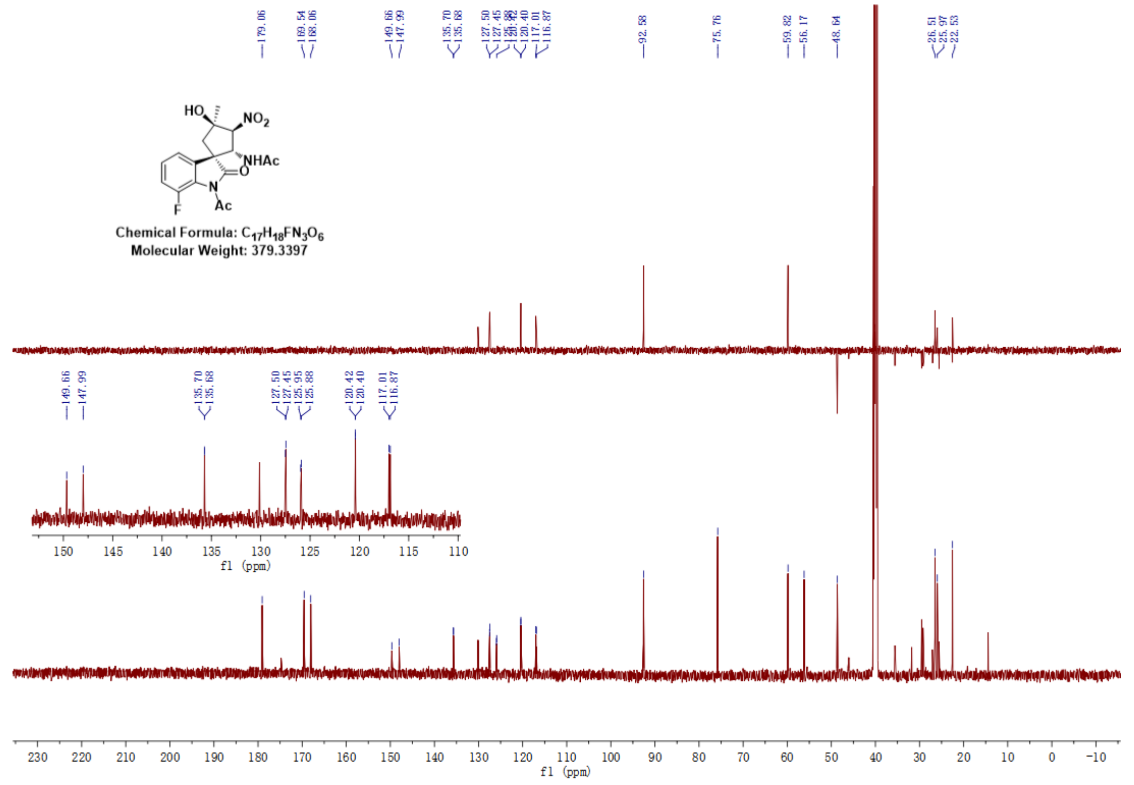

(C) HPLC spectra

(1*S*,2*R*,3*S*,4*R*)-*tert*-Butyl-2-acetamido-4-hydroxy-4-methyl-3-nitro-2'-oxospiro[cyclopentane-1,3'-indoline]-1'-carboxylate **(3a)**

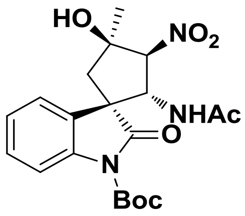

3a

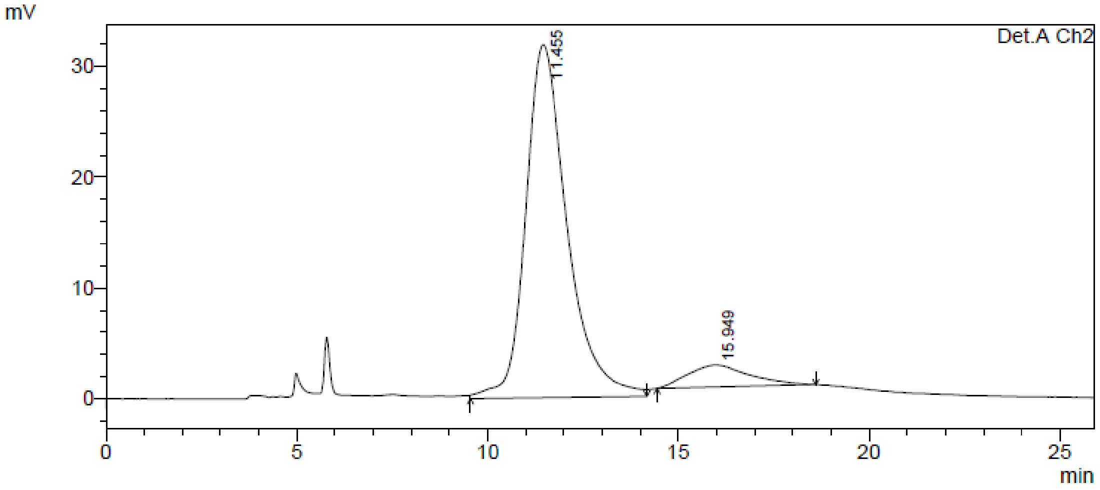

Peak#	Ret. Time	Area	Height	Area %	Height %
1	11.455	2416331	31859	91.344	94.060
2	15.949	228966	2012	8.656	5.940
Total		2645296	33871	100.000	100.000

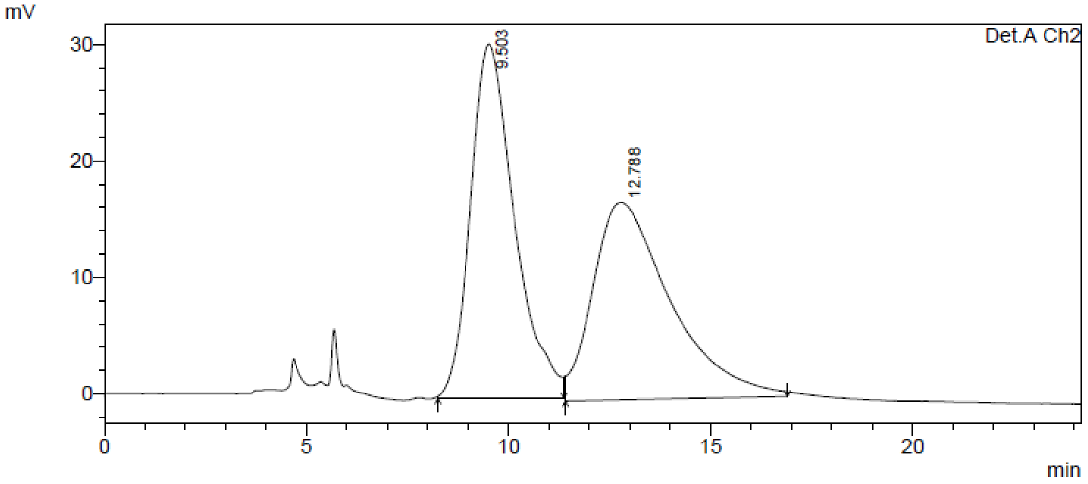

Peak#	Ret. Time	Area	Height	Area %	Height %
1	9.503	2305699	30443	50.467	64.217
2	12.788	2263049	16964	49.533	35.783
Total		4568748	47407	100.000	100.000

(1*S*,2*R*,3*S*,4*R*)-*tert*-Butyl-2-acetamido-4-hydroxy-4,5'-dimethyl-3-nitro-2'-oxospiro-[cyclopentane-1,3'-indoline]-1'-carboxylate **(3b)**

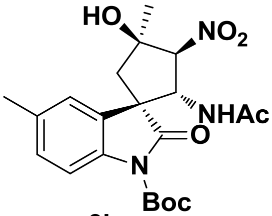

3b

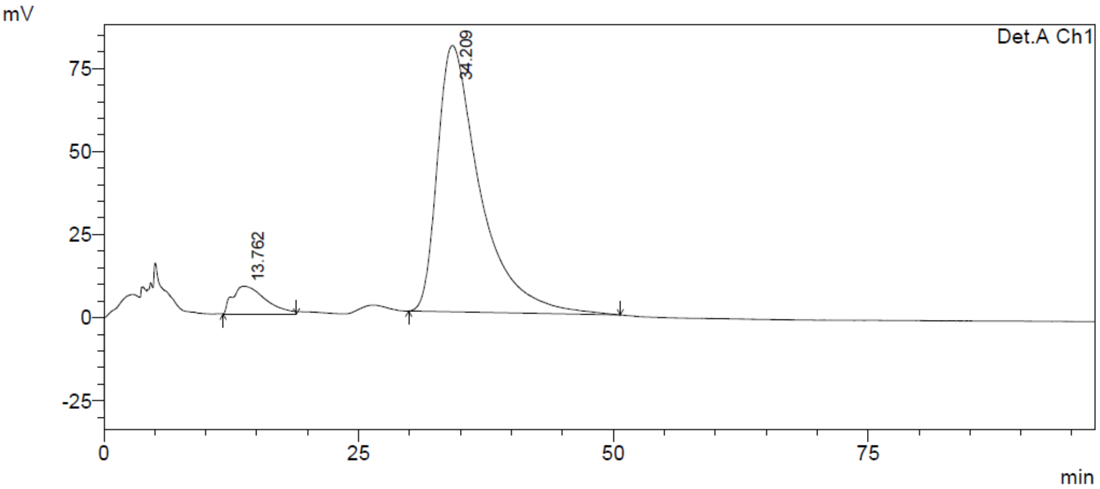

Peak#	Ret. Time	Area	Height	Area %	Height %
1	13.762	1893034	8342	7.478	9.423
2	34.209	23421722	80186	92.522	90.577
Total		25314756	88528	100.000	100.000

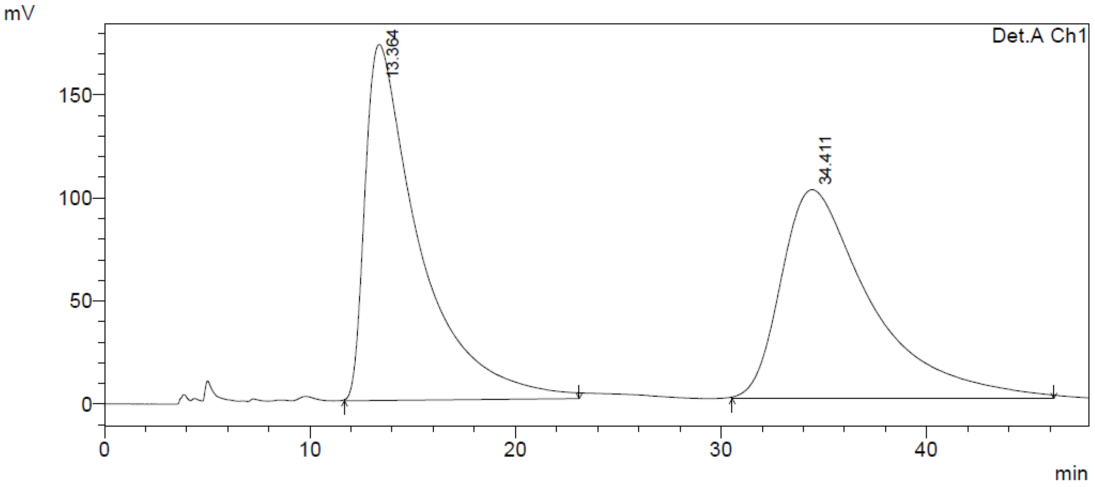

Peak#	Ret. Time	Area	Height	Area %	Height %
1	13.364	31814948	172824	50.669	63.046
2	34.411	30975013	101302	49.331	36.954
Total		62789961	274126	100.000	100.000

(1*S*,2*R*,3*S*,4*R*)-*tert*-Butyl-2-acetamido-4-hydroxy-4,5',7'-trimethyl-3-nitro-2'-oxospiro [cyclopentane-1,3'-indoline]-1'-carboxylate **(3c)**

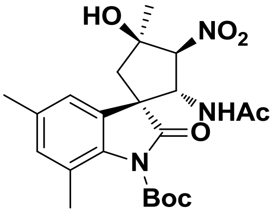

3c

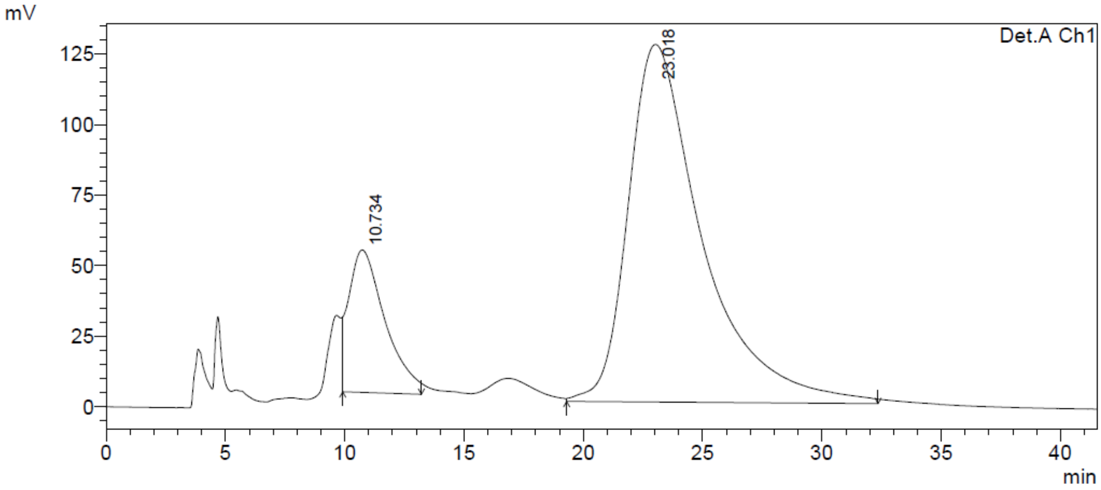

Peak#	Ret. Time	Area	Height	Area %	Height %
1	10.734	5340200	50464	16.301	28.482
2	23.018	27420267	126711	83.699	71.518
Total		32760466	177175	100.000	100.000

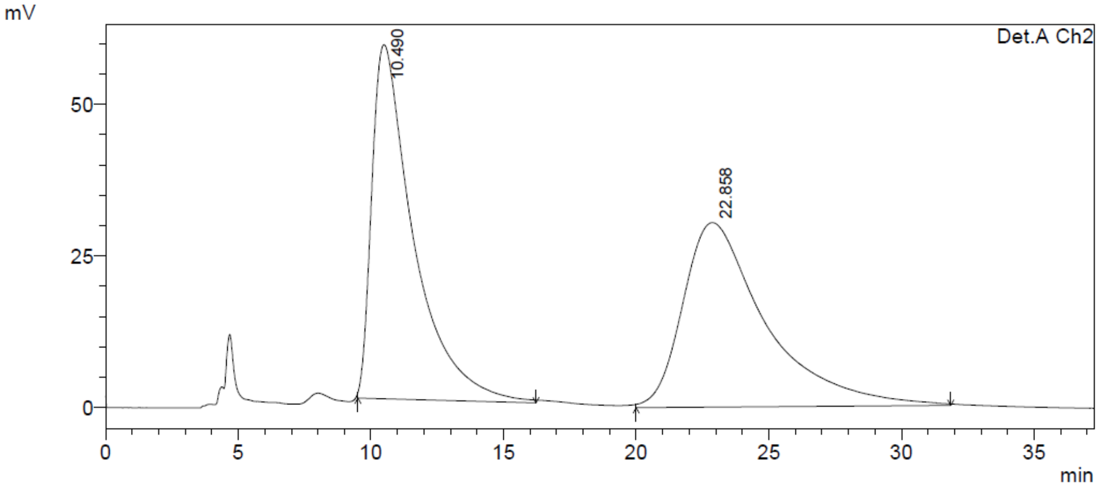

Peak#	Ret. Time	Area	Height	Area %	Height %
1	10.490	6380640	58387	49.090	65.753
2	22.858	6617080	30410	50.910	34.247
Total		12997719	88797	100.000	100.000

(1*S*,2*R*,3*S*,4*R*)-*tert*-Butyl-2-acetamido-4-hydroxy-5'-methoxy-4-methyl-3-nitro-2'-oxo spiro[cyclopentane-1,3'-indoline]-1'-carboxylate **(3d)**

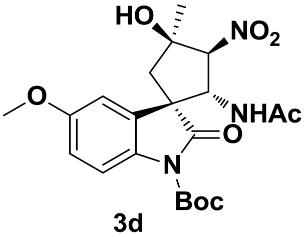

3d

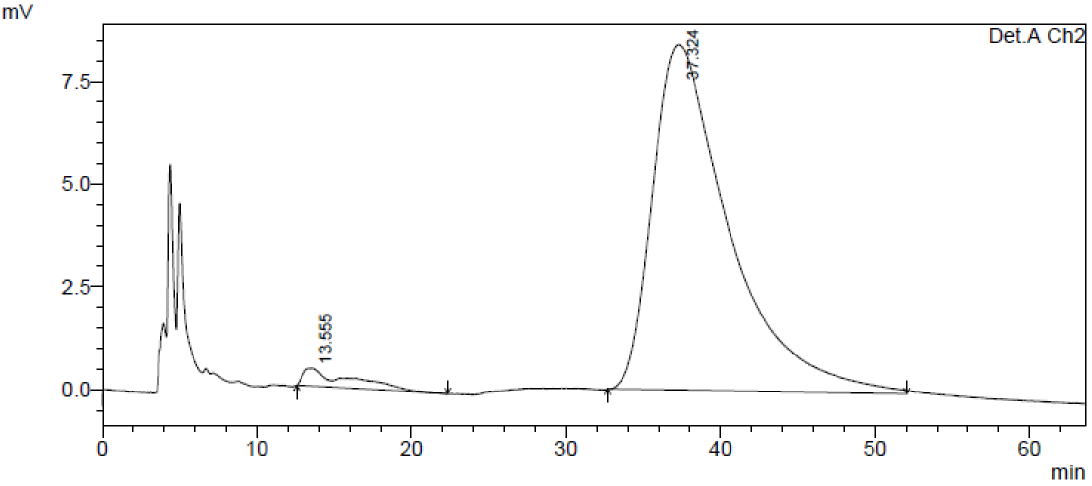

Peak#	Ret. Time	Area	Height	Area %	Height %
1	13.555	93120	439	3.120	4.961
2	37.324	2891125	8405	96.880	95.039
Total		2984245	8843	100.000	100.000

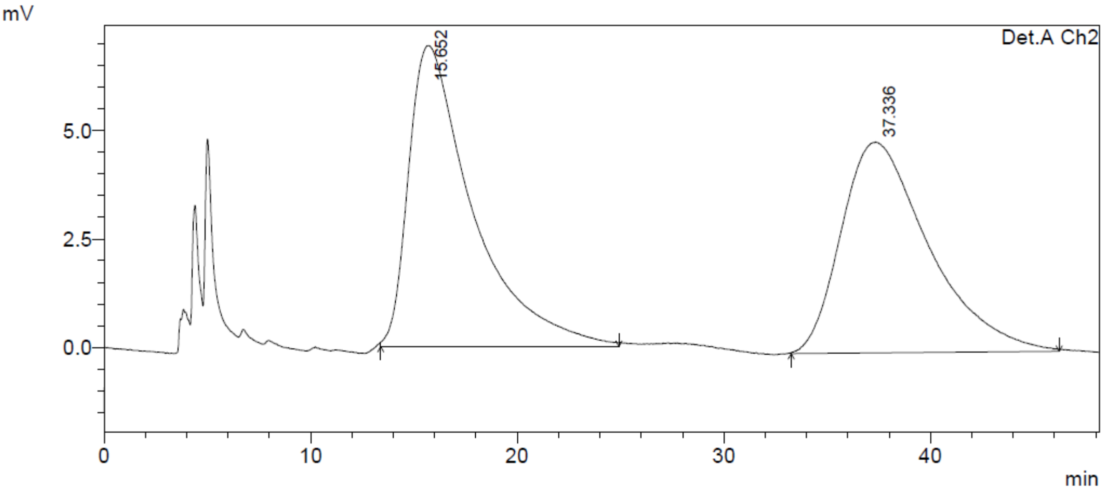

Peak#	Ret. Time	Area	Height	Area %	Height %
1	15.652	1498311	6936	51.069	58.774
2	37.336	1435606	4865	48.931	41.226
Total		2933916	11802	100.000	100.000

(1*S*,2*R*,3*S*,4*R*)-*tert*-Butyl-2-acetamido-4-hydroxy-5'-trifluoromethoxy-4-methyl-3-nitro-2'-oxospiro[cyclopentane-1,3'-indoline]-1'-carboxylate **(3e)**

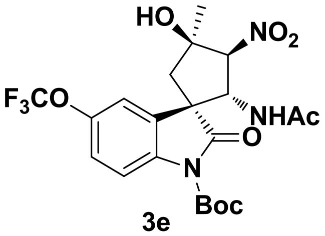

3e

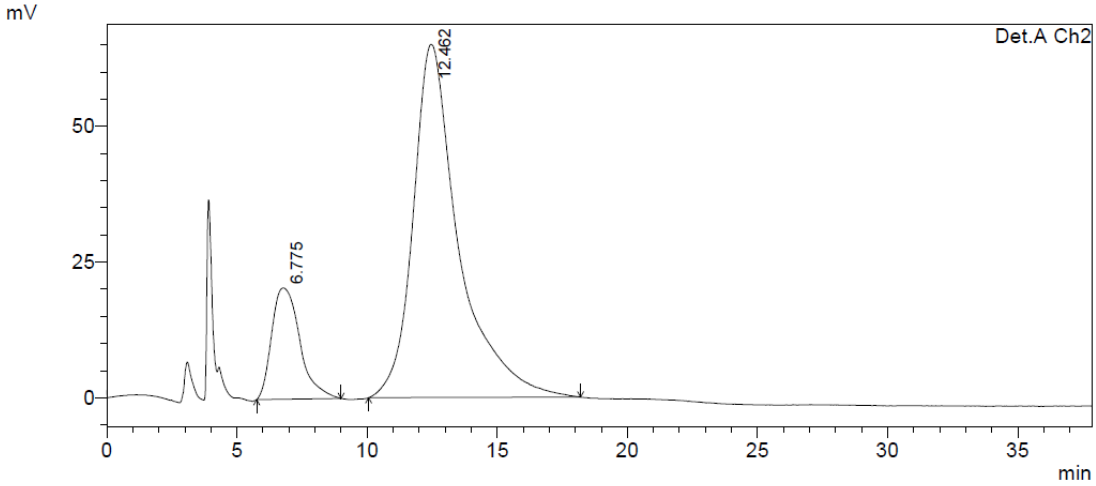

Peak#	Ret. Time	Area	Height	Area %	Height %
1	6.775	1541098	20499	16.314	23.967
2	12.462	7905113	65029	83.686	76.033
Total		9446210	85528	100.000	100.000

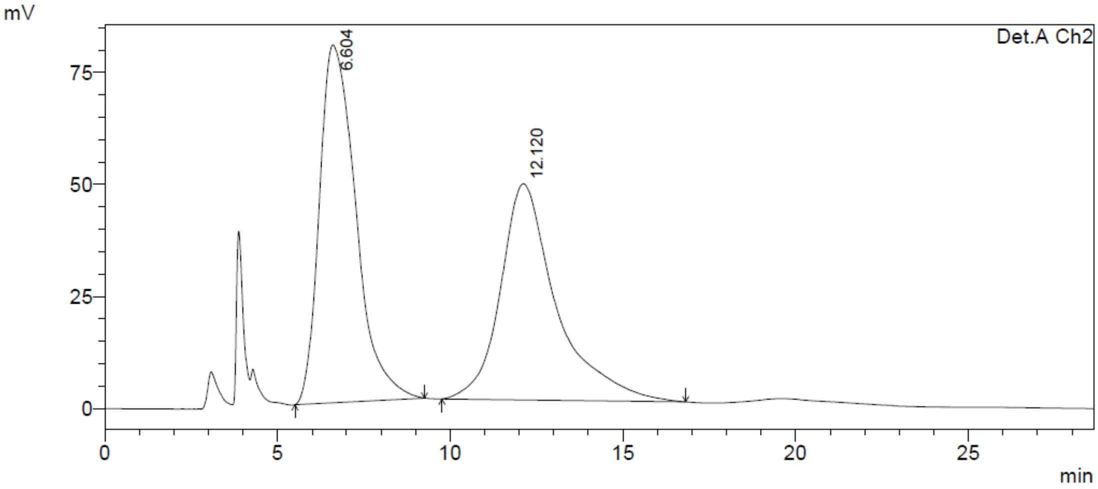

Peak#	Ret. Time	Area	Height	Area %	Height %
1	6.604	5865675	79784	51.936	62.338
2	12.120	5428404	48202	48.064	37.662
Total		11294079	127986	100.000	100.000

(1*S*,2*R*,3*S*,4*R*)-*tert*-Butyl-2-acetamido-4-hydroxy-5'-fluoro-4-methyl-3-nitro-2'-oxo-spiro[cyclopentane-1,3'-indoline]-1'-carboxylate **(3f)**

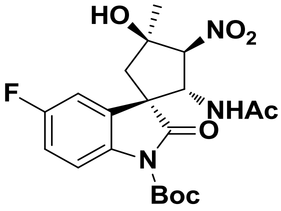

3f

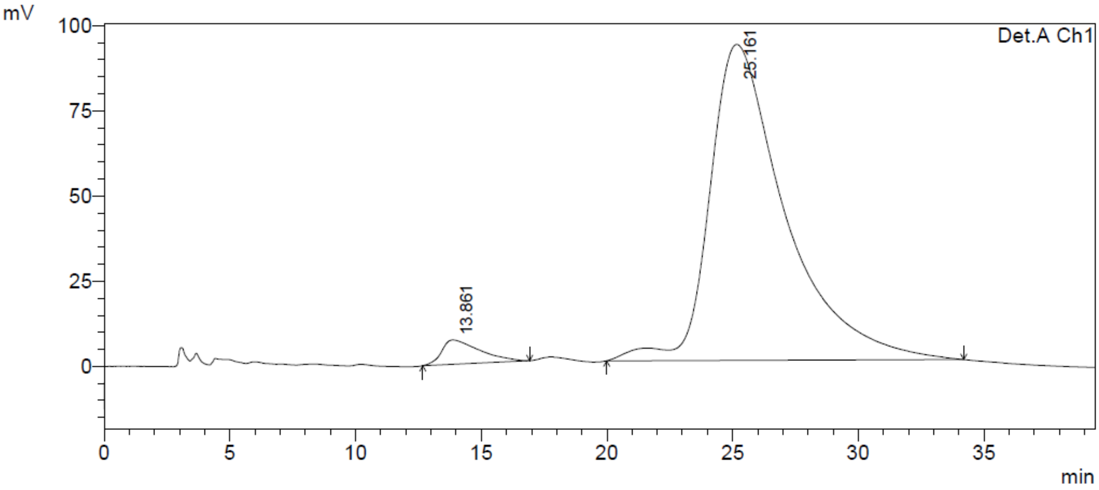

Peak#	Ret. Time	Area	Height	Area %	Height %
1	13.861	748876	7169	3.710	7.180
2	25.161	19436829	92687	96.290	92.820
Total		20185705	99856	100.000	100.000

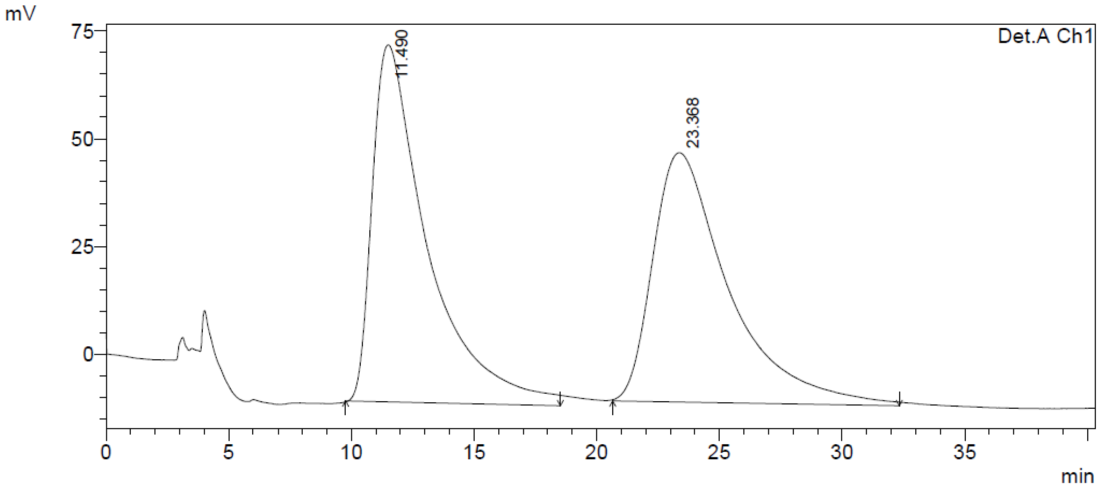

Peak#	Ret. Time	Area	Height	Area %	Height %
1	11.490	12910112	82888	50.931	58.880
2	23.368	12438224	57887	49.069	41.120
Total		25348336	140774	100.000	100.000

(1*S*,2*R*,3*S*,4*R*)-*tert*-Butyl-2-acetamido-4-hydroxy-5'-chloro-4-methyl-3-nitro-2'-oxo-spiro[cyclopentane-1,3'-indoline]-1'-carboxylate **(3g)**

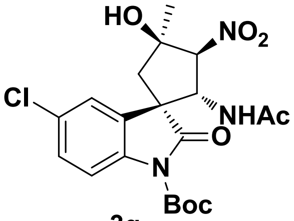

3g

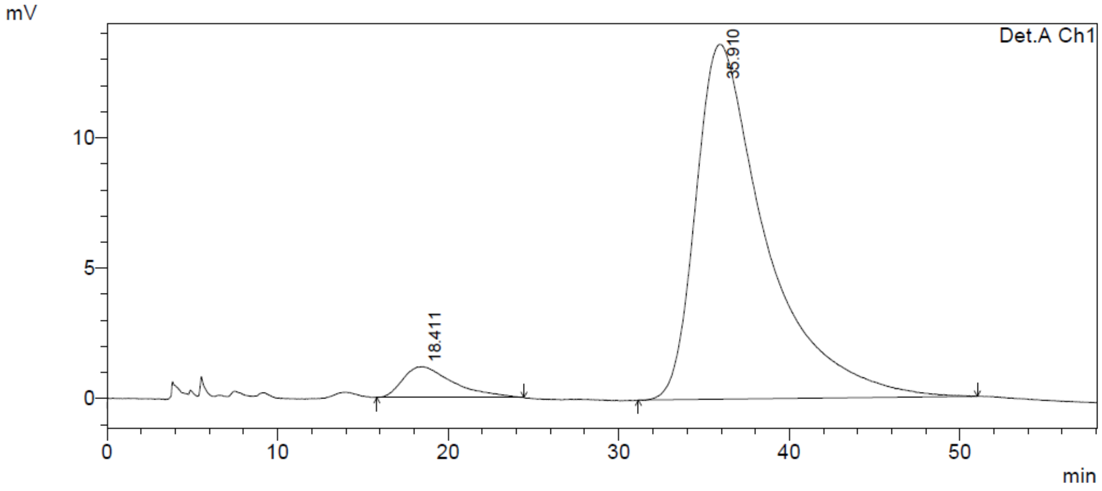

Peak#	Ret. Time	Area	Height	Area %	Height %
1	18.411	242954	1182	5.861	7.987
2	35.910	3902408	13617	94.139	92.013
Total		4145362	14799	100.000	100.000

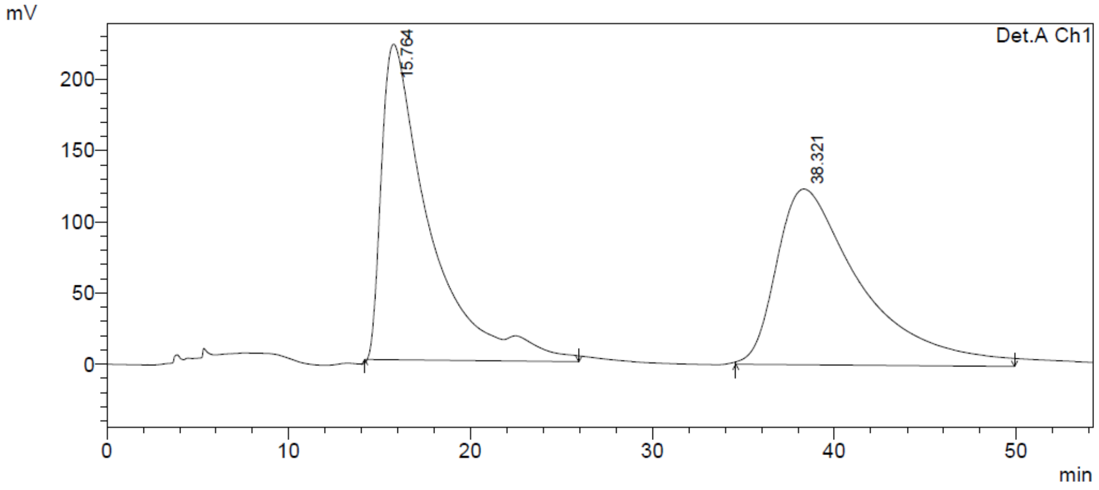

Peak#	Ret. Time	Area	Height	Area %	Height %
1	15.764	40805467	221593	50.435	64.239
2	38.321	40101122	123358	49.565	35.761
Total		80906590	344951	100.000	100.000

(1*S*,2*R*,3*S*,4*R*)-*tert*-Butyl-2-acetamido-4-hydroxy-7'-fluoro-4-methyl-3-nitro-2'-oxo-spiro[cyclopentane-1,3'-indoline]-1'-carboxylate **(3h)**

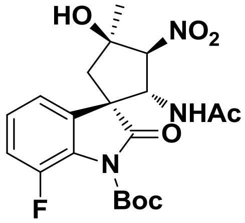

3h

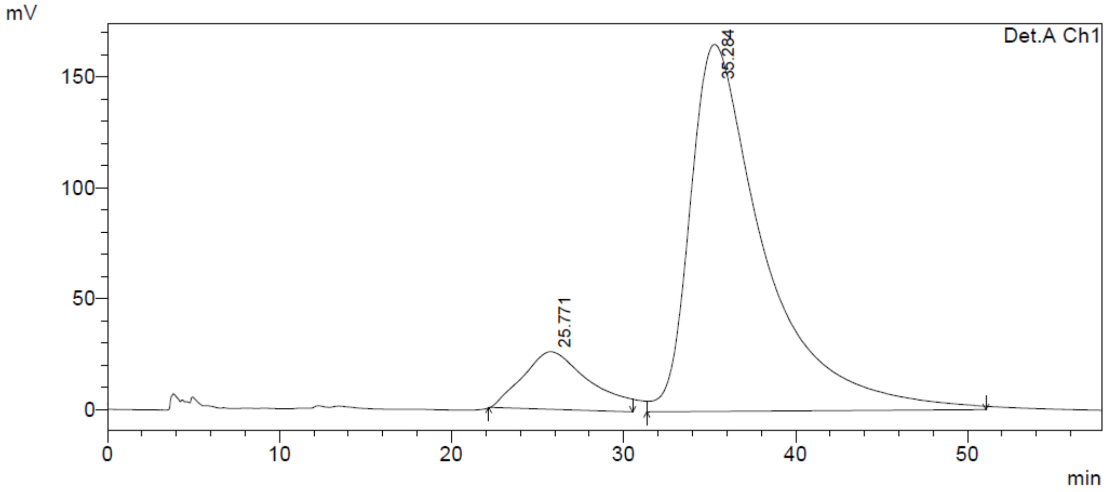

Peak#	Ret. Time	Area	Height	Area %	Height %
1	25.771	6939855	25940	12.222	13.544
2	35.284	49841992	165586	87.778	86.456
Total		56781846	191526	100.000	100.000

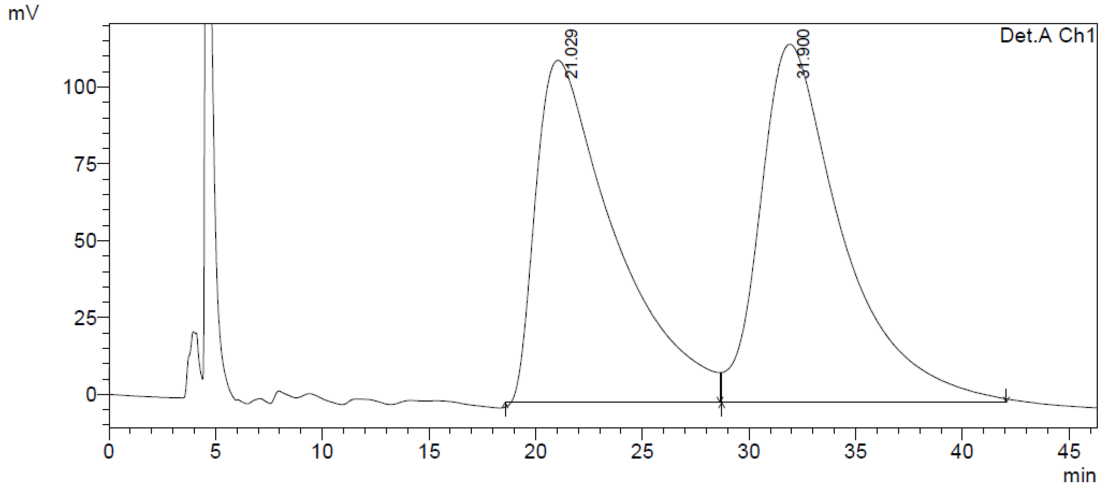

Peak#	Ret. Time	Area	Height	Area %	Height %
1	21.029	28809251	111285	47.879	48.852
2	31.900	31361384	116516	52.121	51.148
Total		60170635	227801	100.000	100.000

(1*S*,2*R*,3*S*,4*R*)-*tert*-Butyl-2-acetamido-4-hydroxy-6'-chloro-4-methyl-3-nitro-2'-oxo-spiro[cyclopentane-1,3'-indoline]-1'-carboxylate **(3i)**

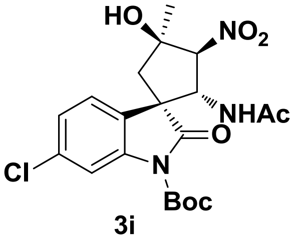

3i

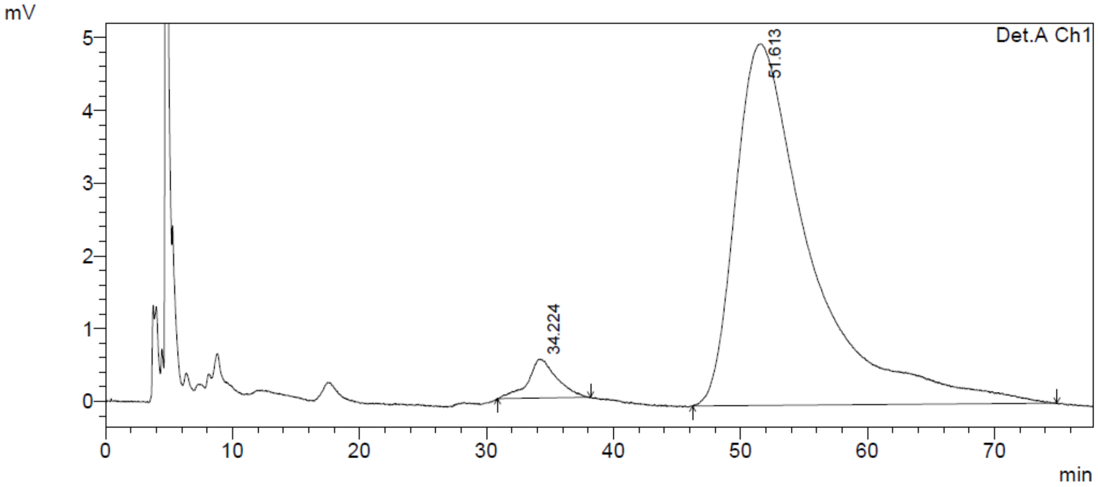

Peak#	Ret. Time	Area	Height	Area %	Height %
1	34.224	84934	534	3.971	9.707
2	51.613	2053697	4968	96.029	90.293
Total		2138631	5503	100.000	100.000

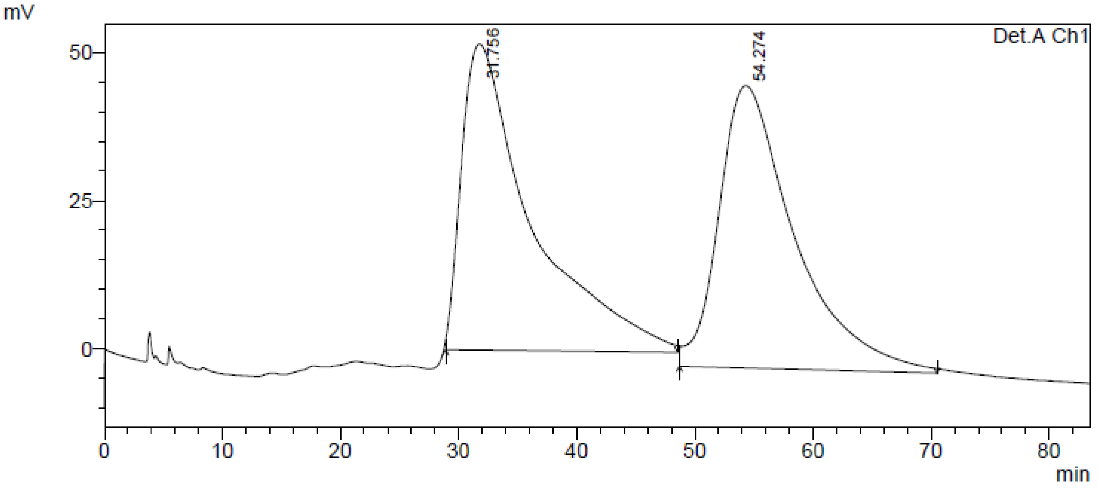

Peak#	Ret. Time	Area	Height	Area %	Height %
1	31.756	21228636	51556	49.904	52.020
2	54.274	21310440	47552	50.096	47.980
Total		42539076	99108	100.000	100.000

(1*S*,2*R*,3*S*,4*R*)-*tert*-Butyl-2-acetamido-4-hydroxy-6'-bromo-4-methyl-3-nitro-2'-oxo-spiro[cyclopentane-1,3'-indoline]-1'-carboxylate **(3j)**

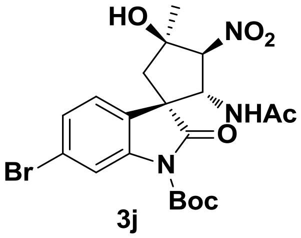

3j

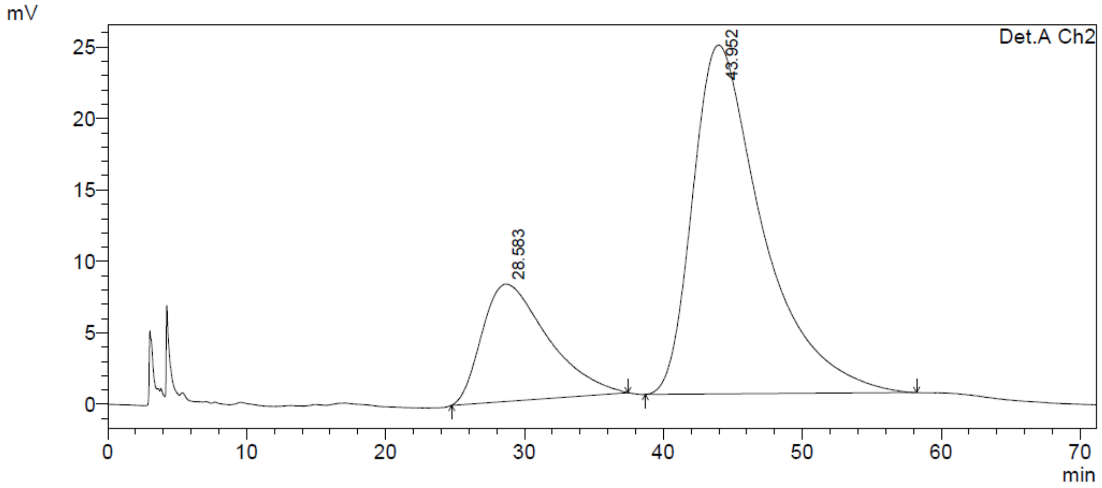

Peak#	Ret. Time	Area	Height	Area %	Height %
1	28.583	2697269	8213	23.972	25.155
2	43.952	8554474	24437	76.028	74.845
Total		11251743	32651	100.000	100.000

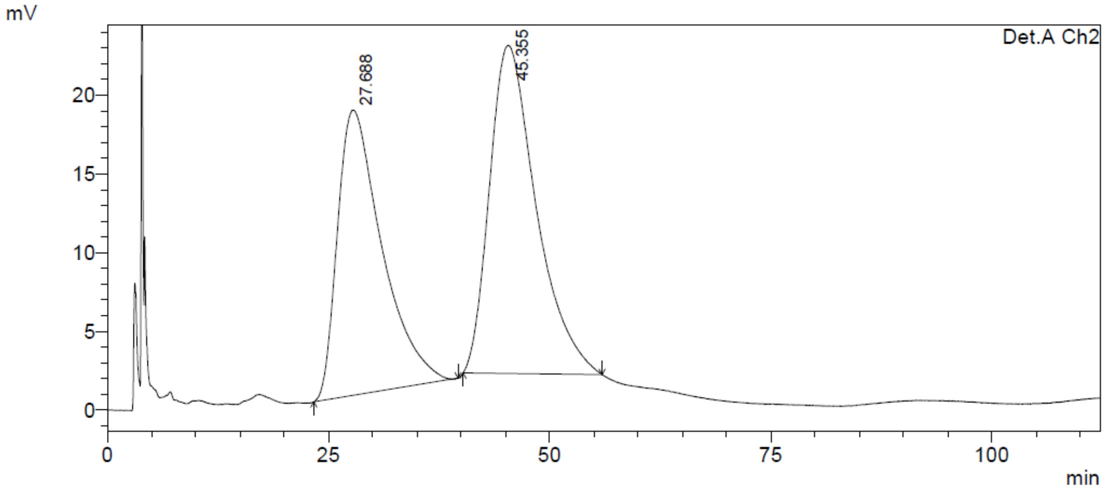

Peak#	Ret. Time	Area	Height	Area %	Height %
1	27.688	6327412	18146	45.211	46.528
2	45.355	7667794	20854	54.789	53.472
Total		13995206	38999	100.000	100.000

N-((1*S*,2*R*,3*S*,4*R*)-1'-Acetyl-3-hydroxy-3-methyl-4-nitro-2'-oxospiro[cyclopentane-1,3'-indolin]-5-yl)acetamide **(3k)**

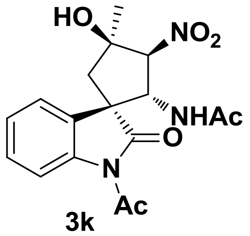

3k

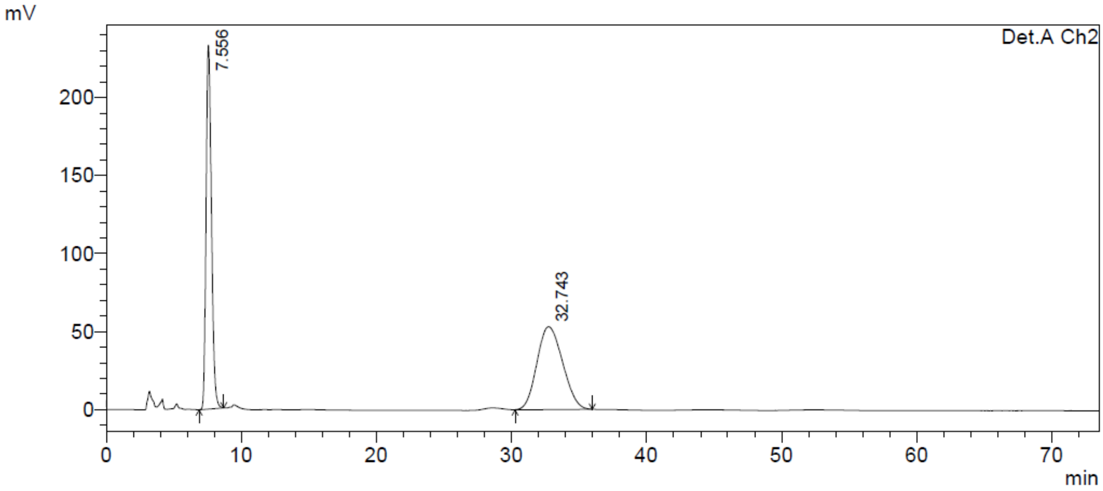

Peak#	Ret. Time	Area	Height	Area %	Height %
1	7.556	6440218	233019	47.878	81.455
2	32.743	7011057	53052	52.122	18.545
Total		13451275	286071	100.000	100.000

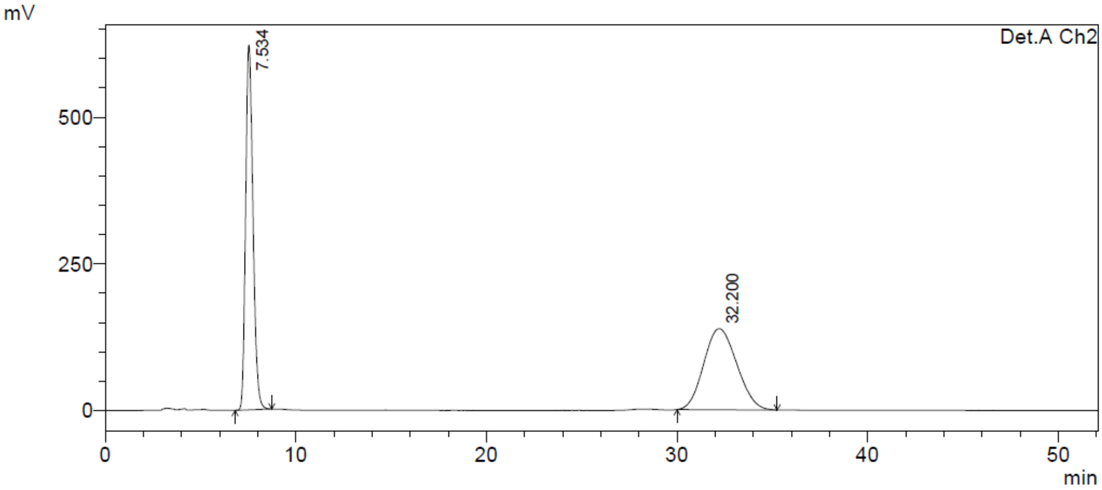

Peak#	Ret. Time	Area	Height	Area %	Height %
1	7.534	16716890	622199	50.021	81.810
2	32.200	16703063	138340	49.979	18.190
Total		33419952	760539	100.000	100.000

N-((1*S*,2*R*,3*S*,4*R*)-1'-Acetyl-3-hydroxy-3,5'-dimethyl-4-nitro-2'-oxospiro[cyclo-pentane-1,3'-indolin]-5-yl)acetamide **(3l)**

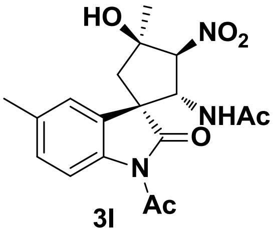

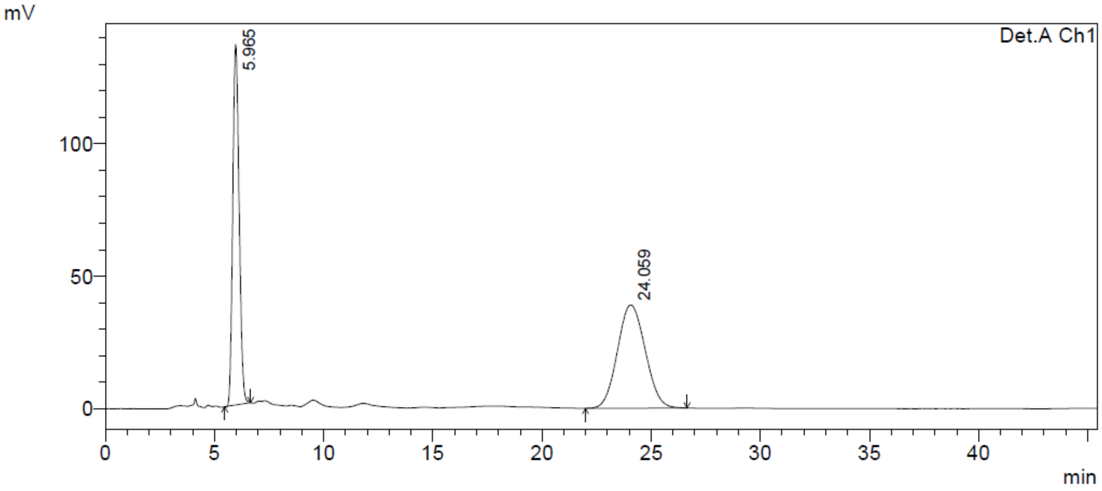

Peak#	Ret. Time	Area	Height	Area %	Height %
1	5.965	2756362	136055	44.462	77.751
2	24.059	3442964	38933	55.538	22.249
Total		6199326	174988	100.000	100.000

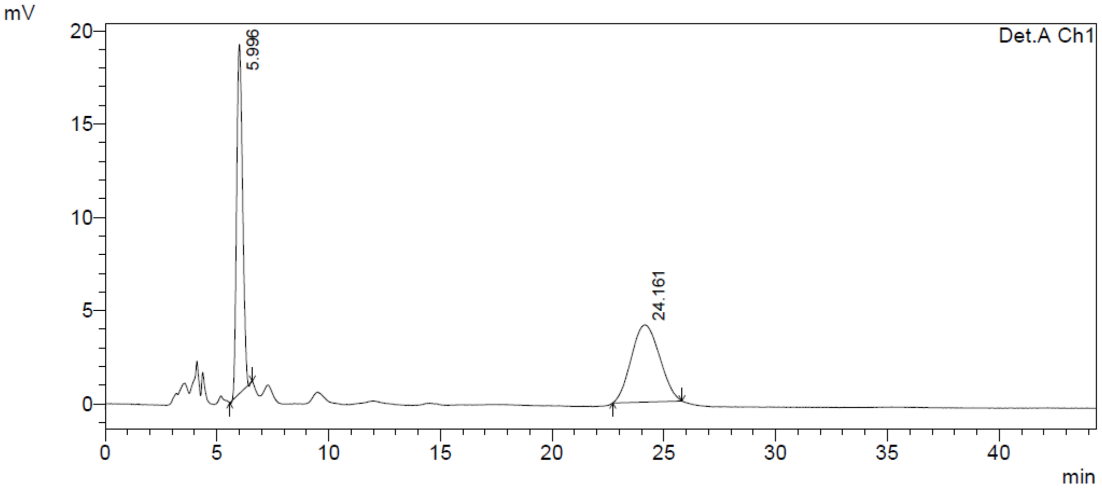

Peak#	Ret. Time	Area	Height	Area %	Height %
1	5.996	347747	18724	48.831	81.914
2	24.161	364400	4134	51.169	18.086
Total		712146	22858	100.000	100.000

N-((1*S*,2*R*,3*S*,4*R*)-1'-Acetyl-3-hydroxy-5'-methoxy-3-methyl-4-nitro-2'-oxospiro-[cyclopentane-1,3'-indolin]-5-yl)acetamide **(3m)**

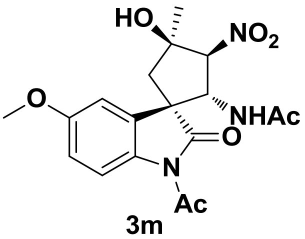

3m

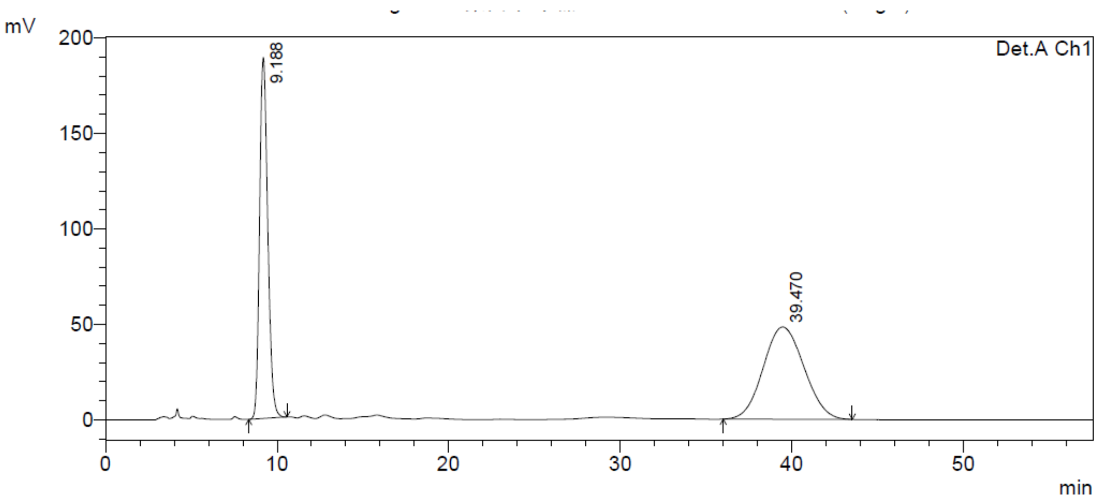

Peak#	Ret. Time	Area	Height	Area %	Height %
1	9.188	6277956	189080	43.983	79.645
2	39.470	7995643	48323	56.017	20.355
Total		14273598	237404	100.000	100.000

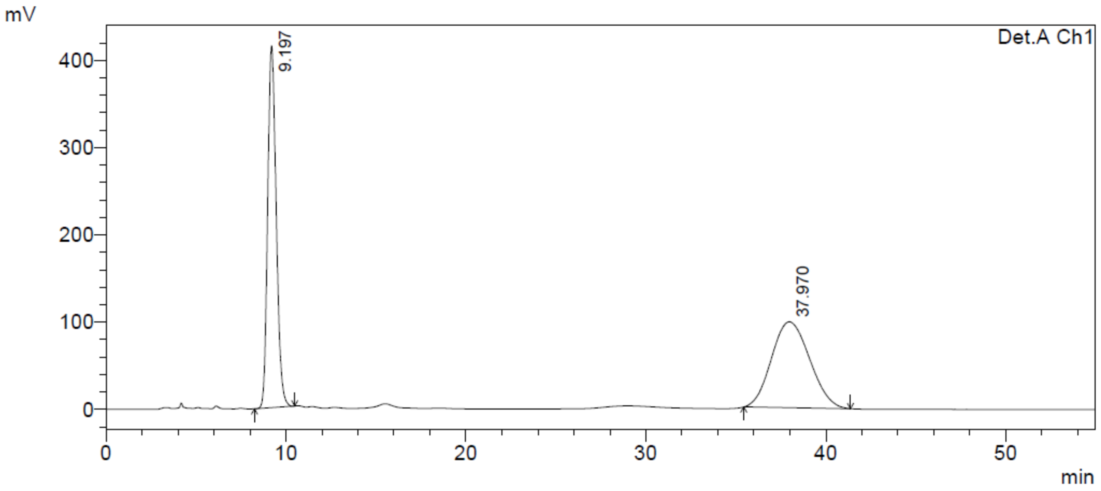

Peak#	Ret. Time	Area	Height	Area %	Height %
1	9.197	13690622	414836	48.476	80.826
2	37.970	14551504	98412	51.524	19.174
Total		28242126	513249	100.000	100.000

N-((1*S*,2*R*,3*S*,4*R*)-1'-Acetyl-5'-fluoro-3-hydroxy-3-methyl-4-nitro-2'-oxospiro[cyclopentane-1,3'-indolin]-5-yl)acetamide **(3n)**

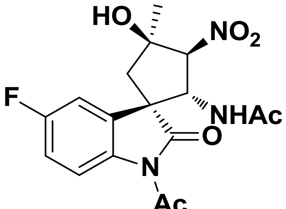

3n

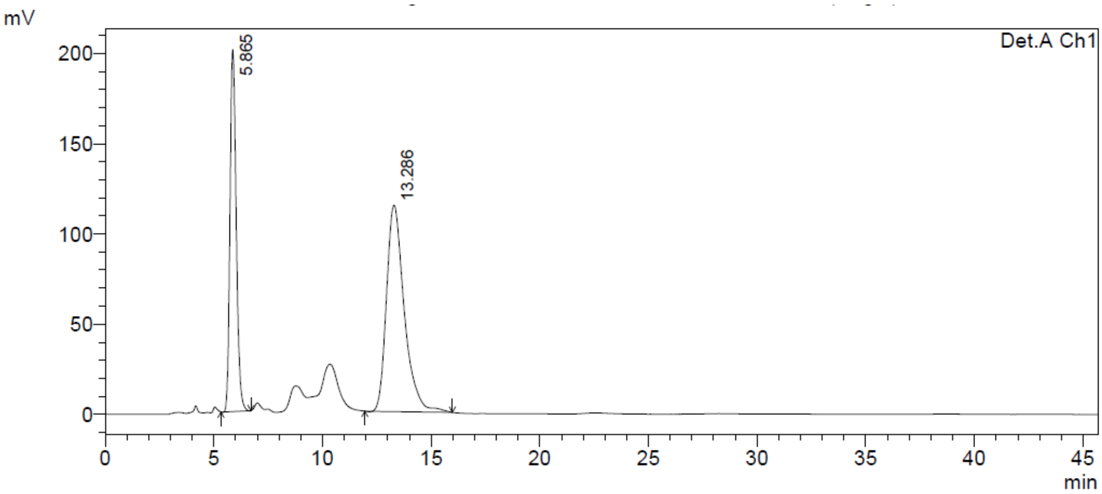

Peak#	Ret. Time	Area	Height	Area %	Height %
1	5.865	4006835	200622	38.108	63.676
2	13.286	6507453	114446	61.892	36.324
Total		10514289	315068	100.000	100.000

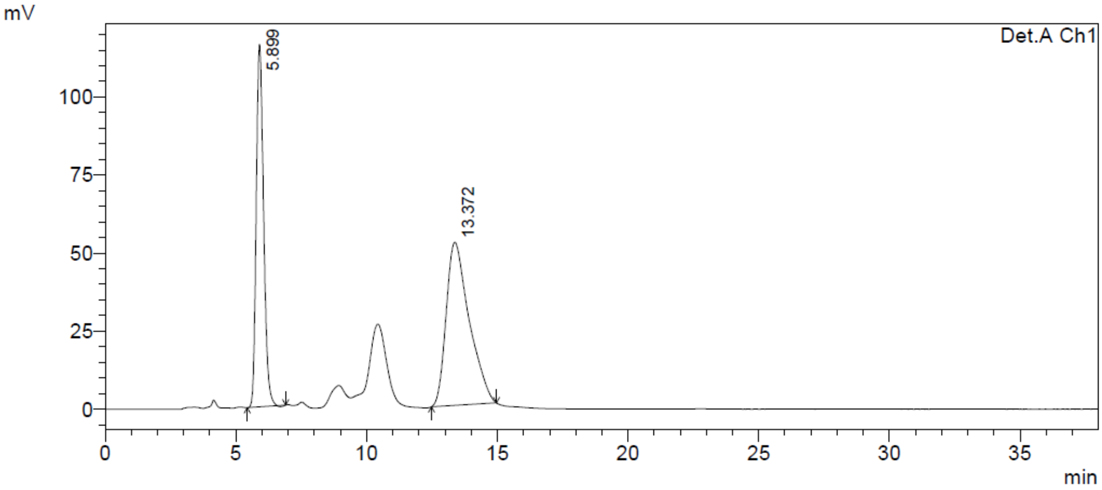

Peak#	Ret. Time	Area	Height	Area %	Height %
1	5.899	2264185	116089	41.482	68.977
2	13.372	3194090	52211	58.518	31.023
Total		5458275	168299	100.000	100.000

N-((1*S*,2*R*,3*S*,4*R*)-1'-Acetyl-7'-fluoro-3-hydroxy-3-methyl-4-nitro-2'-oxospiro[cyclo-pentane-1,3'-indolin]-5-yl)acetamide **(3o)**

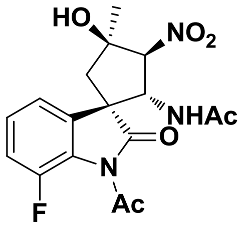

3o

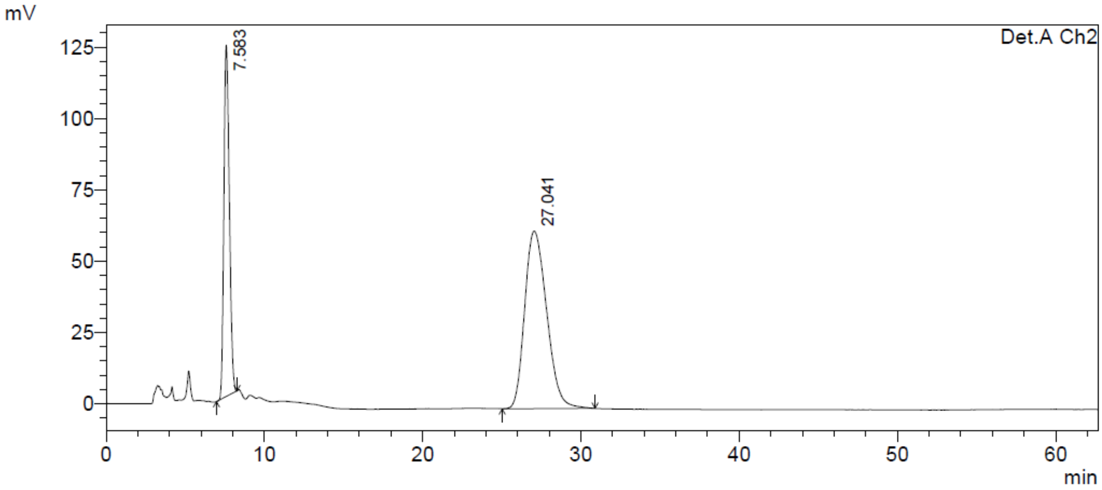

Peak#	Ret. Time	Area	Height	Area %	Height %
1	7.583	2952491	123258	32.879	66.446
2	27.041	6027459	62242	67.121	33.554
Total		8979950	185500	100.000	100.000

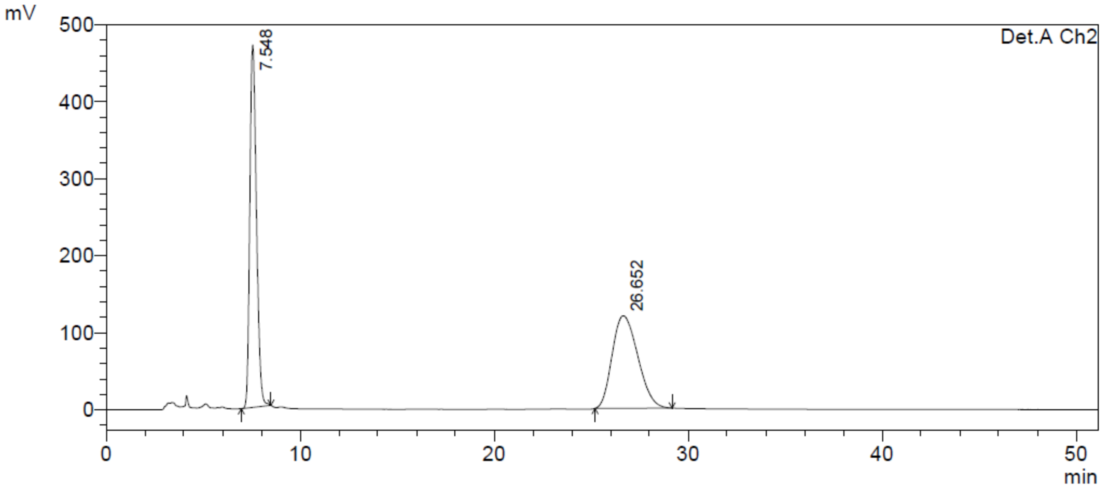

Peak#	Ret. Time	Area	Height	Area %	Height %
1	7.548	11217831	470609	50.364	79.647
2	26.652	11055880	120258	49.636	20.353
Total		22273712	590867	100.000	100.000

(D) X-ray Data

Figure S1: X-ray Structure of compound **3g**.

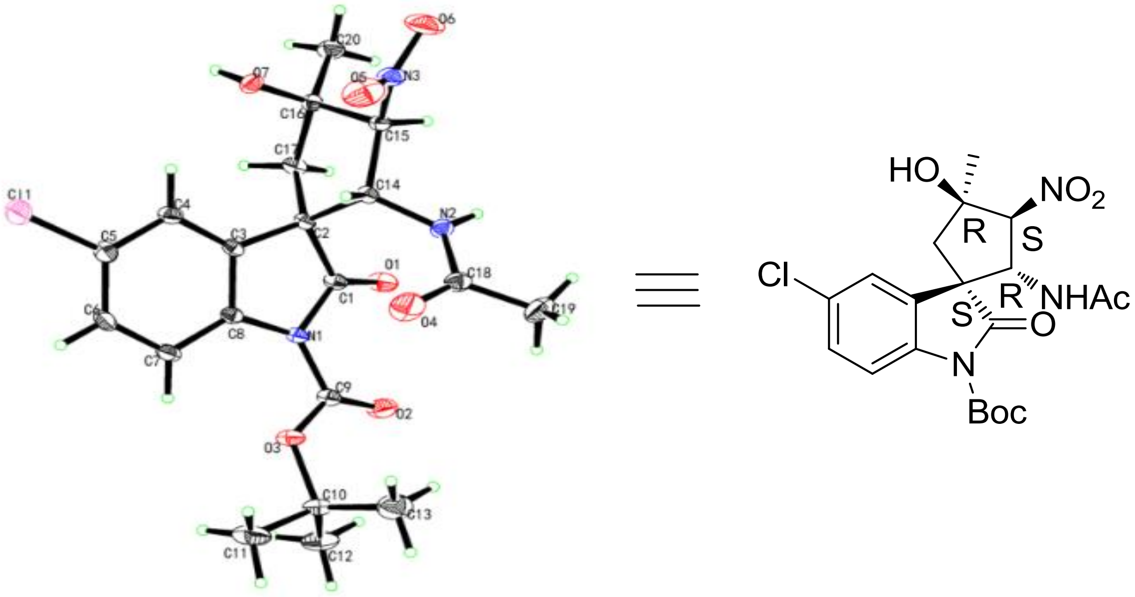

The (1*S*,2*R*,3*S*,4*R*)-configuration of **3g** was determined by the X-ray analysis of its crystal. The structure was solved and refined using the Bruker SHELXTL Software Package. X-ray crystallographic data of (1*S*,2*R*,3*S*,4*R*)-**3g** were collected at T = 296 K: $C_{20}H_{24}ClN_3O_7$, *M*r = 453.87. Space group: *P*212121, a = 9.7678 (9) Å, b = 14.5518 (14) Å, c = 15.4811 (15) Å, $\alpha = 90°$, $\beta = 90°$, $\gamma = 90°$, V = 2200.5 (4) Å^3, Z = 4.

CCDC 1532974 (**3g**) contains the supplementary crystallographic data for this paper. These data can be obtained free of charge from the Cambridge Crystallographic Data Centre via www.ccdc.cam.ac.uk/data_request/cif.
